# Associations between Dietary Pulses Alone or with Other Legumes and Cardiometabolic Disease Outcomes: An Umbrella Review and Updated Systematic Review and Meta-analysis of Prospective Cohort Studies

**DOI:** 10.1093/advances/nmz113

**Published:** 2019-11-15

**Authors:** Effie Viguiliouk, Andrea J Glenn, Stephanie K Nishi, Laura Chiavaroli, Maxine Seider, Tauseef Khan, Marialaura Bonaccio, Licia Iacoviello, Sonia Blanco Mejia, David J A Jenkins, Cyril W C Kendall, Hana Kahleová, Dario Rahelić, Jordi Salas-Salvadó, John L Sievenpiper

**Affiliations:** 1 Toronto 3D Knowledge Synthesis and Clinical Trials Unit, St. Michael's Hospital, Toronto, Ontario, Canada; 2 Department of Nutritional Sciences, University of Toronto, Toronto, Ontario, Canada; 3 Clinical Nutrition and Risk Factor Modification Centre, St. Michael's Hospital, Toronto, Ontario, Canada; 4 Department of Epidemiology and Prevention, IRCCS Neuromed, Pozzilli, Italy; 5 Research Center in Epidemiology and Preventive Medicine (EPIMED), Department of Medicine and Surgery, University of Insubria, Varese, Italy; 6 Department of Medicine, University of Toronto, Toronto, Ontario, Canada; 7 Division of Endocrinology & Metabolism, St. Michael's Hospital, Toronto, Ontario, Canada; 8 Li Ka Shing Knowledge Institute, St. Michael's Hospital, Toronto, Ontario, Canada; 9 College of Pharmacy and Nutrition, University of Saskatchewan, Saskatoon, Saskatchewan, Canada; 10 Physicians Committee for Responsible Medicine, Washington, DC, USA; 11 Institute for Clinical and Experimental Medicine, Prague, Czech Republic; 12 Department of Endocrinology, Diabetes and Clinical Pharmacology, Dubrava University Hospital, School of Medicine, University of Zagreb, Zagreb, Croatia; 13 CIBER Fisiopatología de la Obesidad y Nutrición (CIBER Obn), Instituto de Salud Carlos III, Madrid, Spain; 14 Human Nutrition Department, IISPV, Universitat Rovira i Virgili, Reus, Spain

**Keywords:** pulses, legumes, cardiovascular disease, diabetes, hypertension, obesity, prospective cohort, systematic review, meta-analysis, GRADE

## Abstract

To update the European Association for the Study of Diabetes clinical practice guidelines for nutrition therapy, we conducted an umbrella review and updated systematic review and meta-analysis (SRMA) of prospective cohort studies of the association between dietary pulses with or without other legumes and cardiometabolic disease outcomes. We searched the PubMed, MEDLINE, EMBASE, and Cochrane databases through March 2019. We included the most recent SRMAs of prospective cohort studies and new prospective cohort studies published after the census dates of the included SRMAs assessing the relation between dietary pulses with or without other legumes and incidence and mortality of cardiovascular diseases (CVDs) [including coronary heart disease (CHD), myocardial infarction (MI), and stroke], diabetes, hypertension, and/or obesity. Two independent reviewers extracted data and assessed risk of bias. Risk estimates were pooled using the generic inverse variance method and expressed as risk ratios (RRs) with 95% CIs. The overall certainty of the evidence was assessed using the GRADE approach. Six SRMAs were identified and updated to include 28 unique prospective cohort studies with the following number of cases for each outcome: CVD incidence, 10,261; CVD mortality, 16,168; CHD incidence, 7786; CHD mortality, 3331; MI incidence, 2585; stroke incidence, 8570; stroke mortality, 2384; diabetes incidence, 10,457; hypertension incidence, 83,284; obesity incidence, 8125. Comparing the highest with the lowest level of intake, dietary pulses with or without other legumes were associated with significant decreases in CVD (RR: 0.92; 95% CI: 0.85, 0.99), CHD (RR: 0.90; 95% CI: 0.83, 0.99), hypertension (RR: 0.91; 95% CI: 0.86, 0.97), and obesity (RR: 0.87; 95% CI: 0.81, 0.94) incidence. There was no association with MI, stroke, and diabetes incidence or CVD, CHD, and stroke mortality. The overall certainty of the evidence was graded as “low” for CVD incidence and “very low” for all other outcomes. Current evidence shows that dietary pulses with or without other legumes are associated with reduced CVD incidence with low certainty and reduced CHD, hypertension, and obesity incidence with very low certainty. More research is needed to improve our estimates. This trial was registered at clinicaltrials.gov as NCT03555734.

## Introduction

Dietary pulses, the edible dried seeds of legumes (i.e., chickpeas, lentils, beans, and peas) that are high in fiber, plant protein, and various micronutrients and low in fat and glycemic index (GI) (1–3), have been increasingly recognized for their benefits in the prevention and management of type 2 diabetes and cardiovascular diseases (CVDs) across various chronic disease guidelines. The American Heart Association, Canadian Cardiovascular Society, and European Society for Cardiology encourage dietary patterns that emphasize intake of legumes (which include dietary pulses, soybeans, peanuts, fresh peas, and fresh beans) for lowering LDL cholesterol and blood pressure (4), dietary pulses for lowering LDL cholesterol (5), and legumes for lowering LDL cholesterol and improving the overall lipoprotein profile (6), respectively. Similarly, diabetes guidelines from Diabetes Canada recommend that individuals with diabetes consume dietary pulses to help manage glycemic control, blood pressure, and body weight (7) and the American Diabetes Association recommend various dietary patterns that include dietary pulses as acceptable for the management of diabetes (8). Although the European Association for the Study of Diabetes (EASD) recommend legumes to help meet minimum requirements for fiber intake (9), they have not yet assessed the evidence for the prevention and management of type 2 diabetes and CVD.

To update the recommendations for the role of dietary pulses in the prevention and management of cardiometabolic diseases, the Diabetes and Nutrition Study Group of the EASD commissioned a series of systematic reviews and meta-analyses (SRMAs) using the Grading of Recommendations Assessment, Development and Evaluation (GRADE) approach. The present SRMA using the GRADE approach was conducted to address the question of whether the available evidence from prospective cohort studies of dietary pulses with or without other legumes shows advantages for CVDs and other cardiometabolic disease outcomes.

## Methods

We conducted an umbrella review and updated SRMA (study protocol: NCT03555734) following the methodology from the *Cochrane Handbook for Systematic Reviews and Interventions* (10). Reporting followed the Meta-analysis of Observational Studies in Epidemiology guideline (11) and Preferred Reporting Items for Systematic Reviews and Meta-Analyses guideline (www.prisma-statement.org).

### Data sources and searches

For the umbrella review, we updated our search from a previous umbrella review (12) from 9 December, 2016 through to 14 March, 2019 using PubMed (which includes the MEDLINE and National Library of Medicine databases) and the following search terms: “pulses” OR “legumes” AND “meta-analysis.”

For the updated SRMA, we searched MEDLINE, EMBASE, and the Cochrane Library databases for new prospective cohort studies published after the census dates of the SRMAs identified in the umbrella review through March 2019. The search strategies are presented in **Supplemental Tables 1–4**. The search was restricted to human studies without language restrictions. Manual searches of the reference lists of included studies supplemented electronic searches.

### Study selection

For the umbrella review, we included the most recent SRMAs of prospective cohort studies assessing the relation between dietary pulses with or without other legumes (if dietary pulses alone were not reported) and incidence and/or mortality of cardiometabolic disease outcomes (including CVDs, diabetes, hypertension, and/or obesity). Multiple SRMAs that assessed the same outcome were included when they consisted of different studies.

For the updated SRMA, we included prospective cohort studies published after the census dates of the SRMAs identified from the umbrella review. Studies were included if they were of ≥1-y follow-up duration and assessed the association between dietary pulses with or without other legumes (if dietary pulses alone were not reported) and incidence and/or mortality of cardiometabolic disease outcomes (including CVDs, diabetes, hypertension, and/or obesity) in people free of the disease at baseline.

### Data extraction

Two reviewers (EV, AG, SKN, LC, or MS) independently reviewed the articles and extracted relevant data. The primary outcome was incidence and/or mortality of CVDs [including CVD, coronary heart disease (CHD), myocardial infarction (MI), and stroke] and secondary outcomes included incidence and/or mortality of diabetes, hypertension, and obesity expressed as risk ratios (RRs) with 95% CIs. We contacted authors for missing data (13–15).

### Risk of bias

The Newcastle-Ottawa Scale (NOS) was used to assess the risk of bias in included studies, where ≤9 points were awarded based on cohort selection (≤4 points), the comparability of the cohort (≤2 points), and adequacy of the outcome measures (≤3 points) (16). Studies achieving ≥6 points were considered low risk of bias. Differences were reconciled by consensus.

### Grading of the evidence

The certainty and strength of the evidence were assessed using the GRADE approach (17–29). Included observational studies started at low-certainty evidence by default and then were downgraded or upgraded based on prespecified criteria. Criteria to downgrade included study limitations (the weight of studies showed risk of bias by the NOS), inconsistency (substantial unexplained interstudy heterogeneity, *I*^2^ ≥ 50% and *P* < 0.10), indirectness (presence of factors relating to the population, exposures, and outcomes that limit generalizability), imprecision [95% CIs were wide or crossed a minimally important difference of 5% (RR: 0.95–1.05) for all outcomes], and publication bias (significant evidence of small-study effects). Criteria to upgrade included a large size effect (RR >2 or RR <0.5 in the absence of plausible confounders), a dose–response gradient, and attenuation by plausible confounding effects.

### Statistical analyses

Primary and sensitivity analyses were conducted using Review Manager version 5.3 (The Cochrane Collaboration). Subgroup and publication bias analyses were conducted using STATA software, version 13.0 (StataCorp LLC). Individual cohort comparison RRs from the most adjusted models were obtained comparing the extreme quantiles. ORs and HRs were regarded as RRs. When studies used continuous relative risk per dose, we imputed the extreme quantile RRs by obtaining dose difference from relevant data provided by the study in the same or another publication, or using the most similar study taking into account location, population, time, and age. To obtain summary estimates, we ln-transformed the RRs and pooled them using DerSimonian–Laird random-effects models (30). A fixed-effects model was used when data from <5 studies were available.

Heterogeneity was assessed (Cochran *Q* statistic) and quantified (*I*^2^ statistic). If *I*^2^ was ≥50% and *P* < 0.10, we interpreted this as indicating substantial heterogeneity (10, 24). We also investigated possible sources of heterogeneity through sensitivity and subgroup analyses. Sensitivity analyses were performed by systematically removing each study from the meta-analysis with recalculation of the summary estimates in order to assess whether any single study exerted an undue influence on the summary estimates. If ≥10 cohort comparisons were available, a priori subgroup analyses were conducted for sex, follow-up, validation of dietary assessment methods, NOS, and funding source using meta-regression analyses. A post hoc subgroup or sensitivity analysis was performed (depending on whether there were ≥10 or <10 cohort comparisons available, respectively) for each outcome to assess the association in studies reporting dietary pulses alone as the exposure. A random-effects linear dose-response was modelled using a generalized least-square trend for estimation of summarized dose-response data as per Greenland and Longnecker (31) and Orsini et al. (32). A 2-stage multivariate random-effects method was used to model a nonlinear association using restricted cubic splines with 3 knots (32). If ≥10 cohort comparisons were available, we investigated publication bias by visual inspection of funnel plots and using the Begg (33) and Egger tests (34).

## Results

**Supplemental Figure 1** shows the flow of the literature for the umbrella review. We identified 6 SRMAs: 3 for CVDs (35–37), 1 for diabetes (38), 1 for hypertension (39), and 1 for obesity outcomes (40). **Supplemental Figures 2–5** show the flow of the literature for the updated search of these SRMAs. Ten new prospective cohort studies were identified for CVDs (13–15, 41–47), 2 for diabetes (48, 49), 1 for hypertension (50), and 0 for obesity outcomes. The total number of cohort comparisons included from the identified SRMAs and our updated search were 7 for CVD incidence (231,353 unique participants and 10,261 cases) (42, 44, 51–54), 12 for CVD mortality (940,756 unique participants and 16,186 cases) (13–15, 43–47, 52, 55, 56), 10 for CHD incidence (306,814 unique participants and 7786 cases) (51, 57–62), 9 for CHD mortality (224,592 unique participants and 3331 cases) (14, 41, 43, 45, 55, 60, 63, 64), 4 for MI incidence (202,528 unique participants, 2585 cases) (44, 52, 63), 8 for stroke incidence (342,079 unique participants and 8570 cases) (44, 52, 65–68), 6 for stroke mortality (168,504 unique participants and 2384 cases) (14, 41, 43, 45, 55, 67), 9 for diabetes incidence (259,325 unique participants and 10,457 cases) (48, 53, 69–73), 7 for hypertension incidence (288,352 unique participants and 83,284 cases) (50, 74–77), and 1 for obesity/overweight incidence (78).

### Study characteristics

**Table 1** and **Supplemental Tables 5–14** show the characteristics of the included prospective cohort studies. Participants were from several geographical areas including Asia, Europe, the Middle East, North America, and Oceania and tended to be middle-aged. Based on available data, there were more female than male participants across all outcomes. The median follow-up durations ranged from 6 y for diabetes incidence to 22 y for stroke incidence. Ascertainment of incident cases was done by medical records across all outcomes, with the exceptions of diabetes, hypertension, and obesity incidence, where there were some studies using self-report (43%, 54%, and 100%, respectively). The percentage of studies reporting dietary pulses alone as the exposure ranged from 13% for CHD mortality and diabetes incidence to 100% for MI incidence. Dietary intake was assessed by some form of FFQ by the majority of studies. The lowest quantile of intake from dietary pulses with or without other legumes ranged from a median of 0 g/d for MI incidence to 16.2 g/d for obesity incidence. The highest quantile of intake from dietary pulses with or without other legumes ranged from a median of 27.8 g/d for CVD mortality to 213 g/d for MI incidence. All studies were funded by agency alone except for 5 studies that were funded by both agency and industry (14, 15, 44, 48, 73) and 2 studies where funding sources were unknown (13, 47, 49, 54).

**TABLE 1:** Summary of characteristics of prospective cohort studies assessing the associations between dietary pulses with or without other legumes and cardiometabolic disease outcomes in participants free of the disease at baseline^1^

	Cardiometabolic disease outcome									
Characteristic	CVD incidence	CVD mortality	CHD incidence	CHD mortality	MI incidence	Stroke incidence	Stroke mortality	Diabetes incidence	Hypertension incidence	Obesity incidence
Cohorts, *n*	6	11	8	8	3	7	6	8	7	1
Cohort comparisons, *n*	7	12	10	9	4	8	6	9	7	1
Geographic regions (cohorts, *n*)	Asia (1)	Asia (2)	Asia (2)	Asia (2)	Asia (1)	Asia (1)	Asia (2)	Asia (1)	Europe (2)	North America
	Europe (2)	Europe (5)	Europe (3)	Europe (3)	North America (1)	Europe (2)	Europe (3)	Europe (3)	North America (4)	
	Middle East (1)	Middle East (1)	North America (3)	Middle East (1)	Several regions^2^ (1)	North America (3)	Middle East (1)	Middle East (1)	Middle East (1)	
	North America (1)	North America (1)		North America (2)		Several regions^2^ (1)		North America (2)		
	Several regions^2^ (1)	Oceania (1)						Oceania (1)		
		Several regions^2^ (1)								
Unique participants, *n*	231,353	940,756	306,814	224,592	202,528	342,079	168,504	259,325	288,352	18,146
Men:women, %	42:58	37:63	31:69	36:64	42:58	40:60	43:57	10:90	21:79	0:100
Median age (range), y	51 (18–101)	57 (35–85)	54 (18–101)	56 (20–86)	52 (35–70)	54 (30–75)	57 (35–85)	55 (20–80)	52 (18–90)	≥45
Median follow-up (range), y	9 (7–19)	9 (6–16)	10 (5–26)	9 (6–26)	10 (6–13)	22 (7–26)	9 (7–26)	6 (4–18)	9 (3–26)	16
Cases, *n*	10,261	16,186	7786	3331	2585	8570	2384	10,457	83,284	8125
Outcome assessment methods (cohorts, *n*)	Medical records (6)	Medical records (11)	Medical records (8)	Medical records (8)	Medical records (3)	Medical records (7)	Medical records (6)	Medical records (4)	Medical records (3)	Self-report
								Self-report (3)	Self-report (4)	
								NR (1)		
Exposure^3^ (cohorts, *n*)	Pulses (3)	Pulses (3)	Pulses (2)	Pulses (1)	Pulses (3)	Pulses (2)	Pulses (1)	Pulses (1)	Pulses (5)	Pulses + other legumes
	Pulses + other legumes (3)	Pulses + other legumes (8)	Pulses + other legumes (6)	Pulses + other legumes (7)	Pulses + other legumes (0)	Pulses + other legumes (5)	Pulses + other legumes (5)	Pulses + other legumes (7)	Pulses + other legumes (2)	
Diet assessment methods (cohorts, *n*)	FFQ (1)	FFQ (1)	FFQ (1)	FFQ (1)	vFFQ (2)	vFFQ (2)	FFQ (1)	FFQ (1)	vSFFQ (4)	vSFFQ
	vFFQ (2)	vFFQ (3)	vSFFQ (2)	vFFQ (1)	vSFFQ (1)	vSFFQ (2)	vFFQ (1)	vSFFQ (3)	SFFQ, interviewer administered (1)	
	vSFFQ (3)	vFFQ, interviewer administered (1)	SFFQ, interviewer administered (1)	vSFFQ (3)		SFFQ, interviewer administered (1)	vSFFQ (1)	vSFFQ, interviewer administered (3)	vSFFQ, interviewer administered (1)	
		vSFFQ (2)	vSFFQ, interviewer administered (4)	vSFFQ, interviewer administered (3)		vSFFQ, interviewer administered (1)	vSFFQ, interviewer administered (3)	Modified diet history method (1)	24-h dietary record (1)	
		vSFFQ, interviewer administered (3)				Diet history interview (1)				
		Several methods^4^ (1)								
Median lowest quantile of dietary pulse or legume intake (range), g/d	5.9 (0.0–8.4)	5.0 (0.0–14)	8.5 (0.0–20.9)	7.0 (3.0–10.7)	0 (—)	6.8 (0.0–7.6)	5.7 (3.0–8.3)	7.3 (0.0–20.2)	2.0 (0.0–13.5)	16.2
Median highest quantile of dietary pulse or legume intake (range), g/d	80.9 (36.0–213)	27.8 (25.0–213)	62.8 (16.4–295.6)	43.0 (26.0–74.7)	213 (—)	55.3 (40.3–213)	32.1 (26.0–53.8)	46.9 (28.8–125.4)	75.2 (43.9–162.8)	75.8
Funding sources^5^ (cohorts, *n*)	Agency (4)	Agency (6)	Agency (8)	Agency (7)	Agency (2)	Agency (6)	Agency (5)	Agency (5)	Agency (7)	Agency
	Agency–industry (1)	Agency–industry (3)		Agency–industry (1)	Agency–industry (1)	Agency–industry (1)	Agency–industry (1)	Agency–industry (2)		
	NR (1)	NR (2)						NR (1)		

1 CHD, coronary heart disease; CVD, cardiovascular disease; MI, myocardial infarction; NR, not reported; SFFQ, semiquantitative FFQ; vFFQ, validated FFQ; vSFFQ, validated semiquantitative FFQ.

2 The PURE cohort study (44) was conducted in Canada, Sweden, United Arab Emirates, Argentina, Brazil, Chile, Malaysia, Poland, South Africa, Turkey, China, Colombia, Iran, occupied Palestinian territory, Bangladesh, India, Pakistan, and Zimbabwe.

3 Studies included under “Pulses” included those reporting only chickpeas, lentils, beans, and/or peas in the exposure. Studies included under “Pulses + other legumes” included those reporting “legumes” without differentiating the legume type or which included other types of legumes in the exposure in addition to pulses (e.g., soybeans, soy products, peanuts, fresh peas, and/or fresh beans). (Note: if a study included beans and peas in the exposure and did not specify whether they were fresh and/or dry, the study was categorized under “Pulses.”)

4 Several validated dietary assessment tools including SFFQs, FFQs, quantitative dietary questionnaires, and/or food records.

5 Agency funding is that from government, university, or not-for-profit sources. Industry funding is that from trade organizations that obtain revenue from the sale of products.

**Supplemental Tables 15–24** show the statistical adjustments performed in the included studies. All studies adjusted for the prespecified primary confounding variables (age for the majority of outcomes) with the exception of 1 study (49). Fewer than half the studies assessing CVD outcomes (48%) adjusted for ≥7 of the 9 secondary confounding variables for CVD outcomes (sex, family history of CVD, smoking, markers of overweight/obesity, diabetes, hypertension, dyslipidemia, energy intake, and physical activity). The majority of the studies assessing diabetes outcomes (88%) adjusted for ≥4 of the 6 secondary confounding variables for diabetes outcomes (sex, family history of diabetes, smoking, markers of overweight/obesity, energy intake, and physical activity). The majority of the studies assessing hypertension outcomes (86%) adjusted for ≥5 of the 7 secondary confounding variables for hypertension outcomes (sex, diabetes, smoking, markers of overweight/obesity, energy intake, sodium intake, and physical activity).

### Risk of bias assessment

**Supplemental Table 25** shows the NOS scores for the included prospective cohort studies. Although several studies lost points in several domains, there was no evidence of serious risk of bias across the included studies assessing CVD outcomes, diabetes, and obesity incidence. For hypertension incidence, >50% of the weight (68.7%) was contributed by studies considered to be high risk of bias (NOS <6).

### Dietary pulses with or without other legumes and CVD incidence

**FIGURE 1:**
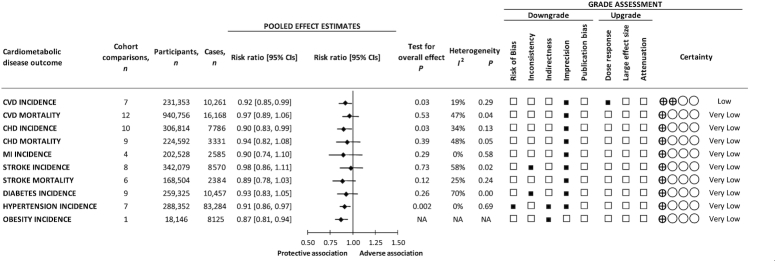
Summary and GRADE assessment of the pooled effect estimates of prospective cohort studies assessing the associations between dietary pulses with or without other legumes and cardiometabolic disease outcomes (the highest compared with the lowest level of intake) in participants free of the disease at baseline. Pooled risk estimate for each outcome is represented by the diamond. Data are expressed as weighted risk ratios with 95% CIs using the generic inverse-variance method modelled by random effects, or by fixed effects if data from <5 studies were available. Values of *I*^2^ ≥ 50% and *P* < 0.10 indicate substantial heterogeneity (10, 24). Values >1.0 indicate an adverse association. CHD, coronary heart disease; CVD, cardiovascular disease; GRADE, Grading of Recommendations Assessment, Development and Evaluation; MI, myocardial infarction; NA, not available.

**Figure 1** and **Supplemental Figure 6** show the association between dietary pulses with or without other legumes and CVD incidence (7 cohort comparisons, 231,353 unique participants, and 10,261 cases). We found a protective association (RR: 0.92; 95% CI: 0.85, 0.99; *P* = 0.03) with no evidence of substantial heterogeneity (*I*^2^ = 19%, *P* = 0.29) when we compared the highest and lowest levels of intake.

### Dietary pulses with or without other legumes and CVD mortality

Figure 1 and **Supplemental Figure 7** show the association between dietary pulses with or without other legumes and CVD mortality (12 cohort comparisons, 940,756 unique participants, and 16,168 cases). There was no association (RR: 0.97; 95% CI: 0.89, 1.06; *P* = 0.53) with no evidence of substantial heterogeneity (*I*^2^ = 47%, *P* = 0.04) when we compared the highest and lowest levels of intake.

### Dietary pulses with or without other legumes and CHD incidence

Figure 1 and **Supplemental Figure 8** show the association between dietary pulses with or without other legumes and CHD incidence (10 cohort comparisons, 306,814 unique participants, and 7786 cases). We found a protective association (RR: 0.90; 95% CI: 0.83, 0.99; *P* = 0.03) with no evidence of substantial heterogeneity (*I*^2^ = 34%, *P* = 0.13) when we compared the highest and lowest levels of intake.

### Dietary pulses with or without other legumes and CHD mortality

Figure 1 and **Supplemental Figure 9** show the association between dietary pulses with or without other legumes and CHD mortality (9 cohort comparisons, 224,592 unique participants, and 3331 cases). There was no association (RR: 0.94; 95% CI: 0.82, 1.08; *P* = 0.39) with no evidence of substantial heterogeneity (*I*^2^ = 48%, *P* = 0.05) when we compared the highest and lowest levels of intake.

### Dietary pulses with or without other legumes and MI incidence

Figure 1 and **Supplemental Figure 10** show the association between dietary pulses with or without other legumes and MI incidence (4 cohort comparisons, 202,528 unique participants, and 2585 cases). There was no association (RR: 0.90; 95% CI: 0.74, 1.10; *P* = 0.29) with no evidence of heterogeneity (*I*^2^ = 0%, *P* = 0.58) when we compared the highest and lowest levels of intake.

### Dietary pulses with or without other legumes and stroke incidence

Figure 1 and **Supplemental Figure 11** show the association between dietary pulses with or without other legumes and stroke incidence (8 cohort comparisons, 342,079 unique participants, and 8570 cases). There was no association (RR: 0.98; 95% CI: 0.86, 1.11; *P* = 0.73) with evidence of substantial heterogeneity (*I*^2^ = 58%, *P* = 0.02) when we compared the highest and lowest levels of intake.

### Dietary pulses with or without other legumes and stroke mortality

Figure 1 and **Supplemental Figure 12** show the association between dietary pulses with or without other legumes and stroke mortality (6 cohort comparisons, 168,504 unique participants, and 2384 cases). There was no association (RR: 0.89; 95% CI: 0.78, 1.03; *P* = 0.12) with no evidence of substantial heterogeneity (*I*^2^ = 25%, *P* = 0.24) when we compared the highest and lowest levels of intake.

### Dietary pulses with or without other legumes and diabetes incidence

Figure 1 and **Supplemental Figure 13** show the association between dietary pulses with or without other legumes and diabetes incidence (9 cohort comparisons, 259,325 unique participants, and 10,457 cases). There was no association (RR: 0.93; 95% CI: 0.83, 1.05; *P* = 0.26) with evidence of substantial heterogeneity (*I*^2^ = 70%, *P* = 0.0008) when we compared the highest and lowest levels of intake.

### Dietary pulses with or without other legumes and hypertension incidence

Figure 1 and **Supplemental Figure 14** show the association between dietary pulses with or without other legumes and hypertension incidence (7 cohort comparisons, 288,352 unique participants, and 83,284 cases). We found a protective association (RR: 0.91; 95% CI: 0.86, 0.97; *P* = 0.002) with no evidence of heterogeneity (*I*^2^ = 0%, *P* = 0.69) when we compared the highest and lowest levels of intake.

### Dietary pulses with or without other legumes and obesity incidence

Only 1 cohort study was identified that assessed the association between legumes and overweight/obesity incidence (78), which showed a protective association (RR: 0.87; 95% CI: 0.81, 0.97; *P*-trend < 0.0001) when comparing the highest and lowest levels of intake.

### Sensitivity, subgroup, and dose-response analyses

**Supplemental Table 26** shows select sensitivity analyses in which the systematic removal of an individual study altered the significance of the pooled effect estimate or evidence of heterogeneity for an outcome. The systematic removal of several studies modified the association between dietary pulses with or without other legumes and incidence of CVD (42, 44, 51, 52, 54) and CHD (51, 58, 60) from a protective association to no association. The systematic removal of Bonaccio et al. (14) modified the association between dietary pulses with or without other legumes and stroke mortality from no association to a protective association. The systematic removal of several studies modified the heterogeneity from nonsubstantial to substantial for the association between dietary pulses with or without other legumes and mortality of CVD (13, 44, 45, 47, 52) and CHD (45, 55, 60, 63, 64).

**Supplemental Figures 15** and **16** show the a priori and post hoc subgroup analyses. CVD mortality and CHD incidence were the only outcomes with ≥10 cohort comparisons available, both of which showed no evidence of effect modification by any of the subgroups for the association with dietary pulses with or without other legumes.

**Supplemental Table 27** shows the post hoc sensitivity analyses assessing the association in those studies reporting dietary pulses alone as the exposure. Of the outcomes with <10 cohort comparisons available, none showed an association with dietary pulses after removal of studies including other legumes in the exposure, with the exception of hypertension incidence (RR: 0.92; 95% CI: 0.87, 0.98; *P* = 0.01).

**Supplemental Figure 17**A–H shows the dose-response analyses. Only studies with intake data were included, which consisted of 3 for CVD incidence, 7 for CVD mortality, 7 for CHD incidence, 4 for CHD mortality, 5 for stroke incidence, 3 for stroke mortality, 6 for diabetes incidence, and 6 for hypertension incidence. There was evidence of a linear dose-response gradient (per 100 g) for dietary pulses with or without other legumes and CVD incidence (RR: 0.92; 95% CI: 0.86, 0.98; *P* = 0.007). No other outcomes showed evidence of a linear or nonlinear dose-response gradient.

### Publication bias

**Supplemental Figures 18** and **19** show the publication bias analyses. CVD mortality and CHD incidence were the only outcomes with ≥10 cohort comparisons available, both of which showed no evidence of publication bias through visual inspection of funnel plots and formal testing with Begg and Egger tests.

### GRADE assessment

Figure 1 and **Supplemental Table 28** show the GRADE assessments for the associations between dietary pulses with or without legumes and each cardiometabolic disease outcome. The evidence for benefit was rated as very low certainty for CHD and MI incidence and CVD, CHD, and stroke mortality owing to downgrades for serious imprecision; very low certainty for stroke and diabetes incidence owing to downgrades for inconsistency and imprecision; very low certainty for hypertension incidence owing to downgrades for risk of bias, indirectness, and imprecision; very low certainty for obesity incidence owing to downgrades for indirectness; and low certainty for CVD incidence owing to a downgrade for imprecision and an upgrade for a significant inverse dose-response gradient.

## Discussion

We conducted an umbrella review and updated SRMA of prospective cohort studies assessing the association between dietary pulses with or without other legumes and cardiometabolic disease outcomes. We identified 6 SRMAs and updated their search, which resulted in the following total number of cohort comparisons for each outcome: 7 for CVD incidence, 12 for CVD mortality, 10 for CHD incidence, 9 for CHD mortality, 4 for MI incidence, 8 for stroke incidence, 6 for stroke mortality, 9 for diabetes incidence, 7 for hypertension incidence, and 1 for obesity incidence. Pooled analyses showed that dietary pulses with or without other legumes were associated with an 8%, 10%, 9%, and 13% decrease in CVD, CHD, hypertension, and obesity incidence, respectively, when comparing the highest quantile of intake with the lowest quantile of intake. No association was found between dietary pulses with or without other legumes and incidence of MI, stroke, and diabetes or mortality from CVD, CHD, and stroke.

### Results in relation to other studies

Our results are consistent with previous SRMAs of prospective cohort studies in this area that were identified through our umbrella review (35–40), as well as with SRMAs of randomized trials of corresponding risk factors for these disease outcomes, including blood lipids (79), glycemic control (80), blood pressure (81), body weight, and adiposity (82). Potential mechanisms for these findings have been discussed in more detail in a previous umbrella review published by our group (12). Briefly, the potential mechanisms for the observed benefits for the incidence of CVD, CHD, hypertension, and obesity may be mediated by the effects of specific nutrients and properties found in dietary pulses and other legumes, including their high fiber, magnesium, potassium, and protein contents and being low in GI (12). The inconsistencies observed between risk of incidence of and mortality from CVD and CHD are not entirely clear. It is also not clear why benefits were observed for incident CVD and CHD but not incident stroke. It is possible that the benefit observed for incident CVD is being driven by the benefit observed for incident CHD. However, given that the 95% CIs still include benefit for all these outcomes (incident stroke and mortality from CVD and CHD), the benefit of dietary pulses on these outcomes cannot be excluded. More precise estimates are needed to better understand the relation between dietary pulses and their impact on cardiometabolic disease outcomes.

### Strengths and limitations

The strengths of our study are that we identified all available prospective cohort studies through a systematic search strategy, performed quantitative syntheses, and conducted an assessment of the certainty of the evidence by using the GRADE approach.

Despite the inclusion of several large high-quality cohorts, the inability to rule out residual confounding is a limitation inherent in all observational studies, and a reason that observational studies start at low certainty when assessed by GRADE. Sources of residual confounding include reverse causality, the reliability of self-reported intake (83), measured and unmeasured confounders included in statistical models, and important collinearity effects from related dietary and lifestyle patterns. Other important limitations include risk of bias, inconsistency between studies, and indirectness. Risk of bias could not be ruled out for hypertension incidence because half of the studies were considered high risk of bias (contributing 68.7% weight in the pooled analysis) and residual inconsistency could not be ruled out for stroke and diabetes incidence owing to substantial unexplained interstudy heterogeneity (*I*^2^ ≥ 50%, *P* < 0.10). Indirectness could not be ruled out for hypertension incidence because half of the studies were conducted in health professionals (contributing 68.7% weight in the pooled analysis). Although many of the studies specified “legumes” as the exposure without differentiating the legume types or included other legumes in the exposure (e.g., soy, soy products, peanuts), we did not downgrade for indirectness. This is because >50% of the weight was contributed by studies conducted in North America and Europe across the majority of the cardiometabolic disease outcomes. Available data suggest that a higher percentage of individuals consume dietary pulses than consume soy and soy products in North America (1, 2, 84, 85) and dietary patterns commonly consumed in Europe (e.g., Mediterranean, Nordic dietary patterns) typically include or emphasize dietary pulses (86–88). Another limitation consists of the wide range of intake of dietary pulses with or without other legumes across studies within the lowest and highest quantiles of intake, which makes it difficult to ascertain an optimum intake level for health benefits. A final limitation is the imprecision in the estimates of pooled risk. The 95% CIs were wide and could not rule out clinically important benefit or harm across the majority of cardiometabolic disease outcomes. In addition, there was some instability in the precision of the summary estimates for incidence of CVD and CHD and stroke mortality. Lastly, there was only 1 prospective cohort study identified assessing the relation between intake of dietary pulses with or without other legumes and obesity risk.

Balancing the strengths and weaknesses, the evidence was assessed as very low certainty for CHD and MI incidence and mortality from CVD, CHD, and stroke owing to downgrades for serious imprecision; very low certainty for stroke and diabetes incidence owing to downgrades for inconsistency and imprecision; very low certainty for hypertension incidence owing to downgrades for risk of bias, indirectness, and imprecision; very low certainty for obesity incidence owing to downgrades for indirectness; and low certainty for CVD incidence owing to an upgrade for a significant inverse dose-response gradient.

### Implications and future directions

Current levels of dietary pulse consumption remain low, for it has been reported that only 13% of Canadians (2) and 7.9% of Americans (1) consume dietary pulses on any given day. Among consumers, meanintakes ranged from 13 to 294 g/d among Canadians (2) and from 23 to 277 g/d among Americans (1) (approximately <0.25 to 1.75 cups/d). European data show a similar pattern of low consumption (89). Given this low level of consumption there is room to incorporate dietary pulses as part of a healthy dietary pattern to improve cardiometabolic health. We found benefits of intake levels for dietary pulses with or without other legumes ranging from a median of 62.8 g/d for CHD incidence to 80.9 g/d for CVD incidence, which is in line with the levels used in randomized controlled trials showing benefits of dietary pulses on cardiometabolic risk factors (12, 79–82). Furthermore, consumption of dietary pulses has been shown to have larger societal implications, including the potential to lower annual health care costs (90) and contribute to environmental sustainability (91), which is a growing global concern.

More research is needed in this area to improve our understanding of the impact of dietary pulses on cardiometabolic health. In specific, future studies should differentiate between legume types in the exposure and independently analyze the association between dietary pulses and cardiometabolic outcomes in order to improve our understanding.

### Conclusions

Overall, our umbrella review and updated SRMA of available prospective cohort studies supports the intake of dietary pulses with or without other legumes in the prevention of some cardiometabolic diseases (CVD, CHD, hypertension, and obesity). Our confidence in the evidence for this conclusion is generally weak or very weak. Sources of uncertainty include the risk of residual confounding in observational studies that prevent causal inferences from being drawn, serious inconsistency between studies, indirect measurement of dietary pulses, and imprecision in estimates of pooled risk. More research is likely to have an important influence on our estimates and increase our understanding of the role of dietary pulses in the primary prevention of CVDs and other cardiometabolic outcomes.

## Supplementary Material

nmz113_Supplemental_File

## Notes

Aspects of this work were presented at the 7th International Congress on Vegetarian Nutrition, Loma Linda, CA, USA, 26–28 February 2018, and the 36th International Symposium on Diabetes and Nutrition, Opatija, Croatia, 27–30 June 2018.

Supported by Canadian Institutes of Health Research grant 129920 through the Canada-wide Human Nutrition Trialists’ Network. The Diabetes and Nutrition Study Group (DNSG) of the European Association for the Study of Diabetes (EASD) commissioned this systematic review and meta-analysis and provided funding and logistical support for meetings as part of their development of clinical practice guidelines for nutrition therapy. The Diet, Digestive tract, and Disease (3D) Centre, funded through the Canada Foundation for Innovation and the Ministry of Research and Innovation's Ontario Research Fund, provided the infrastructure for the conduct of this project. EV was supported by a Toronto 3D Knowledge Synthesis and Clinical Trials Foundation Internship Award. MB was supported by a Fondazione Umberto Veronesi Fellowship and by Italian Ministry of Health grant GR-2013-02356060. DJAJ was funded by the Government of Canada through the Canada Research Chair Endowment. JLS was funded by a PSI Graham Farquharson Knowledge Translation Fellowship, Diabetes Canada Clinician Scientist Award, Canadian Institutes of Health Research (CIHR) New Investigator Partnership Prize, and Banting & Best Diabetes Centre (BBDC) Sun Life Financial New Investigator Award for Diabetes Research.

Author disclosures: EV and AJG serve as scientific advisors for New Era Nutrition. SKN worked as a clinical research dietitian at Glycaemic Index Laboratories, Toronto, Ontario, Canada, and is currently a registered dietitian and study sub-coordinator at Diabetes Heart Research Centre for clinical trials of AstraZeneca, Boehringer Ingelheim, Novo Nordisk, and Sanofi. LC worked as a clinical research coordinator at Glycaemic Index Laboratories, Toronto, Ontario, Canada. TK has received research support from the Canadian Institutes of Health Research (CIHR) and an unrestricted travel donation from Bee Maid Honey Ltd. He has been an invited speaker at the Calorie Control Council Annual meeting for which he has received an honorarium. DJAJ has received research grants from Saskatchewan & Alberta Pulse Growers Associations, the Agricultural Bioproducts Innovation Program through the Pulse Research Network, the Advanced Foods and Material Network, Loblaw Companies Ltd, Unilever Canada and Netherlands, Barilla, the Almond Board of California, Agriculture and Agri-food Canada, Pulse Canada, Kellogg's Company, Canada, Quaker Oats, Canada, Procter & Gamble Technical Centre Ltd, Bayer Consumer Care, Springfield, NJ, Pepsi/Quaker, International Nut & Dried Fruit (INC), Soy Foods Association of North America, the Coca-Cola Company (investigator initiated, unrestricted grant), Solae, Haine Celestial, the Sanitarium Company, Orafti, the International Tree Nut Council Nutrition Research and Education Foundation, the Peanut Institute, Soy Nutrition Institute (SNI), the Canola and Flax Councils of Canada, the Calorie Control Council, the Canadian Institutes of Health Research (CIHR), the Canada Foundation for Innovation (CFI), and the Ontario Research Fund (ORF). He has received in-kind supplies for trials as a research support from the Almond Board of California, Walnut Council of California, American Peanut Council, Barilla, Unilever, Unico, Primo, Loblaw Companies, Quaker (Pepsico), Pristine Gourmet, Bunge Limited, Kellogg Canada, WhiteWave Foods. He has been on the speaker's panel, served on the scientific advisory board and/or received travel support and/or honoraria from the Almond Board of California, Canadian Agriculture Policy Institute, Loblaw Companies Ltd, the Griffin Hospital (for the development of the NuVal scoring system), the Coca-Cola Company, EPICURE, Danone, Diet Quality Photo Navigation (DQPN), Better Therapeutics (FareWell), Verywell, True Health Initiative (THI), Institute of Food Technologists (IFT), Soy Nutrition Institute (SNI), Herbalife Nutrition Institute (HNI), Saskatchewan & Alberta Pulse Growers Associations, Sanitarium Company, Orafti, the American Peanut Council, the International Tree Nut Council Nutrition Research and Education Foundation, the Peanut Institute, Herbalife International, Pacific Health Laboratories, Nutritional Fundamentals for Health (NFH), Barilla, Metagenics, Bayer Consumer Care, Unilever Canada and Netherlands, Solae, Kellogg, Quaker Oats, Procter & Gamble, Abbott Laboratories, Dean Foods, the California Strawberry Commission, Haine Celestial, PepsiCo, the Alpro Foundation, Pioneer Hi-Bred International, DuPont Nutrition and Health, Spherix Consulting and WhiteWave Foods, the Advanced Foods and Material Network, the Canola and Flax Councils of Canada, Agri-Culture and Agri-Food Canada, the Canadian Agri-Food Policy Institute, Pulse Canada, the Soy Foods Association of North America, the Nutrition Foundation of Italy (NFI), Nutra-Source Diagnostics, the McDougall Program, the Toronto Knowledge Translation Group (St. Michael's Hospital), the Canadian College of Naturopathic Medicine, The Hospital for Sick Children, the Canadian Nutrition Society (CNS), the American Society of Nutrition (ASN), Arizona State University, Paolo Sorbini Foundation, and the Institute of Nutrition, Metabolism and Diabetes. He received an honorarium from the United States Department of Agriculture to present the 2013 W.O. Atwater Memorial Lecture. He received the 2013 Award for Excellence in Research from the International Nut and Dried Fruit Council. He received funding and travel support from the Canadian Society of Endocrinology and Metabolism to produce mini cases for the Canadian Diabetes Association (CDA). He is a member of the International Carbohydrate Quality Consortium (ICQC). His wife, Alexandra L Jenkins, is a director and partner of Glycemic Index Laboratories, Inc., and his sister, Caroline Brydson, received funding through a grant from the St. Michael's Hospital Foundation to develop a cookbook for one of his studies. CWCK has received grants or research support from the Advanced Food Materials Network, Agriculture and Agri-Foods Canada (AAFC), Almond Board of California, American Peanut Council, Barilla, Canadian Institutes of Health Research (CIHR), Canola Council of Canada, International Nut and Dried Fruit Council, International Tree Nut Council Research and Education Foundation, Loblaw Brands Ltd, Pulse Canada, and Unilever. He has received in-kind research support from the Almond Board of California, American Peanut Council, Barilla, California Walnut Commission, Kellogg Canada, Loblaw Companies, Quaker (PepsiCo), Primo, Unico, Unilever, WhiteWave Foods/Danone. He has received travel support and/or honoraria from the American Peanut Council, Barilla, California Walnut Commission, Canola Council of Canada, General Mills, International Nut and Dried Fruit Council, International Pasta Organization, Loblaw Brands Ltd, Nutrition Foundation of Italy, Oldways Preservation Trust, Paramount Farms, Peanut Institute, Pulse Canada, Sun-Maid, Tate & Lyle, Unilever, and White Wave Foods/Danone. He has served on the scientific advisory board for the International Tree Nut Council, International Pasta Organization, McCormick Science Institute, and Oldways Preservation Trust. He is a member of the International Carbohydrate Quality Consortium (ICQC), Executive Board Member of the Diabetes and Nutrition Study Group (DNSG) of the European Association for the Study of Diabetes (EASD), is on the Clinical Practice Guidelines Expert Committee for Nutrition Therapy of the EASD and is a Director of the Toronto 3D Knowledge Synthesis and Clinical Trials foundation. HK is the Director of Clinical Research of the Physicians Committee for Responsible Medicine, a non-profit organization conducting research and education. DR has served as principal investigator or co-investigator in clinical trials of AstraZeneca, Eli Lilly, Merck Sharp & Dohme (MSD), Novo Nordisk, Sanofi Aventis, Solvay, and Trophos. He has received honoraria for speaking or advisory board engagements and consulting fees from Abbott, Amgen, AstraZeneca, Bayer, Boehringer Ingelheim, Eli Lilly, Lifescan—Johnson & Johnson, MSD, Novartis, Novo Nordisk, Pfizer, Pliva, Roche, Salvus, Sanofi Aventis, and Takeda. JS-S reports serving on the board of and receiving grant support through his institution from the International Nut and Dried Fruit Council (INC) and the Eroski Foundation. He reports serving in the Executive Committee of the Instituto Danone Spain. He reports receiving research support from the Instituto de Salud Carlos III, Spain; Ministerio de Educación y Ciencia, Spain; Departament de Salut Pública de la Generalitat de Catalunya, Catalonia, Spain; European Commission; California Walnut Commission, Sacramento, CA, USA; Patrimonio Comunal Olivarero, Spain; La Morella Nuts, Spain; and Borges SA, Spain. He reports receiving consulting fees or travel expenses from Danone, California Walnut Commission, Eroski Foundation, Instituto Danone—Spain, Nuts for Life, Australian Nut Industry Council, Nestlé, Abbot Laboratories, and Font Vella Lanjarón. He is on the Clinical Practice Guidelines Expert Committee of the EASD, and served in the Scientific Committee of the Spanish Food and Safety Agency, and the Spanish Federation of the Scientific Societies of Food, Nutrition and Dietetics. He is a member of the International Carbohydrate Quality Consortium (ICQC) and an Executive Board Member of the DNSG of the EASD. JLS has received research support from the Canadian Foundation for Innovation, Ontario Research Fund, Province of Ontario Ministry of Research and Innovation and Science, Canadian Institutes of Health Research (CIHR), Diabetes Canada, PSI Foundation, Banting and Best Diabetes Centre (BBDC), American Society for Nutrition (ASN), INC International Nut and Dried Fruit Council Foundation, National Dried Fruit Trade Association, The Tate and Lyle Nutritional Research Fund at the University of Toronto, The Glycemic Control and Cardiovascular Disease in Type 2 Diabetes Fund at the University of Toronto (a fund established by the Alberta Pulse Growers), and the Nutrition Trialists Fund at the University of Toronto (a fund established by an inaugural donation from the Calorie Control Council). He has received in-kind food donations to support a randomized controlled trial from the Almond Board of California, California Walnut Commission, American Peanut Council, Barilla, Unilever, Unico/Primo, Loblaw Companies, Quaker, Kellogg Canada, and WhiteWave Foods. He has received travel support, speaker fees and/or honoraria from Diabetes Canada, Mott’s LLP, Dairy Farmers of Canada, FoodMinds LLC, International Sweeteners Association, Nestlé, Pulse Canada, Canadian Society for Endocrinology and Metabolism (CSEM), GI Foundation, Abbott, Biofortis, ASN, Northern Ontario School of Medicine, INC Nutrition Research & Education Foundation, European Food Safety Authority (EFSA), Comité Européen des Fabricants de Sucre (CEFS), and Physicians Committee for Responsible Medicine. He has or has had ad hoc consulting arrangements with Perkins Coie LLP, Tate & Lyle, and Wirtschaftliche Vereinigung Zucker e.V. He is a member of the European Fruit Juice Association Scientific Expert Panel. He is on the Clinical Practice Guidelines Expert Committees of Diabetes Canada, European Association for the study of Diabetes (EASD), Canadian Cardiovascular Society (CCS), and Obesity Canada. He serves or has served as an unpaid scientific advisor for the Food, Nutrition, and Safety Program (FNSP) and the Technical Committee on Carbohydrates of the International Life Science Institute (ILSI) North America. He is a member of the International Carbohydrate Quality Consortium (ICQC), Executive Board Member of the Diabetes and Nutrition Study Group (DNSG) of the EASD, and Director of the Toronto 3D Knowledge Synthesis and Clinical Trials foundation. His wife is an employee of Sobeys Inc. All other authors report no conflicts of interest.

With the exception of the DNSG of the EASD guidelines committee, none of the sponsors had a role in any aspect of the present study, including design and conduct of the study; collection, management, analysis, and interpretation of the data; preparation, review, or approval of the manuscript; or the decision to publish. JLS had full access to all the data in the study and had final responsibility for the decision to submit for publication. There are no patents, products in development, or marketed products to declare.

Supplemental Tables 1–28 and Supplemental Figures 1–19 are available from the “Supplementary data” link in the online posting of the article and from the same link in the online table of contents at https://academic.oup.com/advances/.

This article appears as part of the supplement “7th International Congress on Vegetarian Nutrition,” sponsored by The Harding-Buller Foundation, Worthington, OH. Publication costs for this supplement were defrayed in part by the payment of page charges. The guest editors of the supplement, Sujatha Rajaram and Joan Sabate, have no conflicts of interest. The opinions expressed in this publication are those of the authors and are not attributable to the sponsors or the publisher, Editor, or Editorial Board of *Advances in Nutrition*.

Abbreviations used: CHD, coronary heart disease; CVD, cardiovascular disease; EASD, European Association for the Study of Diabetes; GI, glycemic index; GRADE, Grading of Recommendations Assessment, Development and Evaluation; MI, myocardial infarction; NOS, Newcastle-Ottawa Scale; RR, risk ratio; SRMA, systematic review and meta-analysis.
