# Supplementary material for: Associations between Dietary Pulses Alone or with Other Legumes and Cardiometabolic Disease Outcomes: An Umbrella Review and Updated Systematic Review and Meta-analysis of Prospective Cohort Studies
Source: Adv Nutr. 2019 Nov 15;10(Suppl 4):S308–19. doi: 10.1093/advances/nmz113 (PMC6855952; doi:10.1093/advances/nmz113)
Supplement: nmz113_Supplemental_File [file nmz113_supplemental_file.docx]

**ASSOCIATIONS BETWEEN DIETARY PULSES ALONE OR WITH OTHER LEGUMES AND CARDIOMETABOLIC DISEASE OUTCOMES: AN UMBRELLA REVIEW AND UPDATED SYSTEMATIC REVIEW AND META-ANALYSIS OF PROSPECTIVE COHORT STUDIES**

[**SUPPLEMENTAL FIGURES** 4](#_Toc20754811)

[Supplemental Figure 1 – Summary of evidence search and selection for the umbrella review 4](#_Toc20754812)

[Supplemental Figure 2 – Summary of evidence search and selection for cardiovascular disease outcomes (update of Bechthold et al. 2019 (1), Grosso et al. 2017 (2) and Marvento et al. 2017 (3))* 5](#_Toc20754813)

[Supplemental Figure 3 – Summary of evidence search and selection for diabetes outcomes (update of Schwingshackl et al. 2017 (4)) 6](#_Toc20754814)

[Supplemental Figure 4 – Summary of evidence search and selection for hypertension outcomes (update of Schwingshackl et al. 2017 (5)) 7](#_Toc20754815)

[Supplemental Figure 5 – Summary of evidence search and selection for obesity outcomes (update of Schlesinger et al. 2019 (6)) 8](#_Toc20754816)

[Supplemental Figure 6 – Association between dietary pulses with or without other legumes and CVD incidence (highest vs. lowest level of intake) 9](#_Toc20754817)

[Supplemental Figure 7 – Association between dietary pulses with or without other legumes and CVD mortality (highest vs. lowest level of intake) 10](#_Toc20754818)

[Supplemental Figure 8 – Association between dietary pulses with or without other legumes and CHD incidence (highest vs. lowest level of intake) 11](#_Toc20754819)

[Supplemental Figure 9 – Association between dietary pulses with or without other legumes and CHD mortality (highest vs. lowest level of intake) 12](#_Toc20754820)

[Supplemental Figure 10 – Association between dietary pulses with or without other legumes and MI incidence (highest vs. lowest level of intake) 13](#_Toc20754821)

[Supplemental Figure 11 – Association between dietary pulses with or without other legumes and stroke incidence (highest vs. lowest level of intake) 14](#_Toc20754822)

[Supplemental Figure 12 – Association between dietary pulses with or without other legumes and stroke mortality (highest vs. lowest level of intake) 15](#_Toc20754823)

[Supplemental Figure 13 – Association between dietary pulses with or without other legumes and diabetes incidence (highest vs. lowest level of intake) 16](#_Toc20754824)

[Supplemental Figure 14 – Association between dietary pulses with or without other legumes and hypertension incidence 17](#_Toc20754825)

[Supplemental Figure 15 – Subgroup analyses for the association between dietary pulses with or without other legumes and CVD mortality 18](#_Toc20754826)

[Supplemental Figure 16 – Subgroup analyses for the association between dietary pulses with or without other legumes and CHD incidence 19](#_Toc20754827)

[Supplemental Figure 17a-h – Dose response relationship between dietary pulses with or without other legumes and cardiometabolic disease outcomes 20](#_Toc20754828)

[Supplemental Figure 18 – Publication bias analyses for the association between dietary pulses with or without other legumes and CVD mortality 22](#_Toc20754829)

[Supplemental Figure 19 – Publication bias analyses for the association between dietary pulses with or without other legumes and CHD incidence 23](#_Toc20754830)

[**SUPPLEMENTAL TABLES** 24](#_Toc20754831)

[Supplemental Table 1 – Search strategy for cardiovascular disease outcomes 24](#_Toc20754832)

[Supplemental Table 2 – Search strategy for diabetes outcomes 25](#_Toc20754833)

[Supplemental Table 3 – Search strategy for hypertension outcomes 26](#_Toc20754834)

[Supplemental Table 4 – Search strategy for obesity outcomes 27](#_Toc20754835)

[Supplemental Table 5 – Characteristics of prospective cohort studies assessing the association between dietary pulses with or without other legumes and CVD incidence (7 cohort comparisons, N=231 353) 29](#_Toc20754836)

[Supplemental Table 6 – Characteristics of prospective cohort studies assessing the association between dietary pulses with or without other legumes and CVD mortality (12 cohort comparisons, N=940 756) 30](#_Toc20754837)

[Supplemental Table 7 – Characteristics of prospective cohort studies assessing the association between dietary pulses with or without other legumes and CHD incidence (10 cohort comparisons, N=306 814) 32](#_Toc20754838)

[Supplemental Table 8 – Characteristics of prospective cohort studies assessing the association between dietary pulses with or without other legumes and CHD mortality (9 cohort comparisons, N=224 592) 34](#_Toc20754839)

[Supplemental Table 9 – Characteristics of prospective cohort studies assessing the association between dietary pulses with or without other legumes and MI incidence (4 cohort comparisons, N=202 528) 36](#_Toc20754840)

[Supplemental Table 10 – Characteristics of prospective cohort studies assessing the association between dietary pulses with or without other legumes and stroke incidence (8 cohort comparisons, N=342 079) 37](#_Toc20754841)

[Supplemental Table 11 – Characteristics of prospective cohort studies assessing the association between dietary pulses with or without other legumes and stroke mortality (6 cohort comparisons, N=168 504) 38](#_Toc20754842)

[Supplemental Table 12 – Characteristics of prospective cohort studies assessing the association between dietary pulses with or without other legumes and diabetes incidence (9 cohort comparisons, N=259 325) 39](#_Toc20754843)

[Supplemental Table 13 – Characteristics of prospective cohort studies assessing the association between dietary pulses with or without other legumes and hypertension incidence (7 cohort comparisons, N=288 352) 40](#_Toc20754844)

[Supplemental Table 14 – Characteristics of prospective cohort studies assessing the association between dietary pulses with or without other legumes and obesity incidence (1 cohort comparison, N=18 146) 41](#_Toc20754845)

[Supplemental Table 15 – Analysis of confounding variables among prospective cohort studies assessing the association between dietary pulses with or without other legumes and CVD incidence 42](#_Toc20754846)

[Supplemental Table 16 – Analysis of confounding variables among prospective cohort studies assessing the association between dietary pulses with or without other legumes and CVD mortality 44](#_Toc20754847)

[Supplemental Table 17 – Analysis of confounding variables among prospective cohort studies assessing the association between dietary pulses with or without other legumes and CHD incidence 46](#_Toc20754848)

[Supplemental Table 18 – Analysis of confounding variables among prospective cohort studies assessing the association between dietary pulses with or without other legumes and CHD mortality 48](#_Toc20754849)

[Supplemental Table 19 – Analysis of confounding variables among prospective cohort studies assessing the association between dietary pulses with or without other legumes and MI incidence 50](#_Toc20754850)

[Supplemental Table 20 – Analysis of confounding variables among prospective cohort studies assessing the association between dietary pulses with or without other legumes and stroke incidence 52](#_Toc20754851)

[Supplemental Table 21 – Analysis of confounding variables among prospective cohort studies assessing the association between dietary pulses with or without other legumes and stroke mortality 54](#_Toc20754852)

[Supplemental Table 22 – Analysis of confounding variables among prospective cohort studies assessing the association between dietary pulses with or without other legumes and diabetes incidence 56](#_Toc20754853)

[Supplemental Table 23 – Analysis of confounding variables among prospective cohort studies assessing the association between dietary pulses with or without other legumes and hypertension incidence 58](#_Toc20754854)

[Supplemental Table 24 – Analysis of confounding variables among prospective cohort studies assessing the association between dietary pulses with or without other legumes and obesity incidence 60](#_Toc20754855)

[Supplemental Table 25 – Newcastle-Ottawa Scale (NOS) for prospective cohort studies 62](#_Toc20754856)

[Supplemental Table 26 – Select sensitivity analyses in which the systematic removal of an individual study altered the significance of the pooled effect estimate or the evidence for heterogeneity 64](#_Toc20754857)

[Supplemental Table 27 – Post-hoc sensitivity analyses assessing the association in studies reporting dietary pulses alone as the exposure* 65](#_Toc20754858)

[Supplemental Table 28 – GRADE certainty of evidence assessment for the association between dietary pulses with or without other legumes and cardiometabolic disease outcomes 66](#_Toc20754859)

[**SUPPLEMENTAL REFERENCES** 68](#_Toc20754860)

# SUPPLEMENTAL FIGURES

## Supplemental Figure 1 – Summary of evidence search and selection for the umbrella review

## Supplemental Figure 2 – Summary of evidence search and selection for cardiovascular disease outcomes (update of Bechthold et al. 2019 (1), Grosso et al. 2017 (2) and Marvento et al. 2017 (3))*

*The systematic review and meta-analysis with the earliest census date was used for the updated search.

## Supplemental Figure 3 – Summary of evidence search and selection for diabetes outcomes (update of Schwingshackl et al. 2017 (4))

## Supplemental Figure 4 – Summary of evidence search and selection for hypertension outcomes (update of Schwingshackl et al. 2017 (5))

## Supplemental Figure 5 – Summary of evidence search and selection for obesity outcomes (update of Schlesinger et al. 2019 (6))

## Supplemental Figure 6 – Association between dietary pulses with or without other legumes and CVD incidence (highest vs. lowest level of intake)

Pooled risk estimate is represented by the diamond. Data are expressed as weighted risk ratios with 95% CIs using the generic inverse-variance method modelled by random effects. Values of *I^2^*≥50% and *P*<0.10 indicate substantial heterogeneity (7, 8). Values greater than 1.0 indicate an adverse association. CVD=cardiovascular disease; CI=confidence interval; EPIC=European Prospective Investigation into Cancer and Nutrition; ICS=Isfahan Cohort Study; JPHC=Japan Public Health Center; M=men; NHANES 1 NHEFS=First National Health and Nutrition Examination Survey Epidemiologic Follow-up Study; PURE=Prospective Urban Rural Epidemiology; SUN=Seguimiento Universidad de Navarra; W=women

## Supplemental Figure 7 – Association between dietary pulses with or without other legumes and CVD mortality (highest vs. lowest level of intake)

Pooled risk estimate is represented by the diamond. Data are expressed as weighted risk ratios with 95% CIs using the generic inverse-variance method modelled by random effects. Values of *I^2^*≥50% and *P*<0.10 indicate substantial heterogeneity (7, 8). Values greater than 1.0 indicate an adverse association. CVD=cardiovascular disease; CI=confidence interval; EPIC=European Prospective Investigation into Cancer and Nutrition; HAPIEE=Health Alcohol and Psychosocial factors in Eastern Europe Study; JACC=Japan Collaborative Cohort; JPHC=Japan Public Health Center; M=men; MCCS=Melbourne Collaborative Cohort Study; NOMAS=Northern Manhattan Study; PREDIMED=PREvencion con DIeta MEDiterranea study; PURE=Prospective Urban Rural Epidemiology; W=women

## Supplemental Figure 8 – Association between dietary pulses with or without other legumes and CHD incidence (highest vs. lowest level of intake)

Pooled risk estimate is represented by the diamond. Data are expressed as weighted risk ratios with 95% CIs using the generic inverse-variance method modelled by random effects. Values of *I^2^*≥50% and *P*<0.10 indicate substantial heterogeneity (7, 8). Values greater than 1.0 indicate an adverse association. ARIC=Atherosclerosis Risk in Communities; CHD=coronary heart disease; CI=confidence interval; EPIC=European Prospective Investigation into Cancer and Nutrition; M=men; NHANES 1 NHEFS=First National Health and Nutrition Examination Survey Epidemiologic Follow-up Study; NHS=Nurses’ Health Study; SMHS=Shanghai Men's Health Study; SUN=Seguimiento Universidad de Navarra; SWHS=Shanghai Women’s Health Study; W=wome

## Supplemental Figure 9 – Association between dietary pulses with or without other legumes and CHD mortality (highest vs. lowest level of intake)

Pooled risk estimate is represented by the diamond. Data are expressed as weighted risk ratios with 95% CIs using the generic (9-11) inverse-variance method modelled by random effects. Values of *I^2^*≥50% and *P*<0.10 indicate substantial heterogeneity (7, 8). Values greater than 1.0 indicate an adverse association. AHS=Adventists Health Study; CHD=coronary heart disease; CI=confidence interval; EPIC=European Prospective Investigation into Cancer and Nutrition; HAPIEE=Health Alcohol and Psychosocial factors in Eastern Europe Study; IWHS=Iowa Women's Health Study; JACC=Japan Collaborative Cohort; M=men; NIT=Nutrition Intervention Trials; W=women

## Supplemental Figure 10 – Association between dietary pulses with or without other legumes and MI incidence (highest vs. lowest level of intake)

Pooled risk estimate is represented by the diamond. Data are expressed as weighted risk ratios with 95% CIs using the generic inverse-variance method modelled by fixed effects. Values of *I^2^*≥50% and *P*<0.10 indicate substantial heterogeneity (7, 8). Values greater than 1.0 indicate an adverse association. AHS=Adventists Health Study; CI=confidence interval; JPHC=Japan Public Health Center; M=men; MI=myocardial infarction; PURE=Prospective Urban Rural Epidemiology; W=women

## Supplemental Figure 11 – Association between dietary pulses with or without other legumes and stroke incidence (highest vs. lowest level of intake)

Pooled risk estimate is represented by the diamond. Data are expressed as weighted risk ratios with 95% CIs using the generic inverse-variance method modelled by random effects. Values of *I^2^*≥50% and *P*<0.10 indicate substantial heterogeneity (7, 8). Values greater than 1.0 indicate an adverse association. ARIC=Atherosclerosis Risk in Communities; CI=confidence interval; EPIC=European Prospective Investigation into Cancer and Nutrition; HES=Health Examination Survey; HPFS=Health Professionals Follow-Up Study; JPHC=Japan Public Health Center; M=men; NHS=Nurses’ Health Study; PURE=Prospective Urban Rural Epidemiology; W=women

## Supplemental Figure 12 – Association between dietary pulses with or without other legumes and stroke mortality (highest vs. lowest level of intake)

Pooled risk estimate is represented by the diamond. Data are expressed as weighted risk ratios with 95% CIs using the generic inverse-variance method modelled by random effects. Values of *I^2^*≥50% and *P*<0.10 indicate substantial heterogeneity (7, 8). Values greater than 1.0 indicate an adverse association. CI=confidence interval; EPIC=European Prospective Investigation into Cancer and Nutrition; HAPIEE=Health Alcohol and Psychosocial factors in Eastern Europe Study; JACC=Japan Collaborative Cohort; NIT=Nutrition Intervention Trials

## Supplemental Figure 13 – Association between dietary pulses with or without other legumes and diabetes incidence (highest vs. lowest level of intake)

Pooled risk estimate is represented by the diamond. Data are expressed as weighted risk ratios with 95% CIs using the generic inverse-variance method modelled by random effects. Values of *I^2^*≥50% and *P*<0.10 indicate substantial heterogeneity (7, 8). Values greater than 1.0 indicate an adverse association. CI=confidence interval; EPIC=European Prospective Investigation into Cancer and Nutrition; IWHS=Iowa Women's Health Study; M=men; MCCS=Melbourne Collaborative Cohort Study; MDC=Malmo Diet and Cancer; NHS=Nurses’ Health Study; PREDIMED=PREvencion con DIeta MEDiterranea study; SWHS=Shanghai Women's Health Study; TLGS=Tehran Lipid and Glucose Study; W=women

## Supplemental Figure 14 – Association between dietary pulses with or without other legumes and hypertension incidence

Pooled risk estimate is represented by the diamond. Data are expressed as weighted risk ratios with 95% CIs using the generic inverse-variance method modelled by random effects. Values of *I^2^*≥50% and *P*<0.10 indicate substantial heterogeneity (7, 8). Values greater than 1.0 indicate an adverse association. ARIC=Atherosclerosis Risk in Communities; HPFS=Health Professionals Follow-Up Study; NHS=Nurses’ Health Study; SUN=Seguimiento Universidad de Navarra; TLGS=Tehran Lipid and Glucose Study

## Supplemental Figure 15 – Subgroup analyses for the association between dietary pulses with or without other legumes and CVD mortality

NOS=Newcastle-Ottawa Scale. Point estimates for each subgroup level (diamonds) are the pooled effect estimates. The dashed line represents the pooled effect estimate for the overall (total) analysis. The residual *I^2^* value represents unexplained heterogeneity for each subgroup. For the “Exposure” subgroup, studies included under the “Pulses” level included those reporting only chickpeas, lentils, beans, and/or peas in the exposure, whereas studies included under “Pulses + other legumes” included those reporting “legumes” without differentiating the legume type or included other types of legumes in the exposure in addition to pulses (i.e. soybeans, soy products, peanuts, fresh peas and/or fresh beans). (Note: if beans and peas were included in the exposure and did not specify whether they were fresh and/or dry, the study was categorized under “Pulses”). *Source of funding for two studies were unknown (12, 13)

## Supplemental Figure 16 – Subgroup analyses for the association between dietary pulses with or without other legumes and CHD incidence

NOS=Newcastle-Ottawa Scale. Point estimates for each subgroup level (diamonds) are the pooled effect estimates. The dashed line represents the pooled effect estimate for the overall (total) analysis. The residual *I^2^* value represents unexplained heterogeneity for each subgroup. For the “Exposure” subgroup, studies included under the “Pulses” level included those reporting only chickpeas, lentils, beans, and/or peas in the exposure, whereas studies included under “Pulses + other legumes” included those reporting “legumes” without differentiating the legume type or included other types of legumes in the exposure in addition to pulses (i.e. soybeans, soy products, peanuts, fresh peas and/or fresh beans). (Note: if beans and peas were included in the exposure and did not specify whether they were fresh and/or dry, the study was categorized under “Pulses”).

## Supplemental Figure 17a-h – Dose response relationship between dietary pulses with or without other legumes and cardiometabolic disease outcomes

**A – CVD Incidence**

Black solid and grey dashed lines represent the linear and non-linear spline models respectively along with their confidence intervals. The light grey circles represent the relative risk point estimates for the different doses from each study; the size of the circle is related to inverse of the variance. Three cohort comparisons were available for dose-response analyses for CVD incidence.

**Supplemental Figure 17a-h *(continued)***

**B – CVD Mortality**

Black solid and grey dashed lines represent the linear and non-linear spline models respectively along with their confidence intervals. The light grey circles represent the relative risk point estimates for the different doses from each study; the size of the circle is related to inverse of the variance. Seven cohort comparisons were available for dose-response analyses for CVD mortality.

**Supplemental Figure 17a-h *(continued)***

**C – CHD Incidence**

Black solid and grey dashed lines represent the linear and non-linear spline models respectively along with their confidence intervals. The light grey circles represent the relative risk point estimates for the different doses from each study; the size of the circle is related to inverse of the variance. Seven cohort comparisons were available for dose-response analyses for CHD incidence.

**Supplemental Figure 17a-h *(continued)***

**D – CHD Mortality**

Black solid and grey dashed lines represent the linear and non-linear spline models respectively along with their confidence intervals. The light grey circles represent the relative risk point estimates for the different doses from each study; the size of the circle is related to inverse of the variance. Four cohort comparisons were available for dose-response analyses for CHD mortality.

**Supplemental Figure 17a-h *(continued)***

**E – Stroke Incidence**

Black solid and grey dashed lines represent the linear and non-linear spline models respectively along with their confidence intervals. The light grey circles represent the relative risk point estimates for the different doses from each study; the size of the circle is related to inverse of the variance. Five cohort comparisons were available for dose-response analyses for stroke incidence.

**Supplemental Figure 17a-h *(continued)***

**F – Stroke Mortality**

Black solid and grey dashed lines represent the linear and non-linear spline models respectively along with their confidence intervals. The light grey circles represent the relative risk point estimates for the different doses from each study; the size of the circle is related to inverse of the variance. Three cohort comparisons were available for dose-response analyses for stroke mortality.

**Supplemental Figure 17a-h *(continued)***

**G – Diabetes Incidence**

Black solid and grey dashed lines represent the linear and non-linear spline models respectively along with their confidence intervals. The light grey circles represent the relative risk point estimates for the different doses from each study; the size of the circle is related to inverse of the variance. Seven cohort comparisons were available for dose-response analyses for diabetes incidence.

**Supplemental Figure 17a-h *(continued)***

**H – Hypertension Incidence**

Black solid and grey dashed lines represent the linear and non-linear spline models respectively along with their confidence intervals. The light grey circles represent the relative risk point estimates for the different doses from each study; the size of the circle is related to inverse of the variance. Six cohort comparisons were available for dose-response analyses for hypertension incidence.

## Supplemental Figure 18 – Publication bias analyses for the association between dietary pulses with or without other legumes and CVD mortality

The vertical line represents the pooled effect estimate expressed as a RR. Dashed lines represent pseudo-95% confidence intervals (CI). The circles represent risk estimates for each cohort, and the horizontal lines represent standard errors of the RR.

## Supplemental Figure 19 – Publication bias analyses for the association between dietary pulses with or without other legumes and CHD incidence

The vertical line represents the pooled effect estimate expressed as a RR. Dashed lines represent pseudo-95% confidence intervals (CI). The circles represent risk estimates for each cohort, and the horizontal lines represent standard errors of the RR.

# SUPPLEMENTAL TABLES

## Supplemental Table 1 – Search strategy for cardiovascular disease outcomes

| **MEDLINE** | |  | **EMBASE** | |  | **COCHRANE** | |
| --- | --- | --- | --- | --- | --- | --- | --- |
| 1 | pulses.mp |  | 1 | pulses.mp |  | 1 | pulses.mp |
| 2 | fabaceae.mp |  | 2 | fabaceae.mp |  | 2 | fabaceae.mp |
| 3 | bean*.mp |  | 3 | bean*.mp |  | 3 | bean*.mp |
| 4 | pea.mp |  | 4 | pea.mp |  | 4 | pea.mp |
| 5 | peas.mp |  | 5 | peas.mp |  | 5 | peas.mp |
| 6 | chickpea*.mp |  | 6 | chickpea*.mp |  | 6 | chickpea*.mp |
| 7 | lentil*.mp |  | 7 | lentil*.mp |  | 7 | lentil*.mp |
| 8 | legume*.mp |  | 8 | legume*.mp |  | 8 | legume*.mp |
| 9 | leguminous.mp |  | 9 | leguminous.mp |  | 9 | leguminous.mp |
| 10 | or/1-9 |  | 10 | or/1-9 |  | 10 | or/1-9 |
|  |  |  |  |  |  |  |  |
| 11 | cohort.mp. |  | 11 | cohort.mp. |  | 11 | cohort.mp. |
| 12 | exp prospective study/ |  | 12 | exp prospective study/ |  | 12 | exp Prospective Studies/ |
| 13 | (prospective adj2 (cohort or study)).mp. |  | 13 | (prospective adj2 (cohort or study)).mp. |  | 13 | (prospective adj2 (cohort or study)).mp. |
| 14 | exp follow-up studies/ |  | 14 | exp multivariate analysis/ |  | 14 | exp follow-up studies/ |
| 15 | exp multivariate analysis/ |  | 15 | exp proportional hazards models/ |  | 15 | exp multivariate analysis/ |
| 16 | exp proportional hazards models/ |  | 16 | follow up study.mp. |  | 16 | exp proportional hazards models/ |
| 17 | follow up study.mp. |  | 17 | (longitudinal adj2 study).mp. |  | 17 | follow up study.mp. |
| 18 | (longitudinal adj2 study).mp. |  | 18 | or/11-17 |  | 18 | (longitudinal adj2 study).mp. |
| 19 | or/11-18 |  |  |  |  | 19 | or/11-18 |
|  |  |  |  |  |  |  |  |
| 20 | cardiovascular disease.mp. |  | 19 | cardiovascular disease.mp. |  | 20 | cardiovascular disease.mp. |
| 21 | cvd.mp. |  | 20 | cvd.mp. |  | 21 | cvd.mp. |
| 22 | (coronary adj2 disease).mp. |  | 21 | (coronary adj2 disease).mp. |  | 22 | (coronary adj2 disease).mp. |
| 23 | exp coronary disease/ |  | 22 | exp coronary disease/ |  | 23 | exp coronary disease/ |
| 24 | cerebrovascular.mp. |  | 23 | cerebrovascular.mp. |  | 24 | cerebrovascular.mp. |
| 25 | cerebral vascular.mp. |  | 24 | cerebral vascular.mp. |  | 25 | cerebral vascular.mp. |
| 26 | exp brain ischemia/ |  | 25 | exp brain ischemia/ |  | 26 | exp brain ischemia/ |
| 27 | exp stroke/ |  | 26 | exp stroke/ |  | 27 | exp stroke/ |
| 28 | exp cerebrovascular disorders/ |  | 27 | exp cerebrovascular disorders/ |  | 28 | exp cerebrovascular disorders/ |
| 29 | exp intracranial arterial diseases/ |  | 28 | exp intracranial arterial diseases/ |  | 29 | exp intracranial arterial diseases/ |
| 30 | exp myocardial infarction/ |  | 29 | exp myocardial infarction/ |  | 30 | exp myocardial infarction/ |
| 31 | myocardial infarction.mp. |  | 30 | myocardial infarction.mp. |  | 31 | myocardial infarction.mp. |
| 32 | exp myocardial ischemia/ |  | 31 | exp myocardial ischemia/ |  | 32 | exp myocardial ischemia/ |
| 33 | myocardial ischemia.mp. |  | 32 | myocardial ischemia.mp. |  | 33 | myocardial ischemia.mp. |
| 34 | or/20-33 |  | 33 | or/19-32 |  | 34 | or/20-33 |
|  |  |  |  |  |  |  |  |
| 35 | 10 and 19 and 34 |  | 34 | 10 and 18 and 33 |  | 35 | 10 and 19 and 34 |

## Supplemental Table 2 – Search strategy for diabetes outcomes

| **MEDLINE** | |  | **EMBASE** | |  | **COCHRANE** | |
| --- | --- | --- | --- | --- | --- | --- | --- |
| 1 | pulses.mp. |  | 1 | pulses.mp. |  | 1 | pulses.mp. |
| 2 | fabaceae.mp. |  | 2 | fabaceae.mp. |  | 2 | fabaceae.mp. |
| 3 | bean*.mp. |  | 3 | bean*.mp. |  | 3 | bean*.mp. |
| 4 | pea.mp. |  | 4 | pea.mp. |  | 4 | pea.mp. |
| 5 | peas.mp. |  | 5 | peas.mp. |  | 5 | peas.mp. |
| 6 | chickpea*.mp. |  | 6 | chickpea*.mp. |  | 6 | chickpea*.mp. |
| 7 | lentil*.mp. |  | 7 | lentil*.mp. |  | 7 | lentil*.mp. |
| 8 | legume*.mp. |  | 8 | legume*.mp. |  | 8 | legume*.mp. |
| 9 | leguminous.mp. |  | 9 | leguminous.mp. |  | 9 | leguminous.mp. |
| 10 | or/1-9 |  | 10 | or/1-9 |  | 10 | or/1-9 |
|  |  |  |  |  |  |  |  |
| 11 | cohort.mp. |  | 11 | cohort.mp. |  | 11 | cohort.mp. |
| 12 | exp prospective study/ |  | 12 | exp prospective study/ |  | 12 | exp Prospective Studies/ |
| 13 | (prospective adj2 (cohort or study)).mp. |  | 13 | (prospective adj2 (cohort or study)).mp. |  | 13 | (prospective adj2 (cohort or study)).mp. |
| 14 | exp follow-up studies/ |  | 14 | exp follow-up studies/ |  | 14 | exp follow-up studies/ |
| 15 | exp multivariate analysis/ |  | 15 | exp multivariate analysis/ |  | 15 | exp multivariate analysis/ |
| 16 | exp proportional hazards models/ |  | 16 | exp proportional hazards models/ |  | 16 | exp proportional hazards models/ |
| 17 | follow up study.mp. |  | 17 | follow up study.mp. |  | 17 | follow up study.mp. |
| 18 | (longitudinal adj2 study).mp. |  | 18 | (longitudinal adj2 study).mp. |  | 18 | (longitudinal adj2 study).mp. |
| 19 | or/11-18 |  | 19 | or/11-18 |  | 19 | or/11-18 |
|  |  |  |  |  |  |  |  |
| 20 | exp Diabetes Mellitus/ |  | 19 | exp diabetes mellitus/ |  | 20 | Diabetes Mellitus/ |
| 21 | "Type 2 Diabetes*".mp. |  | 20 | "Type 2 Diabetes".mp. |  | 21 | Diabetes Mellitus, Type 2/ |
| 22 | 20 or 21 |  | 21 | 22. 20 or 21 |  | 22 | "Type 2 Diabetes".mp. |
|  |  |  |  |  |  | 23 | 20 or 21 or 22 |
|  |  |  |  |  |  |  |  |
| 23 | 10 and 19 and 22 |  | 22 | 10 and 19 and 22 |  | 24 | 10 and 19 and 23 |

## Supplemental Table 3 – Search strategy for hypertension outcomes

| **MEDLINE** | |  | **EMBASE** | |  | **COCHRANE** | |
| --- | --- | --- | --- | --- | --- | --- | --- |
| 1 | pulses.mp |  | 1 | pulses.mp |  | 1 | pulses.mp |
| 2 | fabaceae.mp |  | 2 | fabaceae.mp |  | 2 | fabaceae.mp |
| 3 | bean*.mp |  | 3 | bean*.mp |  | 3 | bean*.mp |
| 4 | pea.mp |  | 4 | pea.mp |  | 4 | pea.mp |
| 5 | peas.mp |  | 5 | peas.mp |  | 5 | peas.mp |
| 6 | chickpea*.mp |  | 6 | chickpea*.mp |  | 6 | chickpea*.mp |
| 7 | lentil*.mp |  | 7 | lentil*.mp |  | 7 | lentil*.mp |
| 8 | legume*.mp |  | 8 | legume*.mp |  | 8 | legume*.mp |
| 9 | leguminous.mp |  | 9 | leguminous.mp |  | 9 | leguminous.mp |
| 10 | or/1-9 |  | 10 | or/1-9 |  | 10 | or/1-9 |
|  |  |  |  |  |  |  |  |
| 11 | cohort.mp. |  | 11 | cohort.mp. |  | 11 | cohort.mp. |
| 12 | exp prospective study/ |  | 12 | exp prospective study/ |  | 12 | exp Prospective Studies/ |
| 13 | (prospective adj2 (cohort or study)).mp. |  | 13 | (prospective adj2 (cohort or study)).mp. |  | 13 | (prospective adj2 (cohort or study)).mp. |
| 14 | exp follow-up studies/ |  | 14 | exp multivariate analysis/ |  | 14 | exp follow-up studies/ |
| 15 | exp multivariate analysis/ |  | 15 | exp proportional hazards models/ |  | 15 | exp multivariate analysis/ |
| 16 | exp proportional hazards models/ |  | 16 | follow up study.mp. |  | 16 | exp proportional hazards models/ |
| 17 | follow up study.mp. |  | 17 | (longitudinal adj2 study).mp. |  | 17 | follow up study.mp. |
| 18 | (longitudinal adj2 study).mp. |  | 18 | or/11-17 |  | 18 | (longitudinal adj2 study).mp. |
| 19 | or/11-18 |  |  |  |  | 19 | or/11-18 |
|  |  |  |  |  |  |  |  |
| 20 | hypertensive*.mp. |  | 19 | hypertensive*.mp. |  | 20 | hypertensive*.mp. |
| 21 | exp Hypertension/ |  | 20 | exp Hypertension/ |  | 21 | exp Hypertension/ |
| 22 | hypertension*.mp. |  | 21 | hypertension*.mp. |  | 22 | hypertension*.mp. |
| 23 | HTN.mp. |  | 22 | HTN.mp. |  | 23 | HTN.mp. |
| 24 | blood pressure.mp. |  | 23 | blood pressure.mp. |  | 24 | blood pressure.mp. |
| 25 | exp Blood Pressure/ |  | 24 | exp Blood Pressure/ |  | 25 | exp Blood Pressure/ |
| 26 | systolic blood pressure.mp. |  | 25 | systolic blood pressure.mp. |  | 26 | systolic blood pressure.mp. |
| 27 | SBP.mp. |  | 26 | SBP.mp. |  | 27 | SBP.mp. |
| 28 | diastolic blood pressure.mp. |  | 27 | diastolic blood pressure.mp. |  | 28 | diastolic blood pressure.mp. |
| 29 | DBP.mp. |  | 28 | DBP.mp. |  | 29 | DBP.mp. |
| 30 | or/20-29 |  | 29 | or/19-28 |  | 30 | or/20-29 |
|  |  |  |  |  |  |  |  |
| 31 | 10 and 19 and 30 |  | 30 | 10 and 18 and 29 |  | 31 | 10 and 19 and 30 |

## Supplemental Table 4 – Search strategy for obesity outcomes

| **MEDLINE** | |  | **EMBASE** | |  | **COCHRANE** | |
| --- | --- | --- | --- | --- | --- | --- | --- |
| 1 | pulses.mp |  | 1 | pulses.mp |  | 1 | pulses.mp |
| 2 | fabaceae.mp |  | 2 | fabaceae.mp |  | 2 | fabaceae.mp |
| 3 | bean*.mp |  | 3 | bean*.mp |  | 3 | bean*.mp |
| 4 | pea.mp |  | 4 | pea.mp |  | 4 | pea.mp |
| 5 | peas.mp |  | 5 | peas.mp |  | 5 | peas.mp |
| 6 | chickpea*.mp |  | 6 | chickpea*.mp |  | 6 | chickpea*.mp |
| 7 | lentil*.mp |  | 7 | lentil*.mp |  | 7 | lentil*.mp |
| 8 | legume*.mp |  | 8 | legume*.mp |  | 8 | legume*.mp |
| 9 | leguminous.mp |  | 9 | leguminous.mp |  | 9 | leguminous.mp |
| 10 | or/1-9 |  | 10 | or/1-9 |  | 10 | or/1-9 |
|  |  |  |  |  |  |  |  |
| 11 | cohort.mp. |  | 11 | cohort.mp. |  | 11 | cohort.mp. |
| 12 | exp prospective study/ |  | 12 | exp prospective study/ |  | 12 | exp Prospective Studies/ |
| 13 | (prospective adj2 (cohort or study)).mp. |  | 13 | (prospective adj2 (cohort or study)).mp. |  | 13 | (prospective adj2 (cohort or study)).mp. |
| 14 | exp follow-up studies/ |  | 14 | exp multivariate analysis/ |  | 14 | exp follow-up studies/ |
| 15 | exp multivariate analysis/ |  | 15 | exp proportional hazards models/ |  | 15 | exp multivariate analysis/ |
| 16 | exp proportional hazards models/ |  | 16 | follow up study.mp. |  | 16 | exp proportional hazards models/ |
| 17 | follow up study.mp. |  | 17 | (longitudinal adj2 study).mp. |  | 17 | follow up study.mp. |
| 18 | (longitudinal adj2 study).mp. |  | 18 | or/11-17 |  | 18 | (longitudinal adj2 study).mp. |
| 19 | or/11-18 |  |  |  |  | 19 | or/11-18 |
|  |  |  |  |  |  |  |  |
| 20 | exp Body Weight/ |  | 19 | exp Body Weight/ |  | 20 | body weight/ |
| 21 | body weight*.mp. |  | 20 | body weight*.mp. |  | 21 | body weight*.mp. |
| 22 | exp Body Mass Index/ |  | 21 | exp Body Mass Index/ |  | 22 | exp body mass index/ |
| 23 | body mass index.mp. |  | 22 | body mass index.mp. |  | 23 | body mass index.mp. |
| 24 | BMI.mp. |  | 23 | BMI.mp. |  | 24 | BMI.mp. |
| 25 | exp Waist Circumference/ |  | 24 | exp Waist Circumference/ |  | 25 | exp waist circumference/ |
| 26 | waist circumference.mp. |  | 25 | waist circumference.mp. |  | 26 | waist circumference.mp. |
| 27 | exp Waist-Hip Ratio/ |  | 26 | exp Waist-Hip Ratio/ |  | 27 | exp waist-hip ratio/ |
| 28 | waist-hip ratio.mp. |  | 27 | waist-hip ratio.mp. |  | 28 | waist-hip ratio.mp. |
| 29 | exp Overweight/ |  | 28 | exp Overweight/ |  | 29 | exp Overweight/ |
| 30 | overweight.mp. |  | 29 | overweight.mp. |  | 30 | overweight.mp. |
| 31 | exp Obesity/ |  | 30 | exp Obesity/ |  | 31 | Obesity/ |
| 32 | exp Obesity, Abdominal/ |  | 31 | exp Obesity, Abdominal/ |  | 32 | exp Obesity, Abdominal/ |
| 33 | exp Obesity, morbid/ |  | 32 | exp Obesity, morbid/ |  | 33 | "body weights and measures"/ |
| 34 | obesity.mp. |  | 33 | obesity.mp. |  | 34 | exp body fat distribution/ |
| 35 | body fat.mp. |  | 34 | body fat.mp. |  | 35 | exp skinfold thickness/ |
| 36 | fat mass.mp. |  | 35 | fat mass.mp. |  | 36 | obesity.mp. |
| 37 | or/20-36 |  | 36 | or/18-35 |  | 37 | exp adipose tissue/ |
|  |  |  |  |  |  | 38 | body fat.mp. |
|  |  |  |  |  |  | 39 | fat mass.mp. |
|  |  |  |  |  |  | 40 | or/20-39 |
|  |  |  |  |  |  |  |  |
| 38 | 10 and 19 and 37 |  | 37 | 10 and 18 and 36 |  | 41 | 10 and 19 and 40 |

| **Study (Supplemental reference)** | **Cohort** | **Country** | **No.**  **participants** | **Age range,**  **y** | **Follow-up,**  **y** | **No.**  **cases** | **Outcome**  **assessment**  **method** | **Exposure ^1^** | **Diet assessment**  **method** | **Quantile**  **divisions** | **Lowest**  **quantile ^2^,**  **g/d** | **Highest**  **quantile ^2^,**  **g/d** | **Funding**  **source ^3^** |
| --- | --- | --- | --- | --- | --- | --- | --- | --- | --- | --- | --- | --- | --- |
| Bazzano et al. 2001 (14) | NHANES 1  NHEFS | USA | 9632 M, W | 25-74 | 19 | 3680 | Medical records | Pinto beans, red beans, black-eye peas, peanuts, peanut butter | FFQ + 24-hr  recall | Quartiles | ~5.9 | ~80.9 | Agency |
| Kokubo et al. 2007 – M (10) | JPHC | Japan | 19 466 M | 40-59 | 12.5 | 629 | Medical records | Beans | FFQ, validated | Tertiles | NR | NR | Agency |
| Kokubo et al. 2007 – W (10) | JPHC | Japan | 20 984 W | 40-59 | 12.5 | 266 | Medical records | Beans | FFQ, validated | Tertiles | NR | NR | Agency |
| von Ruesten et al. 2013 (15) | EPIC-Potsdam | Germany | 9098 M,  14 433 W | 35-65 | 8 | 363 | Medical records | Green beans,  green peas, lentil/pea/  bean stew | SFFQ, validated | per 100 g/d | NR | NR | Agency |
| Nouri et al. 2016 (16) | ICS | Iran | 2629 M,  2769 W | ≥35 | 6.8 | 427 | Medical records | Legumes | SFFQ, validated | Tertiles | NR | NR | NR |
| Buil-Cosailes et al. 2017 (17) | SUN | Spain | 6633 M,  10 374 W | 18–101 | 9.6 | 112 | Medical records | Lentils, chickpeas, beans, peas (18) | SFFQ, validated | Quintiles | 8.4 | 36 | Agency |
| Miller et al. 2017 (11) | PURE | 18  countries ^4^ | 56 422 M,  78 913 W | 35-70 | 7.4 | 4784 | Medical records | Beans,  black beans, lentils, peas, chickpeas, black-eyed peas | FFQ, validated | Quintiles | ~0 | ~213 | Agency-  Industry |

## Supplemental Table 5 – Characteristics of prospective cohort studies assessing the association between dietary pulses with or without other legumes and CVD incidence (7 cohort comparisons, N=231 353)

CVD=cardiovascular disease; EPIC=European Prospective Investigation into Cancer and Nutrition; FFQ=food frequency questionnaire; ICS=Isfahan Cohort Study; JPHC=Japan Public Health Center; M=men; NHANES 1 NHEFS=First National Health and Nutrition Examination Survey Epidemiologic Follow-up Study; NR=not reported; PURE= Prospective Urban Rural Epidemiology; SFFQ=semi-quantitative food frequency questionnaire; SUN=Seguimiento Universidad de Navarra; W=women; y=years

^1^ For studies that reported “legumes” as the exposure without differentiating the legume type, an attempt was made to access the diet assessment tool used by the study or other relevant publications in order to verify the legume type. A reference was provided for those studies where we were able to obtain more information regarding the legume type.

^2^ Lowest and highest quantiles preceded by “~” represents quantiles that have been converted to g/d from another measure (e.g. servings/d were converted to g/d using a defined serving size provided by the study or the serving size was estimated using relevant dietary guidelines).

^3^ Agency funding is that from government, university, or not-for-profit sources. Industry funding is that from trade organizations that obtain revenue from the sale of products.

^4^ Canada, Sweden, United Arab Emirates, Argentina, Brazil, Chile, Malaysia, Poland, South Africa, Turkey, China, Colombia, Iran, occupied Palestinian territory, Bangladesh, India, Pakistan, and Zimbabwe.

## Supplemental Table 6 – Characteristics of prospective cohort studies assessing the association between dietary pulses with or without other legumes and CVD mortality (12 cohort comparisons, N=940 756)

| **Study**  **(Supplemental reference)** | **Cohort** | **Country** | **No.**  **participants** | **Age range, y** | **Follow-up, y** | **No.**  **cases** | **Outcome**  **assessment**  **method** | **Exposure ^1^** | **Diet assessment method** | **Quantile divisions** | **Lowest**  **quantile ^2^,**  **g/d** | **Highest**  **quantile ^2^,**  **g/d** | **Funding**  **source ^3^** |
| --- | --- | --- | --- | --- | --- | --- | --- | --- | --- | --- | --- | --- | --- |
| Kokubo et al. 2007 – M (10) | JPHC | Japan | 19 466 M | 40-59 | 12.5 | 175 | Medical records | Beans | FFQ, validated | Tertiles | NR | NR | Agency |
| Kokubo et al. 2007 – W (10) | JPHC | Japan | 20 984 W | 40-59 | 12.5 | 57 | Medical records | Beans | FFQ, validated | Tertiles | NR | NR | Agency |
| Nagura et al. 2009 (19) | JACC | Japan | 25 206 M,  34 279 W | 40-79 | 12.7 | 2243 | Medical records | Tofu, boiled beans | FFQ, validated | Quartiles | ~5.7 | ~32.1 | Agency |
| Gardener et al. 2011 (20) | NOMAS | USA | 1637 M,  931 W | >40 | 9 | 314 | Medical records | Legumes | FFQ, validated, interviewer administered | Median | NR | NR | Agency |
| Russell et al. 2014 (12) | MCCS | Australia | 40 625 M, W | 40-69 ^4^ | 15.9 | 1365 | Medical records | Lentils, chickpeas, dry beans, dry peas, baked beans, green beans, green peas (21) | FFQ | Tertiles | NR | NR | Agency |
| Lassale et al. 2015 (22) | EPIC | 10 countries ^5^ | 130 370 M,  320 886 W | 35-70 | 12.8 | 5083 | Medical records | Legumes | Several methods, validated ^6^ | Quintiles | 5 | 25 | NR |
| Bonaccio et al. 2017 (23) | Moli-sani | Italy | 9931 M,  11 376 W | 35-98 | 8.3 | 289 | Medical records | Lentils, beans, chickpeas, fava beans, green peas | SFFQ, validated, interviewer administered | Quartiles | 8.3 | 53.8 | Agency-Industry |
| Farvid et al. 2017 (24) | Golestan Cohort Study | Iran | 18 261 M,  24 142 W | 36-85 | 8.1 | 1467 | Medical records | Soybeans, beans, lentils, peas, split peas | SFFQ, validated, interviewer administered | Quintiles | ~3 | ~26 | Agency |
| Miller et al. 2017 (11) | PURE | 18 countries ^7^ | 56 422 M,  78 913 W | 35-70 | 7.4 | 1649 | Medical records | Beans, black beans, lentils, peas, chickpeas, black-eyed peas | FFQ, validated | Quintiles | ~0 | ~213 | Agency-Industry |
| Stefler et al. 2017 (25) | HAPIEE Study | Czech Republic, Poland, Russia | 19 263 M,W | 57 | 7 | 438 | Medical records | Legumes | SFFQ, validated | per 1 point increase in component score | NR | NR | Agency |
| Papandreou et al. 2018 (26) | PREDIMED | Spain | 3068 M, 4144 W | 55-80 M  60-80 W | 6 | 103 | Medical records | Lentils, chickpeas, dry beans, fresh peas | SFFQ, validated, interviewer administered | Tertiles | 14.0 | 27.3 | Agency-Industry |
| van den Brandt et al. 2019 (13) | The Netherlands Cohort Study | Netherlands | 58 279 M,  62 573 W | 55-69 | 10 | 2985 | Medical records | Pulses | SFFQ, validated | Quartiles | 0 | 27.8 | NR |

CVD=cardiovascular disease; EPIC=European Prospective Investigation into Cancer and Nutrition; FFQ=food frequency questionnaire; HAPIEE=Health Alcohol and Psychosocial factors in Eastern Europe Study; JACC=Japan Collaborative Cohort; JPHC=Japan Public Health Center; M=men; MCCS=Melbourne Collaborative Cohort Study; NOMAS=Northern Manhattan Study; NR=not reported; PREDIMED=PREvencion con DIeta MEDiterranea study; PURE= Prospective Urban Rural Epidemiology; SFFQ=semi-quantitative food frequency questionnaire; W=women; y=years

^1^ For studies that reported “legumes” as the exposure without differentiating the legume type, an attempt was made to access the diet assessment tool used by the study or other relevant publications in order to verify the legume type. A reference was provided for those studies where we were able to obtain more information regarding the legume type.

^2^ Lowest and highest quantiles preceded by “~” represents quantiles that have been converted to g/d from another measure (e.g. servings/d were converted to g/d using a defined serving size provided by the study or the serving size was estimated using relevant dietary guidelines).

^3^ Agency funding is that from government, university, or not-for-profit sources. Industry funding is that from trade organizations that obtain revenue from the sale of products.

^4^ Participants age range at baseline was 27-75 years, where 99% of participants age ranged 40-69 years.

^5^ Norway, Sweden, Denmark, UK, Netherlands, Germany, France, Spain, Italy, Greece.

^6^ SFFQ, FFQ, interviewer administered quantitative dietary questionnaires, and/or food records.

^7^ Canada, Sweden, United Arab Emirates, Argentina, Brazil, Chile, Malaysia, Poland, South Africa, Turkey, China, Colombia, Iran, occupied Palestinian territory, Bangladesh, India, Pakistan, and Zimbabwe.

## Supplemental Table 7 – Characteristics of prospective cohort studies assessing the association between dietary pulses with or without other legumes and CHD incidence (10 cohort comparisons, N=306 814)

| **Study (Supplemental reference)** | **Cohort** | **Country** | **No.**  **participants** | **Age range,**  **y** | **Follow-up,**  **y** | **No.**  **cases** | **Outcome**  **assessment**  **method** | **Exposure ^1^** | **Diet assessment method** | **Quantile**  **divisions** | **Lowest**  **quantile ^2^,**  **g/d** | **Highest**  **quantile ^2^,**  **g/d** | **Funding**  **source ^3^** |
| --- | --- | --- | --- | --- | --- | --- | --- | --- | --- | --- | --- | --- | --- |
| Bazzano et al. 2001 (14) | NHANES 1 NHEFS | USA | 9632 M, W | 25-74 | 19 | 1802 | Medical records | Pinto beans, red beans, black-eye peas, peanuts, peanut butter | FFQ + 24-hr recall | Quartiles | ~5.9 | ~80.9 | Agency |
| Buckland et al. 2009 – M (27) | EPIC- Spain | Spain | 15 335 M | 29-69 | 10.4 | 480 | Medical records | Legumes | SFFQ, validated, interviewer administered | Tertiles | ~20.9 | ~295.6 | Agency |
| Buckland et al. 2009 – W (27) | EPIC- Spain | Spain | 25 422 W | 29-69 | 10.4 | 126 | Medical records | Legumes | SFFQ, validated, interviewer administered | Tertiles | ~15.1 | ~214.2 | Agency |
| Bernstein et al. 2010 (28) | NHS | USA | 84 136 W | 30-55 | 26 | 3162 | Medical records | Beans | SFFQ, validated | Quintiles | ~0 | ~16.4 | Agency |
| Martinez-Gonzalez et al. 2011 (29) | SUN | Spain | 5444 M,  8165 W | 18-101 | 4.9 | 68 | Medical records | Lentils, chickpeas, beans, peas (18) | SFFQ, validated | Median | NR | NR | Agency |
| Dilis et al. 2012 – M (30) | EPIC-Greece | Greece | 9740 M | 20-86 | 10 | 426 | Medical records | Lentils, chickpeas, beans, green peas, green beans (31) | SFFQ, validated, interviewer administered | per SD | NR | NR | Agency |
| Dilis et al. 2012 – W (30) | EPIC-Greece | Greece | 14 189 W | 20-86 | 10 | 210 | Medical records | Lentils, chickpeas, beans, green peas, green beans (31) | SFFQ, validated, interviewer administered | per SD | NR | NR | Agency |
| Haring et al. 2014 (32) | ARIC | USA | 5331 M,  6735 W | 45-64 | 22 | 1147 | Medical records | String beans or green beans, peas or lima beans, beans or lentils (dried cooked, or canned) (33) | SFFQ, interviewer administered | Quintiles | ~10.9 | ~65.1 | Agency |
| Yu et al. 2014 (34) | SMHS | China | 55 474 M | 40-74 | 5.4 | 217 | Medical records | Fresh peas, fresh soyabeans, broad beans, Chinese long beans, green beans, snow peas | SFFQ, validated, interviewer administered | Quartiles | 10.8 | 62.8 | Agency |
| Yu et al. 2014 (34) | SWHS | China | 67 211 W | 40-70 | 9.8 | 148 | Medical records | Fresh peas, fresh soyabeans, broad beans, Chinese long beans, green beans,snow peas | SFFQ, validated, interviewer administered | Quartiles | 8.5 | 50.7 | Agency |

AHS=Adventists Health Study; ARIC=Atherosclerosis Risk in Communities; CHD=coronary heart disease; EPIC=European Prospective Investigation into Cancer and Nutrition; FFQ=food frequency questionnaire; JPHC=Japan Public Health Center; M=men; NHANES 1 NHEFS=First National Health and Nutrition Examination Survey Epidemiologic Follow-up Study; NHS= Nurses’ Health Study; NR=not reported; PURE= Prospective Urban Rural Epidemiology; SD=standard deviation; SFFQ=semi-quantitative food frequency questionnaire; SMHS=Shanghai Men's Health Study; SUN=Seguimiento Universidad de Navarra; SWHS=Shanghai Women’s Health Study; W=women; y=years

^1^ For studies that reported “legumes” as the exposure without differentiating the legume type, an attempt was made to access the diet assessment tool used by the study or other relevant publications in order to verify the legume type. A reference was provided for those studies where we were able to obtain more information regarding the legume type.

^2^ Lowest and highest quantiles preceded by “~” represents quantiles that have been converted to g/d from another measure (e.g. servings/d were converted to g/d using a defined serving size provided by the study or the serving size was estimated using relevant dietary guidelines).

^3^ Agency funding is that from government, university, or not-for-profit sources. Industry funding is that from trade organizations that obtain revenue from the sale of products.

| **Study**  **(Supplemental reference)** | **Cohort** | **Country** | **No.**  **participants** | **Age range,**  **y** | **Follow-up,**  **y** | **No.**  **cases** | **Outcome**  **assessment**  **method** | **Exposure ^1^** | **Diet assessment method** | **Quantile**  **divisions** | **Lowest**  **quantile ^2^,**  **g/d** | **Highest**  **quantile ^2^,**  **g/d** | **Funding**  **source ^3^** |
| --- | --- | --- | --- | --- | --- | --- | --- | --- | --- | --- | --- | --- | --- |
| Fraser et al. 1992 (9) | AHS | USA | 10 003 M,  16 740 W | ≥25 | 6 | 463 | Medical records | Beans, peas | SFFQ, validated | Tertiles | NR | NR | Agency |
| Kelemen et al. 2005 (35) | IWHS | USA | 29 017 W | 55-69 | 15 | 739 | Medical records | Tofu, dried beans, nuts, peanut butter | SFFQ, validated | Quintiles | ~10.7 | ~74.7 | Agency |
| Nagura et al. 2009 (19) | JACC | Japan | 25 206 M,  34 279 W | 40-79 | 12.7 | 452 | Medical records | Tofu, boiled beans | FFQ, validated | Quartiles | ~5.7 | ~32.1 | Agency |
| Dilis et al. 2012 – M (30) | EPIC-Greece | Greece | 9740 M | 20-86 | 10 | 150 | Medical records | Lentils, chickpeas, beans, green peas, green beans (31) | SFFQ, validated, interviewer administered | per SD | NR | NR | Agency |
| Dilis et al. 2012 – W (30) | EPIC-Greece | Greece | 14 189 W | 20-86 | 10 | 90 | Medical records | Lentils, chickpeas, beans, green peas, green beans (31) | SFFQ, validated, interviewer administered | per SD | NR | NR | Agency |
| Wang et al. 2016 (36) | LINXIAN NIT | China | 1104 M,  1341 W | 40-69 | 26 | 355 | Medical records | Cooked dried soybean, cooked dried beans or peas, fresh bean curd, dried bean curd, fermented salted pickled bean curd, bean sprouts | FFQ | per 4 times/wk | NR | NR | Agency |
| Bonaccio et al. 2017 (23) | Moli-sani | Italy | 9931 M,  11 376 W | ≥35 | 8.3 | 92 | Medical records | Lentils, beans, chickpeas, fava beans, green peas | SFFQ, validated, interviewer administered | Quartiles | 8.3 | 53.8 | Agency-  Industry |
| Farvid et al. 2017 (24) | Golestan Cohort Study | Iran | 18 261 M,  24 142 W | 36-85 | 8.1 | 764 | Medical records | Soybeans, beans, lentils, peas, split peas | SFFQ, validated, interviewer administered | Quintiles | ~3 | ~26 | Agency |
| Stefler et al. 2017 (25) | HAPIEE Study | Czech Republic, Poland, Russia | 19 263 M,W | 57 | 7 | 226 | Medical records | Legumes | SFFQ, validated | per 1 point increase in component score | NR | NR | Agency |

## Supplemental Table 8 – Characteristics of prospective cohort studies assessing the association between dietary pulses with or without other legumes and CHD mortality (9 cohort comparisons, N=224 592)

AHS=Adventists Health Study; CHD=coronary heart disease; EPIC=European Prospective Investigation into Cancer and Nutrition; FFQ=food frequency questionnaire; HAPIEE=Health Alcohol and Psychosocial factors in Eastern Europe Study; IWHS=Iowa Women's Health Study; JACC=Japan Collaborative Cohort; M=men; NIT=Nutrition Intervention Trials; NR=not reported; SD=standard deviation; SFFQ=semi-quantitative food frequency questionnaire; W=women; wk=week; y=years

^1^ For studies that reported “legumes” as the exposure without differentiating the legume type, an attempt was made to access the diet assessment tool used by the study or other relevant publications in order to verify the legume type. A reference was provided for those studies where we were able to obtain more information regarding the legume type.

^2^ Lowest and highest quantiles preceded by “~” represents quantiles that have been converted to g/d from another measure (e.g. servings/d were converted to g/d using a defined serving size provided by the study or the serving size was estimated using relevant dietary guidelines).

^3^ Agency funding is that from government, university, or not-for-profit sources. Industry funding is that from trade organizations that obtain revenue from the sale of products.

| **Study (Supplemental reference)** | **Cohort** | **Country** | **No.**  **participants** | **Age range,**  **y** | **Follow-up,**  **y** | **No.**  **cases** | **Outcome**  **assessment**  **method** | **Exposure** | **Diet assessment method** | **Quantile**  **divisions** | **Lowest**  **quantile ^1^,**  **g/d** | **Highest**  **quantile ^1^,**  **g/d** | **Funding**  **source ^2^** |
| --- | --- | --- | --- | --- | --- | --- | --- | --- | --- | --- | --- | --- | --- |
| Fraser et al. 1992 (9) | AHS | USA | 10 003 M,  16 740 W | ≥25 | 6 | 134 | Medical records | Beans, peas | SFFQ, validated | Tertiles | NR | NR | Agency |
| Kokubo et al. 2007 – M (10) | JPHC | Japan | 19 466 M | 40-59 | 12.5 | 242 | Medical records | Beans | FFQ, validated | Tertiles | NR | NR | Agency |
| Kokubo et al. 2007 – W (10) | JPHC | Japan | 20 984 W | 40-59 | 12.5 | 66 | Medical records | Beans | FFQ, validated | Tertiles | NR | NR | Agency |
| Miller et al. 2017 (11) | PURE | 18  countries ^3^ | 56 422 M,  78 913 W | 35-70 | 7.4 | 2143 | Medical records | Beans, black beans, lentils, peas, chickpeas, black-eyed peas | FFQ, validated | Quintiles | ~0 | ~213 | Agency-  Industry |

## Supplemental Table 9 – Characteristics of prospective cohort studies assessing the association between dietary pulses with or without other legumes and MI incidence (4 cohort comparisons, N=202 528)

AHS=Adventists Health Study; FFQ=food frequency questionnaire; JPHC=Japan Public Health Center; M=men; NR=not reported; PURE= Prospective Urban Rural Epidemiology; SFFQ=semi-quantitative food frequency questionnaire; W=women; y=years

^1^ Lowest and highest quantiles preceded by “~” represents quantiles that have been converted to g/d from another measure (e.g. servings/d were converted to g/d using a defined serving size provided by the study or the serving size was estimated using relevant dietary guidelines).

^2^ Agency funding is that from government, university, or not-for-profit sources. Industry funding is that from trade organizations that obtain revenue from the sale of products.

^3^ Canada, Sweden, United Arab Emirates, Argentina, Brazil, Chile, Malaysia, Poland, South Africa, Turkey, China, Colombia, Iran, occupied Palestinian territory, Bangladesh, India, Pakistan, and Zimbabwe.

## Supplemental Table 10 – Characteristics of prospective cohort studies assessing the association between dietary pulses with or without other legumes and stroke incidence (8 cohort comparisons, N=342 079)

| **Study (Supplemental reference)** | **Cohort** | **Country** | **No.**  **participants** | **Age range,**  **y** | **Follow-up,**  **y** | **No.**  **cases** | **Outcome**  **assessment**  **method** | **Exposure ^1^** | **Diet assessment method** | **Quantile**  **divisions** | **Lowest**  **quantile ^2^,**  **g/d** | **Highest**  **quantile ^2^,**  **g/d** | **Funding**  **source ^3^** |
| --- | --- | --- | --- | --- | --- | --- | --- | --- | --- | --- | --- | --- | --- |
| Kokubo et al. 2007 – M (10) | JPHC | Japan | 19 466 M | 40-59 | 12.5 | 387 | Medical records | Beans | FFQ, validated | Tertiles | NR | NR | Agency |
| Kokubo et al. 2007 – W (10) | JPHC | Japan | 20 984 W | 40-59 | 12.5 | 200 | Medical records | Beans | FFQ. validated | Tertiles | NR | NR | Agency |
| Mizrahi et al. 2009 (37) | Finnish Mobile Clinic Health Examination Survey | Finland | 3932 M,W | 40-74 | 24 | 625 | Medical records | Legumes | Diet history interview | Quartiles | ~0.8 | ~40.3 | Agency |
| Bernstein et al. 2012 (38) | HPFS | USA | 43 150 M | 40-75 | 22 | 1397 | Medical records | Dry beans, peas, soy, tofu | SFFQ, validated | Quintiles | ~6.8 | ~55.3 | Agency |
| Bernstein et al. 2012 (38) | NHS | USA | 84 010 W | 30-55 | 26 | 2633 | Medical records | Dry beans, peas, soy, tofu | SFFQ, validated | Quintiles | ~6.8 | ~41.7 | Agency |
| Misirli et al. 2012 (39) | EPIC-Greece | Greece | 9617 M,  13 984 W | NR | 10.6 | 395 | Medical records | Lentils, chickpeas, beans, green peas, green beans (31) | SFFQ, validated, interviewer administered | per SD | NR | NR | Agency |
| Haring et al. 2015 (40) | ARIC | USA | 5120 M,  6481 W | 45-64 | 22.7 | 699 | Medical records | String beans or green beans, peas or lima beans, beans or lentils (dried cooked, or canned) (33) | SFFQ, interviewer administered | Quintiles | ~7.6 | ~61.8 | Agency |
| Miller et al. 2017 (11) | PURE | 18  countries ^4^ | 56 422 M,  78 913 W | 35-70 | 7.4 | 2234 | Medical records | Beans, black beans, lentils, peas, chickpeas, black-eyed peas | FFQ, validated | Quintiles | ~0 | ~213 | Agency-Industry |

ARIC=Atherosclerosis Risk in Communities; EPIC=European Prospective Investigation into Cancer and Nutrition; FFQ=food frequency questionnaire; HPFS=Health Professionals Follow-Up Study; JPHC=Japan Public Health Center; M=men; NHS=Nurses’ Health Study; NR=not reported; PURE=Prospective Urban Rural Epidemiology; SD=standard deviation; SFFQ=semi-quantitative food frequency questionnaire; W=women; y=years

^1^ For studies that reported “legumes” as the exposure without differentiating the legume type, an attempt was made to access the diet assessment tool used by the study or other relevant publications in order to verify the legume type. A reference was provided for those studies where we were able to obtain more information regarding the legume type.

^2^ Lowest and highest quantiles preceded by “~” represents quantiles that have been converted to g/d from another measure (e.g. servings/d were converted to g/d using a defined serving size provided by the study or the serving size was estimated using relevant dietary guidelines).

^3^ Agency funding is that from government, university, or not-for-profit sources. Industry funding is that from trade organizations that obtain revenue from the sale of products.

^4^ Canada, Sweden, United Arab Emirates, Argentina, Brazil, Chile, Malaysia, Poland, South Africa, Turkey, China, Colombia, Iran, occupied Palestinian territory, Bangladesh, India, Pakistan, and Zimbabwe.

## Supplemental Table 11 – Characteristics of prospective cohort studies assessing the association between dietary pulses with or without other legumes and stroke mortality (6 cohort comparisons, N=168 504)

| **Study**  **(Supplemental reference)** | **Cohort** | **Country** | **No.**  **participants** | **Age range,**  **y** | **Follow-up,**  **y** | **No.**  **cases** | **Outcome**  **assessment**  **method** | **Exposure ^1^** | **Diet assessment method** | **Quantile**  **divisions** | **Lowest**  **quantile ^2^,**  **g/d** | **Highest**  **quantile ^2^,**  **g/d** | **Funding**  **source ^3^** |
| --- | --- | --- | --- | --- | --- | --- | --- | --- | --- | --- | --- | --- | --- |
| Nagura et al. 2009 (19) | JACC | Japan | 25 206 M,  34 279 W | 40-79 | 12.7 | 1053 | Medical records | Tofu, boiled beans | FFQ, validated | Quartiles | ~5.7 | ~32.1 | Agency |
| Misirli et al. 2012 (39) | EPIC-Greece | Greece | 9617 M,  13 984 W | NR | 10.6 | 196 | Medical records | Lentils, chickpeas, beans, green peas, green beans (31) | SFFQ, validated, interviewer administered | per SD | NR | NR | Agency |
| Wang et al. 2016 (36) | LINXIAN NIT | China | 1104 M,  1341 W | 40-69 | 26 | 452 | Medical records | Beans | FFQ | per 4 times/wk | NR | NR | Agency |
| Bonaccio et al. 2017 (23) | Moli-sani | Italy | 9931 M,  11 376 W | ≥35 | 8.3 | 67 | Medical records | Lentils, beans, chickpeas, fava beans, green peas | SFFQ, validated, interviewer administered | Quartiles | 8.3 | 53.8 | Agency-  Industry |
| Farvid et al. 2017 (24) | Golestan Cohort Study | Iran | 18 261 M,  24 142 W | 36-85 | 8.1 | 507 | Medical records | Soybeans, beans, lentils, peas, split peas | SFFQ, validated, interviewer administered | Quintiles | ~3 | ~26 | Agency |
| Stefler et al. 2017 (25) | HAPIEE Study | Czech Republic, Poland, Russia | 19 263 M,W | 57 | 7 | 109 | Medical records | Legumes | SFFQ, validated | per 1 point increase in component score | NR | NR | Agency |

EPIC=European Prospective Investigation into Cancer and Nutrition; FFQ=food frequency questionnaire; HAPIEE=Health Alcohol and Psychosocial factors in Eastern Europe Study; JACC=Japan Collaborative Cohort; ; M=men; NIT=Nutrition Intervention Trials; NR=not reported; SD=standard deviation; SFFQ=semi-quantitative food frequency questionnaire; W=women; y=years; wk=week

^1^ For studies that reported “legumes” as the exposure without differentiating the legume type, an attempt was made to access the diet assessment tool used by the study or other relevant publications in order to verify the legume type. A reference was provided for those studies where we were able to obtain more information regarding the legume type.

^2^ Lowest and highest quantiles preceded by “~” represents quantiles that have been converted to g/d from another measure (e.g. servings/d were converted to g/d using a defined serving size provided by the study or the serving size was estimated using relevant dietary guidelines).

^3^ Agency funding is that from government, university, or not-for-profit sources. Industry funding is that from trade organizations that obtain revenue from the sale of products.

## Supplemental Table 12 – Characteristics of prospective cohort studies assessing the association between dietary pulses with or without other legumes and diabetes incidence (9 cohort comparisons, N=259 325)

| **Study**  **(Supplemental reference)** | **Cohort** | **Country** | **No. participants** | **Age range, y** | **Follow-up,**  **y** | **No.**  **cases** | **Outcome**  **assessment**  **method** | **Exposure ^1^** | **Diet assessment method** | **Quantile**  **divisions** | **Lowest**  **quantile ^2^,**  **g/d** | **Highest**  **quantile ^2^,**  **g/d** | **Funding**  **source ^3^** |
| --- | --- | --- | --- | --- | --- | --- | --- | --- | --- | --- | --- | --- | --- |
| Meyer et al. 2000 (41) | IWHS | USA | 35 988 W | 55-69 | 6 | 1141 | Self-report | Mature beans | SFFQ, validated | Quintiles | ~19.3 | ~125.4 | Agency |
| Hodge et al. 2004 (42) | MCCS | Australia | 31 641 M, W | 40-69 ^4^ | 4 | 365 | Medical records | Lentils, chickpeas, dry beans, dry peas, baked beans, green beans, green peas (21) | FFQ | Quartiles | NR | NR | Agency |
| Bazzano et al. 2008 (43) | NHS | USA | 71 346 W | 38-63 | 18 | 4529 | Self-report | Tofu, peas, beans | SFFQ, validated | Quintiles | ~7.3 | ~46.9 | Agency |
| Villegas et al. 2008 (44) | SWHS | China | 64 191 W | 40-70 | 4.6 | 1605 | Self-report | All legumes excluding soybeans and peanuts | SFFQ, validated, interviewer administered | Quintiles | 5.6 | 37.1 | Agency |
| Ericson et al. 2013 – M (45) | MDC | Sweden | 10 550 M | 45-74 | 12 | 798 | Medical records | Legumes | Modified diet history method ^5^, validated | Quintiles | 0 | 61 | Agency-Industry |
| Ericson et al. 2013 – W (45) | MDC | Sweden | 16 590 W | 45-74 | 12 | 773 | Medical records | Legumes | Modified diet history method ^5^, validated | Quintiles | 0 | 49 | Agency-Industry |
| von Ruesten et al. 2013 (15) | EPIC-Potsdam | Germany | 9098 M,  14 433 W | 35-65 | 8 | 837 | Medical records | Green beans, green peas, lentil/pea/  bean stew | SFFQ, validated | per 100 g/d | NR | NR | Agency |
| Becerra-Tomas et al. 2017 (46) | PREDIMED | Spain | 1267 M,  2082 W | 55-80 M  60-80 W | 4.3 | 266 | Medical records | Lentils, chickpeas, dry beans, fresh peas | SFFQ, validated, interviewer administered | Quartiles | 12.7 | 28.8 | Agency-Industry |
| Khalili-Moghadam et al. 2019 (47) | TLGS | Iran | 971 M,  1168 W | 20-70 | 5.8 | 143 | NR | Legumes | SFFQ, validated, interviewer administered | Tertiles | ~20.2 | ~41.9 | NR |

EPIC=European Prospective Investigation into Cancer and Nutrition; FFQ=food frequency questionnaire; IWHS=Iowa Women's Health Study; M=men; MCCS=Melbourne Collaborative Cohort Study; MDC=Malmo Diet and Cancer; NHS=Nurses’ Health Study; NR=not reported; PREDIMED=PREvencion con DIeta MEDiterranea study; SFFQ=semi-quantitative food frequency questionnaire; SWHS=Shanghai Women's Health Study; TLGS=Tehran Lipid and Glucose Study; W=women; y=years

^1^ For studies that reported “legumes” as the exposure without differentiating the legume type, an attempt was made to access the diet assessment tool used by the study or other relevant publications in order to verify the legume type. A reference was provided for those studies where we were able to obtain more information regarding the legume type.

^2^ Lowest and highest quantiles preceded by “~” represents quantiles that have been converted to g/d from another measure (e.g. servings/d were converted to g/d using a defined serving size provided by the study or the serving size was estimated using relevant dietary guidelines).

^3^ Agency funding is that from government, university, or not-for-profit sources. Industry funding is that from trade organizations that obtain revenue from the sale of products.

^4^ Participants age range at baseline was 27-75 years, where 99% of participants age ranged 40-69 years.

^5^ 7-day menu book + SFFQ + interview.

| **Study**  **(Supplemental reference)** | **Cohort** | **Country** | **No.**  **participants** | **Age range,**  **y** | **Follow-up,**  **y** | **No.**  **cases** | **Outcome**  **Assessment**  **method** | **Exposure ^1^** | **Diet assessment method** | **Quantile**  **divisions** | **Lowest**  **quantile ^2^,**  **g/d** | **Highest**  **quantile ^2^,**  **g/d** | **Funding**  **source ^3^** |
| --- | --- | --- | --- | --- | --- | --- | --- | --- | --- | --- | --- | --- | --- |
| Nunez-Cordoba et al. 2009 (48) | SUN | Spain | 3583 M,  5825 W | 20-90 | 4.2 | 501 | Medical records | Lentils, chickpeas, beans, peas (18) | SFFQ, validated | Median | NR | NR | Agency |
| Weng et al. 2013 (49) | ARIC | USA | 4459 M,  5454 W | 45-64 | 9 | 2853 | Medical records | String beans or green beans, peas or lima beans, beans or lentils (dried cooked, or canned) (33) | SFFQ, interviewer administered | Tertiles | ~0 | ~162.8 | Agency |
| Borgi et al. 2016 (50) | NHS | USA | 62 175 W | 30-55 | 26 | 35375 | Self-report | Beans or lentils | SFFQ, validated | Quartiles | ~2 | ~75.2 | Agency |
| Borgi et al. 2016 (50) | NHS II | USA | 88 475 W | 25-42 | 20 | 25246 | Self-report | Beans or lentils | SFFQ, validated | Quartiles | ~2 | ~75.2 | Agency |
| Borgi et al. 2016 (50) | HPFS | USA | 36 803 M | 40-75 | 24 | 16752 | Self-report | Beans or lentils | SFFQ, validated | Quartiles | ~2 | ~75.2 | Agency |
| Golzarand et al. 2016 (51) | TLGS | Iran | 490 M,  662 W | 20-84 | 3 | 144 | Medical records | Kidney beans, pinto beans, white beans, chickpeas,  cowpeas, lentils, split peas, soybeans | SFFQ, validated, interviewer administered | Tertiles | 13.5 | 43.9 | Agency |
| Lelong et al. 2017 (52) | NutriNet-Santé | France | 16 166 M,  64 260 W | ≥18 | 3.4 | 2413 | Self-report | Legumes | 24-hr diet records | Quartiles | 0 | 79 | Agency |

## Supplemental Table 13 – Characteristics of prospective cohort studies assessing the association between dietary pulses with or without other legumes and hypertension incidence (7 cohort comparisons, N=288 352)

ARIC=Atherosclerosis Risk in Communities; HPFS=Health Professionals Follow-Up Study; M=men; NHS=Nurses’ Health Study; NR=not reported; SFFQ=semi-quantitative food frequency questionnaire; SUN=Seguimiento Universidad de Navarra; TLGS=Tehran Lipid and Glucose Study; W=women; y=years

^1^ For studies that reported “legumes” as the exposure without differentiating the legume type, an attempt was made to access the diet assessment tool used by the study or other relevant publications in order to verify the legume type. A reference was provided for those studies where we were able to obtain more information regarding the legume type.

^2^ Lowest and highest quantiles preceded by “~” represents quantiles that have been converted to g/d from another measure (e.g. servings/d were converted to g/d using a defined serving size provided by the study or the serving size was estimated using relevant dietary guidelines).

^3^ Agency funding is that from government, university, or not-for-profit sources. Industry funding is that from trade organizations that obtain revenue from the sale of products.

## Supplemental Table 14 – Characteristics of prospective cohort studies assessing the association between dietary pulses with or without other legumes and obesity incidence (1 cohort comparison, N=18 146)

| **Study**  **(Supplemental reference)** | **Cohort** | **Country** | **No.**  **participants** | **Age range,**  **y** | **Follow-up,**  **y** | **No.**  **cases** | **Outcome**  **Assessment**  **method** | **Exposure** | **Diet assessment method** | **Quantile**  **divisions** | **Lowest**  **quantile ^1^,**  **g/d** | **Highest**  **quantile ^1^,**  **g/d** | **Funding**  **source ^2^** |
| --- | --- | --- | --- | --- | --- | --- | --- | --- | --- | --- | --- | --- | --- |
| Rautiainen et al. 2015 (53) | WHS | USA | 18 146 W | ≥45 | 15.9 | 8125 | Self-report | Beans, peas, string beans, tofu | SFFQ, validated | Quintiles | ~16.2 | ~75.8 | Agency |

SFFQ=semi-quantitative food frequency questionnaire; W=women; WHS=Women’s Health Study; y=years

^1^ Lowest and highest quantiles preceded by “~” represents quantiles that have been converted to g/d from another measure (e.g. servings/d were converted to g/d using a defined serving size provided by the study or the serving size was estimated using relevant dietary guidelines).

^2^ Agency funding is that from government, university, or not-for-profit sources. Industry funding is that from trade organizations that obtain revenue from the sale of products.

## Supplemental Table 15 – Analysis of confounding variables among prospective cohort studies assessing the association between dietary pulses with or without other legumes and CVD incidence

| **COHORT** | **NHANES 1 NHEFS** | **JPHC** | **EPIC-Potsdam** | **ICS** | **SUN** | **PURE** |
| --- | --- | --- | --- | --- | --- | --- |
| **STUDY (Supplemental reference)** | **Bazzano et al. 2001 (14)** | **Kokubo et al. 2007 (10)** | **von Ruesten et al. 2013 (15)** | **Nouri et al. 2016 (16)** | **Buil-Cosailes et al. 2017 (17)** | **Miller et al. 2017 (11)** |
| **Pre-specified primary confounding variables** |  |  |  |  |  |  |
| Age | x | x | x | x | x | x |
| **Pre-specified secondary confounding variables** |  |  |  |  |  |  |
| Sex | x | x | x | x | x | x |
| Family history of CVD |  |  |  |  | x |  |
| Smoking | x | x | x | x | x | x |
| Markers of overweight/obesity  (BMI, weight, waist circumference, waist-to-hip ratio) | x | x | x | x | x | x |
| Diabetes ^1^ | x | x |  |  | x | x |
| Hypertension ^1^ | x | x | x |  | x | x |
| Dyslipidemia ^1^ | x | x | x | x | x | x |
| Energy intake | x | x | x |  | x | x |
| Physical activity | x | x | x | x | x | x |
| **Other confounding variables** |  |  |  |  |  |  |
| Ethnicity, race, country of birth | x |  |  |  |  |  |
| Education | x | x | x | x | x | x |
| Income, job |  |  |  | x |  |  |
| Household amenities score |  |  |  |  |  |  |
| Marital status |  |  |  | x | x |  |
| Hours of sleep |  |  |  |  |  |  |
| Mental status |  |  |  |  |  |  |
| Height |  |  |  |  |  |  |
| Shared frailty |  |  |  | x |  |  |
| Family history of cancer |  |  |  |  |  |  |
| Family history of hypertension |  |  |  |  |  |  |
| History of hypercholesterolemia or hypertension |  |  |  |  |  |  |
| Menopausal status |  | x |  |  |  |  |
| Postmenopausal hormone use |  |  |  |  |  |  |
| Aspirin, analgesic use |  |  |  |  |  |  |
| Opium use |  |  |  |  |  |  |
| Oral contraceptive use |  |  |  |  |  |  |
| C-reactive protein |  |  |  |  |  |  |
| Carbohydrates |  |  |  |  |  |  |
| Grains |  |  |  |  | x | x |
| Fruits, vegetables | x | x |  |  | x |  |
| Fiber |  |  |  |  |  |  |
| Meat, poultry, fish | x | x |  |  |  | x |
| Egg |  |  |  |  |  |  |
| Dairy |  |  |  |  |  |  |
| Nuts |  |  |  |  |  |  |
| Other protein sources |  |  |  |  |  |  |
| No legume consumption |  |  | x |  |  |  |
| Fat |  |  |  |  |  |  |
| PUFA |  |  |  |  |  |  |
| MUFA |  |  |  |  | x |  |
| SFA | x |  |  |  |  |  |
| Trans fat |  |  |  |  |  |  |
| MUFA:SFA |  |  |  |  |  |  |
| Dietary cholesterol |  |  |  |  |  |  |
| Dietary methionine |  |  |  |  |  |  |
| Vitamin supplements use |  |  | x |  |  |  |
| Magnesium |  |  |  |  |  |  |
| Potassium |  |  |  |  |  |  |
| Sodium |  | x |  |  |  |  |
| Alcohol | x | x | x |  | x |  |
| Tea, coffee |  |  |  |  |  |  |
| Water |  |  |  |  |  |  |
| SSBs, ASBs, fruit juice |  |  |  |  |  |  |
| Junk food (chips, pizza, desserts) |  |  |  |  |  |  |
| Legume-adjusted dietary score |  |  |  | x |  |  |
| MedDiet adherence |  |  |  |  |  |  |
| Healthy Food Score |  |  |  |  |  |  |
| Centre |  | x |  |  |  | x |
| Birth cohort, cohort | x |  |  |  |  |  |
| Time period, season |  |  |  |  |  |  |
| Area of recruitment, residency |  |  |  |  |  | x |
| Method version |  |  |  |  |  |  |
| Intervention group |  |  |  |  |  |  |
| Randomization treatment assignment |  |  |  |  |  |  |

ASB=artificially sweetened beverage; CVD=cardiovascular disease; EPIC=European Prospective Investigation into Cancer and Nutrition; ICS=Isfahan Cohort Study; JPHC=Japan Public Health Center; NHANES 1 NHEFS=First National Health and Nutrition Examination Survey Epidemiologic Follow-up Study; PURE=Prospective Urban Rural Epidemiology; SSB=sugar sweetened beverage; SUN=Seguimiento Universidad de Navarra

^1^ Includes adjustment for medication use for respective condition or levels of relevant biomarkers

## Supplemental Table 16 – Analysis of confounding variables among prospective cohort studies assessing the association between dietary pulses with or without other legumes and CVD mortality

| **COHORT** | **JPHC** | **JACC** | **NOMAS** | **MCCS** | **EPIC** | **Moli-sani** | **Golestan Cohort Study** | **PURE** | **HAPIEE Study** | **PREDIMED** | **The Netherlands Cohort Study** |
| --- | --- | --- | --- | --- | --- | --- | --- | --- | --- | --- | --- |
| **STUDY (Supplemental reference)** | **Kokubo et al. 2007 (10)** | **Nagura et al. 2009 (19)** | **Gardener et al. 2011 (20)** | **Russell et al. 2014 (12)** | **Lassale et al. 2015 (22)** | **Bonaccio et al. 2017 (23)** | **Farvid et al. 2017 (24)** | **Miller et al. 2017 (11)** | **Stefler et al. 2017 (25)** | **Papandreou et al. 2018 (26)** | **van den Brandt et al. 2019 (13)** |
| **Pre-specified primary confounding variables** |  |  |  |  |  |  |  |  |  |  |  |
| Age | x | x | x | x | x | x | x | x | x | x | x |
| **Pre-specified secondary confounding variables** |  |  |  | NR ^1^ |  |  |  |  |  |  |  |
| Sex | x | x | x |  | x | x | x | x | x | x | x |
| Family history of CVD |  |  |  |  |  |  |  |  |  |  |  |
| Smoking | x | x | x |  | x | x | x | x | x | x | x |
| Markers of overweight/obesity  (BMI, weight, waist circumference, waist-to-hip ratio) | x | x |  |  | x |  | x | x |  | x | x |
| Diabetes ^2^ | x | x |  |  |  | x |  | x |  | x | x |
| Hypertension ^2^ | x | x |  |  |  |  | x | x |  | x | x |
| Dyslipidemia ^2^ | x |  |  |  |  |  |  | x |  | x |  |
| Energy intake | x |  | x |  | x | x | x | x | x |  | x |
| Physical activity | x | x | x |  | x | x | x | x | x | x | x |
| **Other confounding variables** |  |  |  |  |  |  |  |  |  |  |  |
| Ethnicity, race, country of birth |  |  | x |  |  |  | x |  |  |  |  |
| Education | x | x | x |  | x | x | x | x | x | x | x |
| Income, job |  |  |  |  |  | x | x |  |  |  |  |
| Household amenities score |  |  |  |  |  |  |  |  | x |  |  |
| Marital status |  |  |  |  |  |  | x |  | x |  |  |
| Hours of sleep |  | x |  |  |  |  |  |  |  |  |  |
| Mental status |  | x |  |  |  |  |  |  |  |  |  |
| Height |  |  |  |  |  |  |  |  |  |  | x |
| Shared frailty |  |  |  |  |  |  |  |  |  |  |  |
| Family history of cancer |  |  |  |  |  |  | x |  |  |  |  |
| Family history of hypertension |  |  |  |  |  |  |  |  |  |  |  |
| History of hypercholesterolemia or hypertension |  |  |  |  |  |  |  |  |  |  |  |
| Menopausal status | x |  |  |  |  |  |  |  |  |  |  |
| Postmenopausal hormone use |  |  |  |  |  |  |  |  |  |  | x |
| Aspirin, analgesic use |  |  |  |  |  |  | x |  |  |  |  |
| Opium use |  |  |  |  |  |  | x |  |  |  |  |
| Oral contraceptive use |  |  |  |  |  |  |  |  |  |  |  |
| C-reactive protein |  |  |  |  |  | x |  |  |  |  |  |
| Carbohydrates |  |  |  |  |  |  |  |  |  |  |  |
| Grains |  |  | x |  |  |  |  | x |  |  |  |
| Fruits, vegetables | x | x | x |  |  |  |  |  |  |  | x |
| Fiber |  |  |  |  |  |  |  |  |  |  |  |
| Meat, poultry, fish | x |  | x |  |  |  |  | x |  |  |  |
| Egg |  |  |  |  |  |  |  |  |  |  |  |
| Dairy |  |  | x |  |  |  |  |  |  |  |  |
| Nuts |  |  |  |  |  |  |  |  |  |  |  |
| Other protein sources |  |  |  |  |  |  |  |  |  |  |  |
| No legume consumption |  |  |  |  |  |  |  |  |  |  |  |
| Fat |  |  |  |  |  |  |  |  |  |  |  |
| PUFA |  | x |  |  |  |  |  |  |  |  |  |
| MUFA |  |  |  |  |  |  |  |  |  |  |  |
| SFA |  | x |  |  |  |  |  |  |  |  |  |
| Trans fat |  |  |  |  |  |  |  |  |  |  |  |
| MUFA:SFA |  |  | x |  |  |  |  |  |  |  |  |
| Dietary cholesterol |  | x |  |  |  |  |  |  |  |  |  |
| Dietary methionine |  |  |  |  |  |  |  |  |  |  |  |
| Vitamin supplements use |  |  |  |  |  |  |  |  | x |  | x |
| Magnesium |  |  |  |  |  |  |  |  |  |  |  |
| Potassium |  |  |  |  |  |  |  |  |  |  |  |
| Sodium | x | x |  |  |  |  |  |  |  |  |  |
| Alcohol | x | x | x |  | x |  | x |  |  | x | x |
| Tea, coffee |  |  |  |  |  | x |  |  |  |  |  |
| Water |  |  |  |  |  |  |  |  |  |  |  |
| SSBs, ASBs, fruit juice |  |  |  |  |  |  |  |  |  |  |  |
| Junk food (chips, pizza, desserts) |  |  |  |  |  |  |  |  |  |  |  |
| Legume-adjusted dietary score |  |  |  |  |  | x |  |  |  |  |  |
| MedDiet adherence |  |  |  |  |  |  |  |  |  | x |  |
| Healthy Food Score |  |  |  |  |  |  |  |  |  |  |  |
| Centre | x |  |  |  | x |  |  | x |  | x |  |
| Birth cohort, cohort |  |  |  |  |  |  |  |  | x |  |  |
| Time period, season |  |  |  |  |  | x |  |  |  |  |  |
| Area of recruitment, residency |  |  |  |  |  |  | x | x |  |  |  |
| Method version |  |  |  |  |  |  |  |  |  |  |  |
| Intervention group |  |  |  |  |  |  |  |  |  | x |  |
| Randomization treatment assignment |  |  |  |  |  |  |  |  |  |  |  |

ASB=artificially sweetened beverage; CVD=cardiovascular disease; EPIC=European Prospective Investigation into Cancer and Nutrition; HAPIEE=Health Alcohol and Psychosocial factors in Eastern Europe Study; JACC=Japan Collaborative Cohort; JPHC=Japan Public Health Center; MCCS=Melbourne Collaborative Cohort Study; NOMAS=Northern Manhattan Study; NR=not reported; PREDIMED=PREvencion con DIeta MEDiterranea study; PURE= Prospective Urban Rural Epidemiology; SSB=sugar sweetened beverage

^1^ Study results were reported in an abstract, which stated that “Multivariate regression models were used to calculate cardiovascular mortality hazard risk ratios. Models were adjusted for age, and other lifestyle variables.”

^2^ Includes adjustment for medication use for respective condition or levels of relevant biomarkers

## Supplemental Table 17 – Analysis of confounding variables among prospective cohort studies assessing the association between dietary pulses with or without other legumes and CHD incidence

| **COHORT** | **NHANES 1 NHEFS** | **EPIC-Spain** | **NHS** | **SUN** | **EPIC-Greece** | **ARIC** | **SMHS** | **SWHS** |
| --- | --- | --- | --- | --- | --- | --- | --- | --- |
| **STUDY (Supplemental reference)** | **Bazzano et al. 2001 (14)** | **Buckland et al. 2009 (27)** | **Bernstein et al. 2010 (28)** | **Martinez-Gonzalez et al. 2011 (29)** | **Dilis et al. 2012 (30)** | **Haring et al. 2014 (32)** | **Yu et al. 2014 (34)** | **Yu et al. 2014 (34)** |
| **Pre-specified primary confounding variables** |  |  |  |  |  |  |  |  |
| Age | x | x | x | x | x | x | x | x |
| **Pre-specified secondary confounding variables** |  |  |  |  |  |  |  |  |
| Sex | x | x | x | x | x | x | x | x |
| Family history of CVD |  |  | x | x |  |  |  |  |
| Smoking | x | x | x | x | x | x | x | x |
| Markers of overweight/obesity  (BMI, weight, waist circumference, waist-to-hip ratio) | x | x | x | x | x | x | x | x |
| Diabetes ^1^ | x | x |  | x |  |  | x | x |
| Hypertension ^1^ | x | x |  | x | x | x | x | x |
| Dyslipidemia ^1^ | x | x |  | x |  | x | x | x |
| Energy intake | x | x | x | x | x | x | x | x |
| Physical activity | x | x | x | x | x | x | x | x |
| **Other confounding variables** |  |  |  |  |  |  |  |  |
| Ethnicity, race, country of birth | x |  |  |  |  | x |  |  |
| Education | x | x |  |  | x | x | x | x |
| Income, job |  |  |  |  |  |  | x | x |
| Household amenities score |  |  |  |  |  |  |  |  |
| Marital status |  |  |  |  |  |  |  |  |
| Hours of sleep |  |  |  |  |  |  |  |  |
| Mental status |  |  |  |  |  |  |  |  |
| Height |  |  |  |  | x |  |  |  |
| Shared frailty |  |  |  |  |  |  |  |  |
| Family history of cancer |  |  |  |  |  |  |  |  |
| Family history of hypertension |  |  |  |  |  |  |  |  |
| History of hypercholesterolemia or hypertension |  |  |  |  |  |  |  |  |
| Menopausal status |  |  | x |  |  |  | x | x |
| Postmenopausal hormone use |  |  | x |  |  |  | x | x |
| Aspirin, analgesic use |  |  | x | x |  |  | x | x |
| Opium use |  |  |  |  |  |  |  |  |
| Oral contraceptive use |  |  |  |  |  |  |  |  |
| C-reactive protein |  |  |  |  |  |  |  |  |
| Carbohydrates |  |  |  |  |  | x |  |  |
| Grains |  |  |  |  |  |  |  |  |
| Fruits, vegetables | x |  |  |  |  |  |  |  |
| Fiber |  |  | x |  |  | x |  |  |
| Meat, poultry, fish | x |  |  |  |  |  | x | x |
| Egg |  |  |  |  |  |  |  |  |
| Dairy |  |  |  |  |  |  |  |  |
| Nuts |  |  |  |  |  |  |  |  |
| Other protein sources |  |  |  |  |  |  |  |  |
| No legume consumption |  |  |  |  |  |  |  |  |
| Fat |  |  |  |  |  |  |  |  |
| PUFA |  |  |  |  |  |  |  |  |
| MUFA |  |  |  |  |  |  |  |  |
| SFA | x |  |  |  |  |  |  |  |
| Trans fat |  |  | x |  |  |  |  |  |
| MUFA:SFA |  |  |  |  |  |  |  |  |
| Dietary cholesterol |  |  |  |  |  |  |  |  |
| Dietary methionine |  |  |  |  |  |  |  |  |
| Vitamin supplements use |  |  | x |  |  |  | x | x |
| Magnesium |  |  |  |  |  | x |  |  |
| Potassium |  |  |  |  |  |  |  |  |
| Sodium |  |  |  |  |  |  |  |  |
| Alcohol | x |  | x |  | x | x | x | x |
| Tea, coffee |  |  |  |  |  |  |  |  |
| Water |  |  |  |  |  |  |  |  |
| SSBs, ASBs, fruit juice |  |  |  |  |  |  |  |  |
| Junk food (chips, pizza, desserts) |  |  |  |  |  |  |  |  |
| Legume-adjusted dietary score |  |  |  |  |  |  |  |  |
| MedDiet adherence |  |  |  |  |  |  |  |  |
| Healthy Food Score |  |  |  |  |  |  |  |  |
| Centre |  | x |  |  |  | x |  |  |
| Birth cohort, cohort | x |  |  |  |  |  | x | x |
| Time period, season |  |  | x |  |  |  |  |  |
| Area of recruitment, residency |  |  |  |  |  |  |  |  |
| Method version |  |  |  |  |  |  |  |  |
| Intervention group |  |  |  |  |  |  |  |  |
| Randomization treatment assignment |  |  |  |  |  |  |  |  |

ARIC=Atherosclerosis Risk in Communities; ASB=artificially sweetened beverage; CHD=coronary heart disease; EPIC=European Prospective Investigation into Cancer and Nutrition; NHANES 1 NHEFS=First National Health and Nutrition Examination Survey Epidemiologic Follow-up Study; NHS=Nurses’ Health Study; SMHS=Shanghai Men's Health Study; SSB=sugar sweetened beverage; SUN=Seguimiento Universidad de Navarra; SWHS=Shanghai Women’s Health Study

^1^ Includes adjustment for medication use for respective condition or levels of relevant biomarkers

## Supplemental Table 18 – Analysis of confounding variables among prospective cohort studies assessing the association between dietary pulses with or without other legumes and CHD mortality

| **COHORT** | **AHS** | **IWHS** | **JACC** | **EPIC-Greece** | **LINXIAN NIT** | **Moli-sani** | **Golestan Cohort Study** | **HAPIEE Study** |
| --- | --- | --- | --- | --- | --- | --- | --- | --- |
| **STUDY (Supplemental reference)** | **Fraser et al. 1992 (9)** | **Kelemen et al. 2005 (35)** | **Nagura et al. 2009 (19)** | **Dilis et al. 2012 (30)** | **Wang et al. 2016 (36)** | **Bonaccio et al. 2017 (23)** | **Farvid et al. 2017 (24)** | **Stefler et al. 2017 (25)** |
| **Pre-specified primary confounding variables** |  |  |  |  |  |  |  |  |
| Age | x | x | x | x | x | x | x | x |
| **Pre-specified secondary confounding variables** |  |  |  |  |  |  |  |  |
| Sex | x | x | x | x | x | x | x | x |
| Family history of CVD |  |  |  |  |  |  |  |  |
| Smoking | x | x | x | x | x | x | x | x |
| Markers of overweight/obesity  (BMI, weight, waist circumference, waist-to-hip ratio) | x | x | x | x | x |  | x |  |
| Diabetes ^1^ |  |  | x |  |  | x |  |  |
| Hypertension ^1^ | x | x | x | x |  |  | x |  |
| Dyslipidemia ^1^ |  |  |  |  |  |  |  |  |
| Energy intake |  | x |  | x |  | x | x | x |
| Physical activity | x | x | x | x |  | x | x | x |
| **Other confounding variables** |  |  |  |  |  |  |  |  |
| Ethnicity, race, country of birth |  |  |  |  |  |  | x |  |
| Education |  | x | x | x |  | x | x | x |
| Income, job |  |  |  |  |  | x | x |  |
| Household amenities score |  |  |  |  |  |  |  | x |
| Marital status |  |  |  |  |  |  | x | x |
| Hours of sleep |  |  | x |  |  |  |  |  |
| Mental status |  |  | x |  |  |  |  |  |
| Height |  |  |  | x |  |  |  |  |
| Shared frailty |  |  |  |  |  |  |  |  |
| Family history of cancer |  | x |  |  |  |  | x |  |
| Family history of hypertension |  |  |  |  |  |  |  |  |
| History of hypercholesterolemia or hypertension |  |  |  |  |  |  |  |  |
| Menopausal status |  |  |  |  |  |  |  |  |
| Postmenopausal hormone use |  | x |  |  |  |  |  |  |
| Aspirin, analgesic use |  |  |  |  |  |  | x |  |
| Opium use |  |  |  |  |  |  | x |  |
| Oral contraceptive use |  |  |  |  |  |  |  |  |
| C-reactive protein |  |  |  |  |  | x |  |  |
| Carbohydrates |  |  |  |  |  |  |  |  |
| Grains |  |  |  |  |  |  |  |  |
| Fruits, vegetables |  | x | x |  |  |  |  |  |
| Fiber |  | x |  |  |  |  |  |  |
| Meat, poultry, fish |  | x |  |  |  |  |  |  |
| Egg |  | x |  |  |  |  |  |  |
| Dairy |  | x |  |  |  |  |  |  |
| Nuts |  |  |  |  |  |  |  |  |
| Other protein sources |  |  |  |  |  |  |  |  |
| No legume consumption |  |  |  |  |  |  |  |  |
| Fat |  |  |  |  |  |  |  |  |
| PUFA |  | x | x |  |  |  |  |  |
| MUFA |  | x |  |  |  |  |  |  |
| SFA |  | x | x |  |  |  |  |  |
| Trans fat |  | x |  |  |  |  |  |  |
| MUFA:SFA |  |  |  |  |  |  |  |  |
| Dietary cholesterol |  | x | x |  |  |  |  |  |
| Dietary methionine |  | x |  |  |  |  |  |  |
| Vitamin supplements use |  | x |  |  |  |  |  | x |
| Magnesium |  |  |  |  |  |  |  |  |
| Potassium |  |  |  |  |  |  |  |  |
| Sodium |  |  | x |  |  |  |  |  |
| Alcohol |  | x | x | x | x |  | x |  |
| Tea, coffee |  |  |  |  |  | x |  |  |
| Water |  |  |  |  |  |  |  |  |
| SSBs, ASBs, fruit juice |  |  |  |  |  |  |  |  |
| Junk food (chips, pizza, desserts) |  |  |  |  |  |  |  |  |
| Legume-adjusted dietary score |  |  |  |  |  | x |  |  |
| MedDiet adherence |  |  |  |  |  |  |  |  |
| Healthy Food Score |  |  |  |  |  |  |  |  |
| Centre |  |  |  |  |  |  |  |  |
| Birth cohort, cohort |  |  |  |  |  |  |  | x |
| Time period, season |  |  |  |  | x | x |  |  |
| Area of recruitment, residency |  |  |  |  | x |  | x |  |
| Method version |  |  |  |  |  |  |  |  |
| Intervention group |  |  |  |  |  |  |  |  |
| Randomization treatment assignment |  |  |  |  |  |  |  |  |

AHS=Adventists Health Study; ASB=artificially sweetened beverage; CHD=coronary heart disease; EPIC=European Prospective Investigation into Cancer and Nutrition; HAPIEE=Health Alcohol and Psychosocial factors in Eastern Europe Study; IWHS=Iowa Women's Health Study; JACC=Japan Collaborative Cohort; LINXIAN NIT=LINXIAN Nutrition Intervention Trials; SSB=sugar sweetened beverage

^1^ Includes adjustment for medication use for respective condition or levels of relevant biomarkers

## Supplemental Table 19 – Analysis of confounding variables among prospective cohort studies assessing the association between dietary pulses with or without other legumes and MI incidence

| **COHORT** | **AHS** | **JPHC** | **PURE** |
| --- | --- | --- | --- |
| **STUDY (Supplemental reference)** | **Fraser et al. 1992 (9)** | **Kokubo et al. 2007 (10)** | **Miller et al. 2017 (11)** |
| **Pre-specified primary confounding variables** |  |  |  |
| Age | x | x | x |
| **Pre-specified secondary confounding variables** |  |  |  |
| Sex | x | x | x |
| Family history of CVD |  |  |  |
| Smoking | x | x | x |
| Markers of overweight/obesity  (BMI, weight, waist circumference, waist-to-hip ratio) | x | x | x |
| Diabetes ^1^ |  | x | x |
| Hypertension ^1^ | x | x | x |
| Dyslipidemia ^1^ |  | x | x |
| Energy intake |  | x | x |
| Physical activity | x | x | x |
| **Other confounding variables** |  |  |  |
| Ethnicity, race, country of birth |  |  |  |
| Education |  | x | x |
| Income, job |  |  |  |
| Household amenities score |  |  |  |
| Marital status |  |  |  |
| Hours of sleep |  |  |  |
| Mental status |  |  |  |
| Height |  |  |  |
| Shared frailty |  |  |  |
| Family history of cancer |  |  |  |
| Family history of hypertension |  |  |  |
| History of hypercholesterolemia or hypertension |  |  |  |
| Menopausal status |  | x |  |
| Postmenopausal hormone use |  |  |  |
| Aspirin, analgesic use |  |  |  |
| Opium use |  |  |  |
| Oral contraceptive use |  |  |  |
| C-reactive protein |  |  |  |
| Carbohydrates |  |  |  |
| Grains |  |  | x |
| Fruits, vegetables |  | x |  |
| Fiber |  |  |  |
| Meat, poultry, fish |  | x | x |
| Egg |  |  |  |
| Dairy |  |  |  |
| Nuts |  |  |  |
| Other protein sources |  |  |  |
| No legume consumption |  |  |  |
| Fat |  |  |  |
| PUFA |  |  |  |
| MUFA |  |  |  |
| SFA |  |  |  |
| Trans fat |  |  |  |
| MUFA:SFA |  |  |  |
| Dietary cholesterol |  |  |  |
| Dietary methionine |  |  |  |
| Vitamin supplements use |  |  |  |
| Magnesium |  |  |  |
| Potassium |  |  |  |
| Sodium |  | x |  |
| Alcohol |  | x |  |
| Tea, coffee |  |  |  |
| Water |  |  |  |
| SSBs, ASBs, fruit juice |  |  |  |
| Junk food (chips, pizza, desserts) |  |  |  |
| Legume-adjusted dietary score |  |  |  |
| MedDiet adherence |  |  |  |
| Healthy Food Score |  |  |  |
| Centre |  | x | x |
| Birth cohort, cohort |  |  |  |
| Time period, season |  |  |  |
| Area of recruitment, residency |  |  | x |
| Method version |  |  |  |
| Intervention group |  |  |  |
| Randomization treatment assignment |  |  |  |

AHS=Adventists Health Study; ASB=artificially sweetened beverage; JPHC=Japan Public Health Center; MI=myocardial infarction; PURE= Prospective Urban Rural Epidemiology; SSB=sugar sweetened beverage

^1^ Includes adjustment for medication use for respective condition or levels of relevant biomarkers

## Supplemental Table 20 – Analysis of confounding variables among prospective cohort studies assessing the association between dietary pulses with or without other legumes and stroke incidence

| **COHORT** | **JPHC** | **Finnish Mobile Clinic Health Examination Survey** | **HPFS** | **NHS** | **EPIC-Greece** | **ARIC** | **PURE** |
| --- | --- | --- | --- | --- | --- | --- | --- |
| **STUDY (Supplemental reference)** | **Kokubo et al. 2007 (10)** | **Mizrahi et al. 2009 (37)** | **Bernstein et al. 2012 (38)** | **Bernstein et al. 2012 (38)** | **Misirli et al. 2012 (39)** | **Haring et al. 2015 (40)** | **Miller et al. 2017 (11)** |
| **Pre-specified primary confounding variables** |  |  |  |  |  |  |  |
| Age | x | x | x | x | x | x | x |
| **Pre-specified secondary confounding variables** |  |  |  |  |  |  |  |
| Sex | x | x | x | x | x | x | x |
| Family history of CVD |  |  | x | x |  |  |  |
| Smoking | x | x | x | x | x | x | x |
| Markers of overweight/obesity  (BMI, weight, waist circumference, waist-to-hip ratio) | x | x | x | x | x | x | x |
| Diabetes ^1^ | x |  |  |  | x |  | x |
| Hypertension ^1^ | x | x |  |  | x | x | x |
| Dyslipidemia ^1^ | x | x |  |  |  | x | x |
| Energy intake | x | x | x | x | x | x | x |
| Physical activity | x | x | x | x | x | x | x |
| **Other confounding variables** |  |  |  |  |  |  |  |
| Ethnicity, race, country of birth |  |  |  |  |  | x |  |
| Education | x |  |  |  | x | x | x |
| Income, job |  |  |  |  |  |  |  |
| Household amenities score |  |  |  |  |  |  |  |
| Marital status |  |  |  |  |  |  |  |
| Hours of sleep |  |  |  |  |  |  |  |
| Mental status |  |  |  |  |  |  |  |
| Height |  |  |  |  |  |  |  |
| Shared frailty |  |  |  |  |  |  |  |
| Family history of cancer |  |  |  |  |  |  |  |
| Family history of hypertension |  |  |  |  |  |  |  |
| History of hypercholesterolemia or hypertension |  |  |  |  |  |  |  |
| Menopausal status | x |  |  | x |  |  |  |
| Postmenopausal hormone use |  |  |  | x |  |  |  |
| Aspirin, analgesic use |  |  | x | x |  |  |  |
| Opium use |  |  |  |  |  |  |  |
| Oral contraceptive use |  |  |  |  |  |  |  |
| C-reactive protein |  |  |  |  |  |  |  |
| Carbohydrates |  |  |  |  |  | x |  |
| Grains |  |  |  |  |  |  | x |
| Fruits, vegetables | x |  | x | x |  |  |  |
| Fiber |  |  | x | x |  | x |  |
| Meat, poultry, fish | x |  |  |  |  |  | x |
| Egg |  |  |  |  |  |  |  |
| Dairy |  |  |  |  |  |  |  |
| Nuts |  |  |  |  |  |  |  |
| Other protein sources |  |  | x | x |  |  |  |
| No legume consumption |  |  |  |  |  |  |  |
| Fat |  |  |  |  |  | x |  |
| PUFA |  |  |  |  |  |  |  |
| MUFA |  |  |  |  |  |  |  |
| SFA |  |  |  |  |  |  |  |
| Trans fat |  |  | x | x |  |  |  |
| MUFA:SFA |  |  |  |  |  |  |  |
| Dietary cholesterol |  |  |  |  |  |  |  |
| Dietary methionine |  |  |  |  |  |  |  |
| Vitamin supplements use |  |  | x | x |  |  |  |
| Magnesium |  |  |  |  |  | x |  |
| Potassium |  |  |  |  |  |  |  |
| Sodium | x |  |  |  |  |  |  |
| Alcohol | x |  | x | x |  | x |  |
| Tea, coffee |  |  |  |  |  |  |  |
| Water |  |  |  |  |  |  |  |
| SSBs, ASBs, fruit juice |  |  |  |  |  |  |  |
| Junk food (chips, pizza, desserts) |  |  |  |  |  |  |  |
| Legume-adjusted dietary score |  |  |  |  |  |  |  |
| MedDiet adherence |  |  |  |  |  |  |  |
| Healthy Food Score |  |  |  |  |  |  |  |
| Centre | x |  |  |  |  | x | x |
| Birth cohort, cohort |  |  |  |  |  |  |  |
| Time period, season |  |  | x | x |  |  |  |
| Area of recruitment, residency |  |  |  |  |  |  | x |
| Method version |  |  |  |  |  |  |  |
| Intervention group |  |  |  |  |  |  |  |
| Randomization treatment assignment |  |  |  |  |  |  |  |

ASB=artificially sweetened beverage; ARIC=Atherosclerosis Risk in Communities; EPIC=European Prospective Investigation into Cancer and Nutrition; HPFS=Health Professionals Follow-Up Study; JPHC=Japan Public Health Center; NHS=Nurses’ Health Study; PURE=Prospective Urban Rural Epidemiology; SSB=sugar sweetened beverage

^1^ Includes adjustment for medication use for respective condition or levels of relevant biomarkers

## Supplemental Table 21 – Analysis of confounding variables among prospective cohort studies assessing the association between dietary pulses with or without other legumes and stroke mortality

| **COHORT** | **JACC** | **EPIC-Greece** | **LINXIAN NIT** | **Moli-sani** | **Golestan Cohort Study** | **HAPIEE Study** |
| --- | --- | --- | --- | --- | --- | --- |
| **STUDY (Supplemental reference)** | **Nagura et al. 2009 (19)** | **Misirli et al. 2012 (39)** | **Wang et al. 2016 (36)** | **Bonaccio et al. 2017 (23)** | **Farvid et al. 2017 (24)** | **Stefler et al. 2017 (25)** |
| **Pre-specified primary confounding variables** |  |  |  |  |  |  |
| Age | x | x | x | x | x | x |
| **Pre-specified secondary confounding variables** |  |  |  |  |  |  |
| Sex | x | x | x | x | x | x |
| Family history of CVD |  |  |  |  |  |  |
| Smoking | x | x | x | x | x | x |
| Markers of overweight/obesity  (BMI, weight, waist circumference, waist-to-hip ratio) | x | x | x |  | x |  |
| Diabetes ^1^ | x | x |  | x |  |  |
| Hypertension ^1^ | x | x |  |  | x |  |
| Dyslipidemia ^1^ |  |  |  |  |  |  |
| Energy intake |  | x |  | x | x | x |
| Physical activity | x | x |  | x | x | x |
| **Other confounding variables** |  |  |  |  |  |  |
| Ethnicity, race, country of birth |  |  |  |  | x |  |
| Education | x | x |  | x | x | x |
| Income, job |  |  |  | x | x |  |
| Household amenities score |  |  |  |  |  | x |
| Marital status |  |  |  |  | x | x |
| Hours of sleep | x |  |  |  |  |  |
| Mental status | x |  |  |  |  |  |
| Height |  |  |  |  |  |  |
| Shared frailty |  |  |  |  |  |  |
| Family history of cancer |  |  |  |  | x |  |
| Family history of hypertension |  |  |  |  |  |  |
| History of hypercholesterolemia or hypertension |  |  |  |  |  |  |
| Menopausal status |  |  |  |  |  |  |
| Postmenopausal hormone use |  |  |  |  |  |  |
| Aspirin, analgesic use |  |  |  |  | x |  |
| Opium use |  |  |  |  | x |  |
| Oral contraceptive use |  |  |  |  |  |  |
| C-reactive protein |  |  |  | x |  |  |
| Carbohydrates |  |  |  |  |  |  |
| Grains |  |  |  |  |  |  |
| Fruits, vegetables | x |  |  |  |  |  |
| Fiber |  |  |  |  |  |  |
| Meat, poultry, fish |  |  |  |  |  |  |
| Egg |  |  |  |  |  |  |
| Dairy |  |  |  |  |  |  |
| Nuts |  |  |  |  |  |  |
| Other protein sources |  |  |  |  |  |  |
| No legume consumption |  |  |  |  |  |  |
| Fat |  |  |  |  |  |  |
| PUFA | x |  |  |  |  |  |
| MUFA |  |  |  |  |  |  |
| SFA | x |  |  |  |  |  |
| Trans fat |  |  |  |  |  |  |
| MUFA:SFA |  |  |  |  |  |  |
| Dietary cholesterol | x |  |  |  |  |  |
| Dietary methionine |  |  |  |  |  |  |
| Vitamin supplements use |  |  |  |  |  | x |
| Magnesium |  |  |  |  |  |  |
| Potassium |  |  |  |  |  |  |
| Sodium | x |  |  |  |  |  |
| Alcohol | x |  | x |  | x |  |
| Tea, coffee |  |  |  | x |  |  |
| Water |  |  |  |  |  |  |
| SSBs, ASBs, fruit juice |  |  |  |  |  |  |
| Junk food (chips, pizza, desserts) |  |  |  |  |  |  |
| Legume-adjusted dietary score |  |  |  | x |  |  |
| MedDiet adherence |  |  |  |  |  |  |
| Healthy Food Score |  |  |  |  |  |  |
| Centre |  |  |  |  |  |  |
| Birth cohort, cohort |  |  |  |  |  | x |
| Time period, season |  |  | x | x |  |  |
| Area of recruitment, residency |  |  | x |  | x |  |
| Method version |  |  |  |  |  |  |
| Intervention group |  |  |  |  |  |  |
| Randomization treatment assignment |  |  |  |  |  |  |

ASB=artificially sweetened beverage; EPIC=European Prospective Investigation into Cancer and Nutrition; HAPIEE=Health Alcohol and Psychosocial factors in Eastern Europe Study; JACC=Japan Collaborative Cohort; LINXIAN NIT=LINXIAN Nutrition Intervention Trials; SSB=sugar sweetened beverage

^1^ Includes adjustment for medication use for respective condition or levels of relevant biomarkers

## Supplemental Table 22 – Analysis of confounding variables among prospective cohort studies assessing the association between dietary pulses with or without other legumes and diabetes incidence

| **COHORT** | **IWHS** | **MCCS** | **NHS** | **SWHS** | **MDC** | **EPIC-Potsdam** | **PREDIMED** | **TLGS** |
| --- | --- | --- | --- | --- | --- | --- | --- | --- |
| **STUDY (Supplemental reference)** | **Meyer et al. 2000 (41)** | **Hodge et al. 2004 (42)** | **Bazzano et al. 2008 (14)** | **Villegas et al. 2008 (44)** | **Ericson et al. 2013 (45)** | **von Ruesten et al. 2013 (15)** | **Becerra-Tomas et al. 2017 (46)** | **Khalili-Moghadam et al. 2019 (47)** |
| **Pre-specified primary confounding variables** |  |  |  |  |  |  |  |  |
| Age | x | x | x | x | x | x | x |  |
| **Pre-specified secondary confounding variables** |  |  |  |  |  |  |  |  |
| Sex | x | x | x | x | x | x | x |  |
| Family history of diabetes |  | x |  |  |  |  |  |  |
| Smoking | x |  | x | x | x | x | x |  |
| Markers of overweight/obesity  (BMI, weight, waist circumference, waist-to-hip ratio) | x | x | x | x | x | x | x |  |
| Energy intake | x | x | x | x | x | x |  |  |
| Physical activity | x | x | x | x | x | x | x |  |
| **Other confounding variables** |  |  |  |  |  |  |  |  |
| Ethnicity, race, country of birth |  | x |  |  |  |  |  |  |
| Education | x | x |  | x | x | x | x |  |
| Income, job |  |  |  | x |  |  |  |  |
| Household amenities score |  |  |  |  |  |  |  |  |
| Marital status |  |  |  |  |  |  |  |  |
| Hours of sleep |  |  |  |  |  |  |  |  |
| Mental status |  |  |  |  |  |  |  |  |
| Height |  |  |  |  |  |  |  |  |
| Shared frailty |  |  |  |  |  |  |  |  |
| Family history of cancer |  |  |  |  |  |  |  |  |
| Family history of hypertension |  |  |  |  |  |  |  |  |
| History of hypercholesterolemia or hypertension |  |  |  |  |  |  |  |  |
| Menopausal status |  |  |  |  |  |  |  |  |
| Diabetes Risk Score ^1^ |  |  |  |  |  |  |  | x |
| Postmenopausal hormone use |  |  | x |  |  |  |  |  |
| Aspirin, analgesic use |  |  |  |  |  |  |  |  |
| Opium use |  |  |  |  |  |  |  |  |
| Oral contraceptive use |  |  |  |  |  |  |  |  |
| C-reactive protein |  |  |  |  |  |  |  |  |
| Carbohydrates |  |  |  |  |  |  |  |  |
| Grains |  |  | x |  |  | x |  |  |
| Fruits, vegetables |  |  | x | x |  | x |  |  |
| Fiber |  |  |  | x |  |  |  |  |
| Meat, poultry, fish |  |  | x |  |  | x |  |  |
| Egg |  |  |  |  |  | x |  |  |
| Dairy |  |  |  |  |  | x |  |  |
| Nuts |  |  | x |  |  | x |  |  |
| Other protein sources |  |  |  |  |  |  |  |  |
| No legume consumption |  |  |  |  |  |  |  |  |
| Fat |  |  |  |  |  | x |  |  |
| PUFA |  |  |  |  |  |  |  |  |
| MUFA |  |  |  |  |  |  |  |  |
| SFA |  |  |  |  |  |  |  |  |
| Trans fat |  |  |  |  |  |  |  |  |
| MUFA:SFA |  |  |  |  |  |  |  |  |
| Dietary cholesterol |  |  |  |  |  |  |  |  |
| Dietary methionine |  |  |  |  |  |  |  |  |
| Vitamin supplements use |  |  |  |  |  | x |  |  |
| Magnesium |  |  |  |  |  |  |  |  |
| Potassium |  |  |  |  |  |  |  |  |
| Alcohol | x | x | x | x | x | x | x |  |
| Tea, coffee |  |  | x |  |  | x |  |  |
| Water |  |  |  |  |  | x |  |  |
| SSBs, ASBs, fruit juice |  |  | x |  |  | x |  |  |
| Junk food (chips, pizza, desserts) |  |  |  |  |  | x |  |  |
| Legume-adjusted dietary score |  |  |  |  |  |  | x |  |
| MedDiet adherence |  |  |  |  |  |  | x |  |
| Healthy Food Score |  |  |  |  |  |  |  |  |
| Centre |  |  |  |  |  |  |  |  |
| Birth cohort, cohort |  |  |  |  |  |  |  |  |
| Time period, season |  |  |  |  | x |  |  |  |
| Area of recruitment, residency |  |  |  |  |  |  |  |  |
| Method version |  |  |  |  | x |  |  |  |
| Intervention group |  |  |  |  |  |  | x |  |
| Randomization treatment assignment |  |  |  |  |  |  |  |  |

ASB=artificially sweetened beverage; EPIC=European Prospective Investigation into Cancer and Nutrition; IWHS=Iowa Women's Health Study; MCCS=Melbourne Collaborative Cohort Study; MDC=Malmo Diet and Cancer; NHS=Nurses’ Health Study; PREDIMED=PREvencion con DIeta MEDiterranea study; SWHS=Shanghai Women's Health Study; SSB=sugar sweetened beverage; TLGS= Tehran Lipid and Glucose Study

^1^ Khalili-Moghadam et al. 2019 (48) defined diabetes risk score as follows: 5 points for having a family history of diabetes; fasting plasma glucose concentrations divided into <5, 5−5.5, and 5.6−6.9 mmol/L with 0, 12, and 33 points, respectively; systolic blood pressure divided into three categories: <120, 120−140, and ≥140 mm Hg with 0, 3, and 7 points, respectively; waist to height ratio divided into three categories: <0.54, 0.54−0.59, and ≥0.59 with 0, 6, and 11 points, respectively; TG/HDL-C (≥3.5) (3 points), and TG/HDL-C (<3.5) (0 point).

## Supplemental Table 23 – Analysis of confounding variables among prospective cohort studies assessing the association between dietary pulses with or without other legumes and hypertension incidence

| **COHORT** | **SUN** | **ARIC** | **NHS** | **NHS II** | **HPFS** | **TLGS** | **NutriNet-Santé** |
| --- | --- | --- | --- | --- | --- | --- | --- |
| **STUDY (Supplemental reference)** | **Nunez-Cordoba et al. 2009 (48)** | **Weng et al. 2013 (49)** | **Borgi et al. 2016 (50)** | **Borgi et al. 2016 (50)** | **Borgi et al. 2016 (50)** | **Golzarand et al. 2016 (51)** | **Lelong et al. 2017 (52)** |
| **Pre-specified primary confounding variables** |  |  |  |  |  |  |  |
| Age | x | x | x | x | x | x | x |
| **Pre-specified secondary confounding variables** |  |  |  |  |  |  |  |
| Sex | x | x | x | x | x | x | x |
| Diabetes |  |  |  |  |  |  |  |
| Smoking | x | x | x | x | x | x | x |
| Markers of overweight/obesity  (BMI, weight, waist circumference, waist-to-hip ratio) | x |  | x | x | x | x | x |
| Energy intake | x | x | x | x | x | x | x |
| Sodium intake |  |  |  |  |  | x |  |
| Physical activity | x | x | x | x | x | x | x |
| **Other confounding variables** |  |  |  |  |  |  |  |
| Ethnicity, race, country of birth |  | x | x | x | x |  |  |
| Education |  | x |  |  |  |  | x |
| Income, job |  |  |  |  |  |  |  |
| Household amenities score |  |  |  |  |  |  |  |
| Marital status |  |  |  |  |  |  |  |
| Hours of sleep |  |  |  |  |  |  |  |
| Mental status |  |  |  |  |  |  |  |
| Height |  |  |  |  |  |  |  |
| Shared frailty |  |  |  |  |  |  |  |
| Family history of cancer |  |  |  |  |  |  |  |
| Family history of hypertension | x |  | x | x | x |  | x |
| History of hypercholesterolemia or hypertension |  |  |  |  |  |  |  |
| Menopausal status |  |  | x | x |  |  |  |
| Postmenopausal hormone use |  |  |  |  |  |  |  |
| Aspirin, analgesic use |  |  | x | x | x |  |  |
| Opium use |  |  |  |  |  |  |  |
| Oral contraceptive use |  |  | x | x |  |  |  |
| C-reactive protein |  |  |  |  |  |  |  |
| Carbohydrates |  |  |  |  |  |  |  |
| Grains | x |  | x | x | x |  |  |
| Fruits, vegetables | x |  |  |  |  |  |  |
| Fiber |  |  |  |  |  | x |  |
| Meat, poultry, fish | x |  | x | x | x |  |  |
| Egg |  |  |  |  |  |  |  |
| Dairy | x |  |  |  |  |  |  |
| Nuts | x |  |  |  |  |  |  |
| Other protein sources |  |  |  |  |  |  |  |
| No legume consumption |  |  |  |  |  |  |  |
| Fat |  |  |  |  |  |  |  |
| PUFA |  |  |  |  |  |  |  |
| MUFA |  |  |  |  |  |  |  |
| SFA |  |  |  |  |  |  |  |
| Trans fat |  |  |  |  |  |  |  |
| MUFA:SFA | x |  |  |  |  |  |  |
| Dietary cholesterol |  |  |  |  |  |  |  |
| Dietary methionine |  |  |  |  |  |  |  |
| Vitamin supplements use |  |  |  |  |  |  |  |
| Magnesium |  |  |  |  |  |  |  |
| Potassium |  |  |  |  |  | x |  |
| Alcohol | x |  | x | x | x |  | x |
| Tea, coffee | x |  |  |  |  |  |  |
| Water |  |  |  |  |  |  |  |
| SSBs, ASBs, fruit juice |  |  | x | x | x |  |  |
| Junk food (chips, pizza, desserts) |  |  |  |  |  |  |  |
| Legume-adjusted dietary score |  |  |  |  |  |  |  |
| MedDiet adherence |  |  |  |  |  |  |  |
| Healthy Food Score |  | x |  |  |  |  |  |
| Centre |  | x |  |  |  |  |  |
| Birth cohort, cohort |  |  |  |  |  |  |  |
| Time period, season |  |  |  |  |  |  |  |
| Area of recruitment, residency |  |  |  |  |  |  |  |
| Method version |  |  |  |  |  |  |  |
| Intervention group |  |  |  |  |  |  |  |
| Randomization treatment assignment |  |  |  |  |  |  |  |

ARIC=Atherosclerosis Risk in Communities; HPFS=Health Professionals Follow-Up Study; NHS=Nurses’ Health Study; SUN=Seguimiento Universidad de Navarra; TLGS=Tehran Lipid and Glucose Study

## Supplemental Table 24 – Analysis of confounding variables among prospective cohort studies assessing the association between dietary pulses with or without other legumes and obesity incidence

| **COHORT** | **WHS** |
| --- | --- |
| **STUDY (Supplemental reference)** | **Rautiainen et al. 2015 (53)** |
| **Pre-specified primary confounding variables** |  |
| Age | x |
| Sex | x |
| Smoking | x |
| Markers of overweight/obesity  (BMI, weight, waist circumference, waist-to-hip ratio) | x |
| Energy intake | x |
| Physical activity | x |
| **Other confounding variables** |  |
| Ethnicity, race, country of birth |  |
| Education |  |
| Income, job |  |
| Household amenities score |  |
| Marital status |  |
| Hours of sleep |  |
| Mental status |  |
| Height |  |
| Shared frailty |  |
| Family history of diabetes |  |
| Family history of cancer |  |
| Family history of hypertension |  |
| History of hypercholesterolemia or hypertension | x |
| Menopausal status | x |
| Postmenopausal hormone use | x |
| Aspirin, analgesic use |  |
| Opium use |  |
| Oral contraceptive use |  |
| C-reactive protein |  |
| Carbohydrates |  |
| Grains |  |
| Fruits, vegetables |  |
| Fiber |  |
| Meat, poultry, fish |  |
| Egg |  |
| Dairy |  |
| Nuts |  |
| Other protein sources |  |
| No legume consumption |  |
| Fat |  |
| PUFA |  |
| MUFA |  |
| SFA |  |
| Trans fat |  |
| MUFA:SFA |  |
| Dietary cholesterol |  |
| Dietary methionine |  |
| Vitamin supplements use | x |
| Magnesium |  |
| Potassium |  |
| Alcohol | x |
| Tea, coffee |  |
| Water |  |
| SSBs, ASBs, fruit juice |  |
| Junk food (chips, pizza, desserts) |  |
| Legume-adjusted dietary score |  |
| MedDiet adherence |  |
| Healthy Food Score |  |
| Centre |  |
| Birth cohort, cohort |  |
| Time period, season |  |
| Area of recruitment, residency |  |
| Method version |  |
| Intervention group |  |
| Randomization treatment assignment | x |

WHS=Women’s Health Study

## Supplemental Table 25 – Newcastle-Ottawa Scale (NOS) for prospective cohort studies

| **Study**  **(Supplemental reference)** | **Selection (maximum 4)** | | | | **Outcome (maximum 3)** | | | **Comparability (maximum 2)** | | **Total** |
| --- | --- | --- | --- | --- | --- | --- | --- | --- | --- | --- |
|  | **Representativeness of the exposed cohort** | **Selection of the non-exposed cohort** | **Ascertainment of exposure** | **Outcome of interest not present at start of study** | **Assessment of outcome** | **Sufficient follow-up duration** | **Adequate follow-up** | **Study controls for primary confounding variable ^1^** | **Study controls for secondary confounding variables ^2^** |  |
| **Cardiovascular disease outcomes** | | | | | | | | | | |
| Fraser et al. 1992 (9) | 0 | 1 | 0 | 1 | 1 | 1 | 1 | 1 | 0 | 6 |
| Bazzano et al. 2001 (14) | 1 | 1 | 1 | 1 | 1 | 1 | 1 | 1 | 1 | 9 |
| Kelemen et al. 2005 (35) | 0 | 1 | 0 | 1 | 1 | 1 | 1 | 1 | 0 | 6 |
| Kokubo et al. 2007 (10) | 1 | 1 | 0 | 1 | 1 | 1 | 1 | 1 | 1 | 8 |
| Buckland et al. 2009 (27) | 1 | 1 | 1 | 1 | 1 | 1 | 1 | 1 | 1 | 9 |
| Mizrahi et al. 2009 (37) | 1 | 1 | 1 | 1 | 1 | 1 | 1 | 1 | 1 | 9 |
| Nagura et al. 2009 (19) | 1 | 1 | 0 | 1 | 1 | 1 | 0 | 1 | 0 | 6 |
| Bernstein et al. 2010 (28) | 0 | 1 | 0 | 1 | 1 | 1 | 1 | 1 | 0 | 6 |
| Gardener et al. 2011 (20) | 1 | 1 | 1 | 1 | 1 | 1 | 1 | 1 | 0 | 8 |
| Martinez-Gonzalez et al. 2011 (29) | 0 | 1 | 0 | 1 | 1 | 1 | 1 | 1 | 1 | 7 |
| Bernstein et al. 2012 – HPFS (38) | 0 | 1 | 0 | 1 | 1 | 1 | 1 | 1 | 0 | 6 |
| Bernstein et al. 2012 – NHS (38) | 0 | 1 | 0 | 1 | 1 | 1 | 1 | 1 | 0 | 6 |
| Dilis et al. 2012 (30) | 1 | 1 | 1 | 1 | 1 | 1 | 1 | 1 | 0 | 8 |
| Misirli et al. 2012 (39) | 1 | 1 | 1 | 1 | 1 | 1 | 1 | 1 | 1 | 9 |
| von Ruesten et al. 2013 (15) | 1 | 1 | 0 | 1 | 1 | 1 | 1 | 1 | 1 | 8 |
| Haring et al. 2014 (32) | 1 | 1 | 1 | 1 | 1 | 1 | 1 | 1 | 1 | 9 |
| Russell et al. 2014 (12) | 1 | 1 | 0 | 1 | 1 | 1 | 0 | 1 | 0 | 6 |
| Yu et al. 2014 (34) | 1 | 1 | 0 | 1 | 1 | 1 | 1 | 1 | 1 | 8 |
| Haring et al. 2015 (40) | 1 | 1 | 1 | 1 | 1 | 1 | 1 | 1 | 1 | 9 |
| Lassale et al. 2015 (22) | 1 | 1 | 0 | 1 | 1 | 1 | 1 | 1 | 0 | 7 |
| Nouri et al. 2016 (16) | 1 | 1 | 0 | 1 | 1 | 1 | 1 | 1 | 0 | 7 |
| Wang et al. 2016 (36) | 0 | 1 | 0 | 1 | 1 | 1 | 0 | 1 | 0 | 5 |
| Bonaccio et al. 2017 (23) | 1 | 1 | 1 | 1 | 1 | 1 | 1 | 1 | 0 | 8 |
| Buil-Cosailes et al. 2017 (17) | 0 | 1 | 0 | 1 | 1 | 1 | 1 | 1 | 1 | 7 |
| Farvid et al. 2017 (24) | 1 | 1 | 0 | 1 | 1 | 1 | 1 | 1 | 0 | 7 |
| Miller et al. 2017 (11) | 1 | 1 | 0 | 1 | 1 | 1 | 1 | 1 | 1 | 8 |
| Stefler et al. 2017 (25) | 1 | 1 | 0 | 1 | 1 | 1 | 0 | 1 | 0 | 6 |
| Papandreou et al. 2018 (26) | 1 | 1 | 1 | 1 | 1 | 1 | 1 | 1 | 1 | 9 |
| van den Brandt et al. 2019 (13) | 1 | 1 | 0 | 1 | 1 | 1 | 0 | 1 | 1 | 7 |
| **Diabetes outcomes** | | | | | | | | | | |
| Meyer et al. 2000 (41) | 0 | 1 | 0 | 1 | 0 | 1 | 1 | 1 | 1 | 6 |
| Hodge et al. 2004 (42) | 1 | 1 | 0 | 1 | 1 | 1 | 1 | 1 | 1 | 8 |
| Bazzano et al. 2008 (43) | 0 | 1 | 0 | 1 | 0 | 1 | 1 | 1 | 1 | 6 |
| Villegas et al. 2008 (44) | 0 | 1 | 1 | 1 | 0 | 1 | 1 | 1 | 1 | 7 |
| Ericson et al. 2013 (45) | 1 | 1 | 1 | 1 | 1 | 1 | 0 | 1 | 1 | 8 |
| von Ruesten et al. 2013 (15) | 1 | 1 | 0 | 1 | 1 | 1 | 1 | 1 | 1 | 8 |
| Becerra-Tomas et al. 2017 (46) | 1 | 1 | 1 | 1 | 1 | 1 | 1 | 1 | 1 | 9 |
| Khalili-Moghadam et al. 2019 (47) | 1 | 1 | 1 | 1 | 0 | 1 | 1 | 0 | 0 | 6 |
| **Hypertension outcomes** | | | | | | | | | | |
| Nunez-Cordoba et al. 2009 (48) | 0 | 1 | 0 | 1 | 0 | 1 | 1 | 1 | 1 | 6 |
| Weng et al. 2013 (49) | 1 | 1 | 1 | 1 | 1 | 1 | 1 | 1 | 0 | 8 |
| Borgi et al. 2016 – NHS (50) | 0 | 1 | 0 | 1 | 0 | 1 | 0 | 1 | 1 | 5 |
| Borgi et al. 2016 – NHS II (50) | 0 | 1 | 0 | 1 | 0 | 1 | 0 | 1 | 1 | 5 |
| Borgi et al. 2016 – HPFS (50) | 0 | 1 | 0 | 1 | 0 | 1 | 0 | 1 | 1 | 5 |
| Golzarand et al. 2016 (51) | 1 | 1 | 1 | 1 | 1 | 1 | 1 | 1 | 1 | 9 |
| Lelong et al. 2017 (52) | 0 | 1 | 0 | `1 | 0 | 1 | 1 | 1 | 1 | 6 |
| **Obesity outcomes** | | | | | | | | | | |
| Rautiainen et al. 2015 (53) | 0 | 1 | 0 | 1 | 0 | 1 | 1 | 1 | 1 | 6 |

^1^ Primary confounding variable was age for all outcomes, with the exception of obesity incidence, which awarded 1/3 point each for controlling for the following confounding variables: age, physical activity, energy intake, sex, baseline BMI or body weight, and smoking status.

^2^ One point was awarded for controlling for 7 of the 9 secondary confounding variables for cardiovascular disease outcomes (sex, family history of CVD, smoking, markers of overweight/obesity, diabetes, hypertension, dyslipidemia, energy intake, and physical activity), 4 of the 6 secondary confounding variables for diabetes outcomes (sex, family history of diabetes, smoking, markers of overweight/obesity, energy intake and physical activity) and for 5 of the 7 secondary confounding variables for hypertension outcomes (sex, diabetes, smoking, markers of overweight/obesity, energy intake, sodium intake and physical activity).

## Supplemental Table 26 – Select sensitivity analyses in which the systematic removal of an individual study altered the significance of the pooled effect estimate or the evidence for heterogeneity

| **Removal of** | **RR [95% CI], *P-value*** | **Heterogeneity** |
| --- | --- | --- |
| **CVD Incidence** | **0.92 [0.85, 0.99], *P*=0.03** | ***I²* = 19%, *P* = 0.29** |
| Bazzano et al. 2001 (14) | 0.91 [0.81, 1.03], *P*=0.12 | *I²* = 32%, *P* = 0.20 |
| Kokubo et al. 2007 – W (10) | 0.93 [0.86, 1.00], *P*=0.05 | *I²* = 16%, *P* = 0.31 |
| Nouri et al. 2016 (16) | 0.93 [0.85, 1.01], *P*=0.08 | *I²* = 24%, *P* = 0.25 |
| Buil-Cosailes et al. 2017 (17) | 0.91 [0.84, 1.00], *P*=0.05 | *I²* = 32%, *P* = 0.20 |
| Miller et al. 2017 (11) | 0.94 [0.88, 1.01], *P*=0.09 | *I²* = 0%, *P* = 0.42 |
| **CVD Mortality** | **0.97 [0.89, 1.06], *P*=0.53** | ***I²* = 47%, *P* = 0.04** |
| Kokubo et al. 2007 – W (10) | 0.97 [0.88, 1.06], *P*=0.51 | *I²* = 51%, *P* = 0.03 |
| Lassale et al. 2015 (22) | 0.98 [0.87, 1.09], *P*=0.65 | *I²* = 51%, *P* = 0.03 |
| Miller et al. 2017 (11) | 0.98 [0.89, 1.08], *P*=0.69 | *I²* = 51%, *P* = 0.03 |
| Stefler et al. 2017 (25) | 0.98 [0.89, 1.08], *P*=0.67 | *I²* = 51%, *P* = 0.02 |
| van den Brandt et al. 2019 (13) | 0.97 [0.88, 1.07], *P*=0.58 | *I²* = 52%, *P* = 0.02 |
| **CHD Incidence** | **0.90 [0.83, 0.99], *P*=0.03** | ***I²* = 34%, *P* = 0.13** |
| Bazzano et al. 2001 (14) | 0.93 [0.86, 1.01], *P*=0.10 | *I²* = 14%; *P* = 0.32 |
| Bernstein et al. 2010 (28) | 0.91 [0.81, 1.02], *P*=0.10 | *I²* = 41%; *P* = 0.09 |
| Dilis et al. 2012 – W (30) | 0.91 [0.83, 1.00], *P*=0.05 | *I²* = 40%; *P* = 0.10 |
| **CHD Mortality** | **0.94 [0.82, 1.08], *P*=0.39** | ***I²* = 48%, *P* = 0.05** |
| Fraser et al. 1992 (9) | 0.92 [0.79, 1.08], *P*=0.32 | *I²* = 51%, *P* = 0.04 |
| Kelemen et al. 2005 (35) | 0.96 [0.82, 1.13], *P*=0.61 | *I²* = 51%, *P* = 0.05 |
| Nagura et al. 2009 (19) | 0.95 [0.81, 1.12], *P*=0.53 | *I²* = 54%, *P* = 0.03 |
| Dilis et al. 2012 – M (30) | 0.92 [0.79, 1.08], *P*=0.33 | *I²* = 52%, *P* = 0.04 |
| Dilis et al. 2012 – W (30) | 0.95 [0.82, 1.11], *P*=0.51 | *I²* = 53%, *P* = 0.04 |
| Stefler et al. 2017 (25) | 0.92 [0.79, 1.08], *P*=0.32 | *I²* = 52%, *P* = 0.04 |
| **Stroke Mortality** | **0.89 [0.78, 1.03], *P*=0.12** | ***I²* = 25%, *P* = 0.24** |
| Bonaccio et al. 2017 (23) | 0.88 [0.79, 0.99], *P*=0.03 | *I²* = 0%, *P* = 0.52 |

CVD=cardiovascular disease; CHD=coronary heart disease; HPFS=Health Professionals Follow-Up Study; M=men; RR=relative risk; W=women

## Supplemental Table 27 – Post-hoc sensitivity analyses assessing the association in studies reporting dietary pulses alone as the exposure*

| **Cardiometabolic**  **disease outcome** | **Included studies**  **(Supplemental reference)** | **RR [95% CI], *P-value*** | **Heterogeneity** |
| --- | --- | --- | --- |
| **CVD Incidence** | Kokubo et al. 2007 – M (10)  Kokubo et al. 2007 – W (10)  Buil-Cosailes et al. 2017 (17)  Miller et al. 2017 (11) | 0.88 [0.74, 1.05], *P*=0.14 | *I*² = 25%, *P* = 0.26 |
| **CHD Mortality** | Fraser et al. 1992 (9) | 1.06 [0.82, 1.37], *P*=0.64 | Not applicable |
| **MI Incidence** | Fraser et al. 1992 (9)  Kokubo et al. 2007 – M (10)  Kokubo et al. 2007 – W (10)  Miller et al. 2017 (11) | 0.90 [0.74, 1.10], *P*=0.29 | *I²* = 0%, *P* = 0.58 |
| **Stroke Incidence** | Kokubo et al. 2007 – M (10)  Kokubo et al. 2007 – W (10)  Miller et al. 2017 (11) | 0.92 [0.67, 1.27], *P*=0.61 | *I²* = 64%, *P* = 0.06 |
| **Stroke Mortality** | Wang et al. 2016 (36) | 0.84 [0.68, 1.05], *P*=0.12 | Not applicable |
| **Diabetes Incidence** | Meyer et al. 2000 (41) | 0.96 [0.76, 1.22], *P*=0.74 | Not applicable |
| **Hypertension Incidence** | Nunez-Cordoba et al. 2009 (48)  Borgi et al. 2016 – NHS (50)  Borgi et al. 2016 – NHS II (50)  Borgi et al. 2016 – HPFS (50)  Golzarand et al. 2016 (51) | 0.92 [0.87, 0.98], *P*=0.01 | *I²* = 0%, *P* = 0.55 |

CVD=cardiovascular disease; CHD=coronary heart disease; NHS=Nurses’ Health Study; HPFS=Health Professionals Follow-Up Study; M=men; MI=myocardial infarction; RR=relative risk; W=women

*Post-hoc sensitivity analyses were conducted for those outcomes that had <10 cohort comparison available. For outcomes with ≥10 cohort comparisons available, refer to the subgroup analyses reported in **Supplemental Figures 15-16.**

## Supplemental Table 28 – GRADE certainty of evidence assessment for the association between dietary pulses with or without other legumes and cardiometabolic disease outcomes

| **Certainty assessment** | | | | | | | | **RR (95% CI)** | **Certainty** |
| --- | --- | --- | --- | --- | --- | --- | --- | --- | --- |
| **Cardiometabolic**  **disease outcome** | **Cohort**  **comparisons,**  ***n*** | **Study design** | **Risk of bias** | **Inconsistency** | **Indirectness** | **Imprecision** | **Other**  **considerations** |  |  |
| **CVD incidence** | 7 | observational studies | not serious | not serious | not serious | serious ^1^ | dose response gradient ^2^ | **0.92 (0.85, 0.99)** | ⨁⨁◯◯ LOW |
| **CVD mortality** | 12 | observational studies | not serious | not serious | not serious | serious ^3^ | none | **0.97 (0.89, 1.06)** | ⨁◯◯◯ VERY LOW |
| **CHD incidence** | 10 | observational studies | not serious | not serious | not serious | serious ^4^ | none | **0.90 (0.83, 0.99)** | ⨁◯◯◯ VERY LOW |
| **CHD mortality** | 9 | observational studies | not serious | not serious | not serious | serious ^5^ | none | **0.94 (0.82, 1.08)** | ⨁◯◯◯ VERY LOW |
| **MI incidence** | 4 | observational studies | not serious | not serious | not serious | serious ^6^ | none | **0.90**  **(0.74, 1.10)** | ⨁◯◯◯ VERY LOW |
| **Stroke incidence** | 8 | observational studies | not serious | serious ^7^ | not serious | serious ^8^ | none | **0.98 (0.86, 1.11)** | ⨁◯◯◯ VERY LOW |
| **Stroke mortality** | 6 | observational studies | not serious | not serious | not serious | serious ^9^ | none | **0.89 (0.78, 1.03)** | ⨁◯◯◯ VERY LOW |
| **Diabetes incidence** | 9 | observational studies | not serious | serious ^10^ | not serious | serious ^11^ | none | **0.93**  **(0.83, 1.05)** | ⨁◯◯◯ VERY LOW |
| **Hypertension incidence** | 7 | observational studies | serious ^12^ | not serious | serious ^13^ | serious ^14^ | none | **0.91 (0.86, 0.97)** | ⨁◯◯◯ VERY LOW |
| **Obesity incidence** | 1 | observational studies | not serious | not serious ^15^ | serious ^16^ | not serious | none | **0.87**  **(0.81, 0.94)** | ⨁◯◯◯ VERY LOW |

CHD=chronic heart disease; CI=confidence interval; CVD=cardiovascular disease; MI=myocardial infarction; RR=risk ratio

^1^ Serious imprecision for CVD incidence, as the 95% CI (0.85, 0.99) overlapped with the minimally important difference for clinical benefit (RR 0.95).

^2^ Upgrade for a dose-response gradient, as the GLST dose-response analyses revealed a significant linear inverse relationship between dietary pulses with or without other legumes and CVD incidence (*P*=0.007, **Supplemental Figure 17a**).

^3^ Serious imprecision for CVD mortality, as the 95% CI (0.89, 1.06) overlapped with the minimally important difference for clinical benefit (RR 0.95) and harm (RR 1.05).

^4^ Serious imprecision for CHD incidence, as the 95% CI (0.83, 0.99) overlapped with the minimally important difference for clinical benefit (RR 0.95).

^5^ Serious imprecision for CHD mortality, as the 95% CI (0.82, 1.08) overlapped with the minimally important difference for clinical benefit (RR 0.95) and harm (RR 1.05).

^6^ Serious imprecision for MI incidence, as the 95% CI (0.74, 1.10) overlapped with the minimally important difference for clinical benefit (RR 0.95) and harm (RR 1.05).

^7^ Serious inconsistency for stroke incidence, as *I^2^*=58% and *P*=0.02.

^8^ Serious imprecision for stroke incidence, as the 95% CI (0.86, 1.11) overlapped with the minimally important difference for clinical benefit (RR 0.95) and harm (RR 1.05).

^9^ Serious imprecision for stroke mortality, as the 95% CI (0.78, 1.03) overlapped with the minimally important difference for clinical benefit (RR 0.95).

^10^ Serious inconsistency for diabetes incidence, as *I^2^*=70% and *P*=0.0008.

^11^ Serious imprecision for diabetes incidence, as the 95% CI (0.83, 1.05) overlapped with the minimally important difference for clinical benefit (RR 0.95).

^12^ Serious risk of bias for hypertension incidence, as >50% of the weight (68.7%) was contributed by studies considered to be high risk of bias (NOS<6).

^13^ Serious indirectness for hypertension incidence, as >50% of the weight (68.7%) was contributed by studies conducted in health professionals.

^14^ Serious imprecision for hypertension incidence, as the 95% CI (0.86, 0.97) overlapped with the minimally important difference for clinical benefit (RR 0.95).

^15^ Not able to assess inconsistency for obesity incidence as only 1 cohort study was available for inclusion.
^16^ Serious indirectness for obesity incidence, as only 1 cohort study was available for inclusion that was conducted in postmenopausal female health professionals.

# SUPPLEMENTAL REFERENCES

1. Bechthold A, Boeing H, Schwedhelm C, Hoffmann G, Knuppel S, Iqbal K, De Henauw S, Michels N, Devleesschauwer B, Schlesinger S, et al. Food groups and risk of coronary heart disease, stroke and heart failure: A systematic review and dose-response meta-analysis of prospective studies. Critical reviews in food science and nutrition 2019;59(7):1071-90. doi: 10.1080/10408398.2017.1392288.

2. Grosso G, Marventano S, Yang J, Micek A, Pajak A, Scalfi L, Galvano F, Kales SN. A comprehensive meta-analysis on evidence of Mediterranean diet and cardiovascular disease: Are individual components equal? Critical reviews in food science and nutrition 2017;57(15):3218-32. doi: 10.1080/10408398.2015.1107021.

3. Marventano S, Izquierdo Pulido M, Sanchez-Gonzalez C, Godos J, Speciani A, Galvano F, Grosso G. Legume consumption and CVD risk: a systematic review and meta-analysis. Public health nutrition 2017;20(2):245-54. doi: 10.1017/s1368980016002299.

4. Schwingshackl L, Hoffmann G, Lampousi AM, Knuppel S, Iqbal K, Schwedhelm C, Bechthold A, Schlesinger S, Boeing H. Food groups and risk of type 2 diabetes mellitus: a systematic review and meta-analysis of prospective studies. European journal of epidemiology 2017;32(5):363-75. doi: 10.1007/s10654-017-0246-y.

5. Schwingshackl L, Schwedhelm C, Hoffmann G, Knuppel S, Iqbal K, Andriolo V, Bechthold A, Schlesinger S, Boeing H. Food Groups and Risk of Hypertension: A Systematic Review and Dose-Response Meta-Analysis of Prospective Studies. Advances in nutrition (Bethesda, Md) 2017;8(6):793-803. doi: 10.3945/an.117.017178.

6. Schlesinger S, Neuenschwander M, Schwedhelm C, Hoffmann G, Bechthold A, Boeing H, Schwingshackl L. Food Groups and Risk of Overweight, Obesity, and Weight Gain: A Systematic Review and Dose-Response Meta-Analysis of Prospective Studies. Advances in nutrition (Bethesda, Md) 2019;10(2):205-18. doi: 10.1093/advances/nmy092.

7. Higgins JPT, Green S (editors). Cochrane Handbook for Systematic Reviews of Interventions Version 5.1.0 [updated March 2011]. The Cochrane Collaboration, 2011. Available from [www.cochrane-handbook.org](http://www.cochrane-handbook.org).

8. Guyatt GH, Oxman AD, Kunz R, Woodcock J, Brozek J, Helfand M, Alonso-Coello P, Glasziou P, Jaeschke R, Akl EA, et al. GRADE guidelines: 7. Rating the quality of evidence--inconsistency. Journal of clinical epidemiology 2011;64(12):1294-302. doi: 10.1016/j.jclinepi.2011.03.017.

9. Fraser GE, Sabate J, Beeson WL, Strahan TM. A possible protective effect of nut consumption on risk of coronary heart disease. The Adventist Health Study. Archives of internal medicine 1992;152(7):1416-24.

10. Kokubo Y, Iso H, Ishihara J, Okada K, Inoue M, Tsugane S. Association of dietary intake of soy, beans, and isoflavones with risk of cerebral and myocardial infarctions in Japanese populations: the Japan Public Health Center-based (JPHC) study cohort I. Circulation 2007;116(22):2553-62. doi: 10.1161/circulationaha.106.683755.

11. Miller V, Mente A, Dehghan M, Rangarajan S, Zhang X, Swaminathan S, Dagenais G, Gupta R, Mohan V, Lear S, et al. Fruit, vegetable, and legume intake, and cardiovascular disease and deaths in 18 countries (PURE): a prospective cohort study. Lancet (London, England) 2017;390(10107):2037-49. doi: 10.1016/s0140-6736(17)32253-5.

12. Russell J, Flood V. Regular consumption of legumes reduces the risk of cardiovascular mortality. Journal of Nutrition & Intermediary Metabolism 2014; 1:1-55.

13. van den Brandt PA. Red meat, processed meat, and other dietary protein sources and risk of overall and cause-specific mortality in The Netherlands Cohort Study. European journal of epidemiology 2019;34(4):351-69. doi: 10.1007/s10654-019-00483-9.

14. Bazzano LA, He J, Ogden LG, Loria C, Vupputuri S, Myers L, Whelton PK. Legume consumption and risk of coronary heart disease in US men and women: NHANES I Epidemiologic Follow-up Study. Archives of internal medicine 2001;161(21):2573-8.

15. von Ruesten A, Feller S, Bergmann MM, Boeing H. Diet and risk of chronic diseases: results from the first 8 years of follow-up in the EPIC-Potsdam study. European journal of clinical nutrition 2013;67(4):412-9. doi: 10.1038/ejcn.2013.7.

16. Nouri F, Sarrafzadegan N, Mohammadifard N, Sadeghi M, Mansourian M. Intake of legumes and the risk of cardiovascular disease: frailty modeling of a prospective cohort study in the Iranian middle-aged and older population. European journal of clinical nutrition 2016;70(2):217-21. doi: 10.1038/ejcn.2015.153.

17. Buil-Cosiales P, Martinez-Gonzalez MA, Ruiz-Canela M, Diez-Espino J, Garcia-Arellano A, Toledo E. Consumption of Fruit or Fiber-Fruit Decreases the Risk of Cardiovascular Disease in a Mediterranean Young Cohort. Nutrients 2017;9(3). doi: 10.3390/nu9030295.

18. de la Fuente-Arrillaga C, Ruiz ZV, Bes-Rastrollo M, Sampson L, Martinez-Gonzalez MA. Reproducibility of an FFQ validated in Spain. Public health nutrition 2010;13(9):1364-72. doi: 10.1017/s1368980009993065.

19. Nagura J, Iso H, Watanabe Y, Maruyama K, Date C, Toyoshima H, Yamamoto A, Kikuchi S, Koizumi A, Kondo T, et al. Fruit, vegetable and bean intake and mortality from cardiovascular disease among Japanese men and women: the JACC Study. The British journal of nutrition 2009;102(2):285-92. doi: 10.1017/s0007114508143586.

20. Gardener H, Wright CB, Gu Y, Demmer RT, Boden-Albala B, Elkind MS, Sacco RL, Scarmeas N. Mediterranean-style diet and risk of ischemic stroke, myocardial infarction, and vascular death: the Northern Manhattan Study. The American journal of clinical nutrition 2011;94(6):1458-64. doi: 10.3945/ajcn.111.012799.

21. Cancer Council Victoria. Dietary questionnaires, DQES v3.2. Cancer Council Victori, 2016. Available from <https://www.cancervic.org.au/downloads/cec/FFQs/DQES_v3.2_Sample-Questionnaire.pdf>.

22. Lassale C, Beulens J, Van Der Schouw Y, Roswall N, Weiderpass E, Romaguera D, Riboli E, Tzoulaki I. A pro-vegetarian food pattern and cardiovascular mortality in the epic study. Circulation 2015; Conference: American Heart Association's Epidemiology and Prevention/Lifestyle and Cardiometabolic Health, 131:A16.

23. Bonaccio M, Di Castelnuovo A, Costanzo S, Persichillo M, Donati MB, De Gaetano G, Iacoviello L. Higher adherence to the traditional Mediterranean diet is associated with lower cardiovascular risk and all-cause mortality in the elderly: Prospective findings from the Moli-sani study. European Journal of Preventive Cardiology 2017; 24 (1 Supplement 1): S8.

24. Farvid MS, Malekshah AF, Pourshams A, Poustchi H, Sepanlou SG, Sharafkhah M, Khoshnia M, Farvid M, Abnet CC, Kamangar F, et al. Dietary Protein Sources and All-Cause and Cause-Specific Mortality: The Golestan Cohort Study in Iran. American journal of preventive medicine 2017;52(2):237-48. doi: 10.1016/j.amepre.2016.10.041.

25. Stefler D, Malyutina S, Kubinova R, Pajak A, Peasey A, Pikhart H, Brunner EJ, Bobak M. Mediterranean diet score and total and cardiovascular mortality in Eastern Europe: the HAPIEE study. European journal of nutrition 2017;56(1):421-9. doi: 10.1007/s00394-015-1092-x.

26. Papandreou C, Becerra-Tomas N, Bullo M, Martinez-Gonzalez MA, Corella D, Estruch R, Ros E, Aros F, Schroder H, Fito M, et al. Legume consumption and risk of all-cause, cardiovascular, and cancer mortality in the PREDIMED study. Clinical nutrition (Edinburgh, Scotland) 2018. doi: 10.1016/j.clnu.2017.12.019.

27. Buckland G, Gonzalez CA, Agudo A, Vilardell M, Berenguer A, Amiano P, Ardanaz E, Arriola L, Barricarte A, Basterretxea M, et al. Adherence to the Mediterranean diet and risk of coronary heart disease in the Spanish EPIC Cohort Study. American journal of epidemiology 2009;170(12):1518-29. doi: 10.1093/aje/kwp282.

28. Bernstein AM, Sun Q, Hu FB, Stampfer MJ, Manson JE, Willett WC. Major dietary protein sources and risk of coronary heart disease in women. Circulation 2010;122(9):876-83. doi: 10.1161/circulationaha.109.915165.

29. Martinez-Gonzalez MA, Garcia-Lopez M, Bes-Rastrollo M, Toledo E, Martinez-Lapiscina EH, Delgado-Rodriguez M, Vazquez Z, Benito S, Beunza JJ. Mediterranean diet and the incidence of cardiovascular disease: a Spanish cohort. Nutrition, metabolism, and cardiovascular diseases : NMCD 2011;21(4):237-44. doi: 10.1016/j.numecd.2009.10.005.

30. Dilis V, Katsoulis M, Lagiou P, Trichopoulos D, Naska A, Trichopoulou A. Mediterranean diet and CHD: the Greek European Prospective Investigation into Cancer and Nutrition cohort. The British journal of nutrition 2012;108(4):699-709. doi: 10.1017/s0007114512001821.

31. Katsouyanni K, Rimm EB, Gnardellis C, Trichopoulos D, Polychronopoulos E, Trichopoulou A. Reproducibility and relative validity of an extensive semi-quantitative food frequency questionnaire using dietary records and biochemical markers among Greek schoolteachers. International journal of epidemiology 1997;26 Suppl 1:S118-27.

32. Haring B, Gronroos N, Nettleton JA, von Ballmoos MC, Selvin E, Alonso A. Dietary protein intake and coronary heart disease in a large community based cohort: results from the Atherosclerosis Risk in Communities (ARIC) study [corrected]. PloS one 2014;9(10):e109552. doi: 10.1371/journal.pone.0109552.

33. ARIC. Dietary Intake Form, version A, C. ARIC, 2019. Available from <https://www2.cscc.unc.edu/aric/cohort-forms>

34. Yu D, Zhang X, Gao YT, Li H, Yang G, Huang J, Zheng W, Xiang YB, Shu XO. Fruit and vegetable intake and risk of CHD: results from prospective cohort studies of Chinese adults in Shanghai. The British journal of nutrition 2014;111(2):353-62. doi: 10.1017/s0007114513002328.

35. Kelemen LE, Kushi LH, Jacobs DR, Jr., Cerhan JR. Associations of dietary protein with disease and mortality in a prospective study of postmenopausal women. American journal of epidemiology 2005;161(3):239-49. doi: 10.1093/aje/kwi038.

36. Wang JB, Fan JH, Dawsey SM, Sinha R, Freedman ND, Taylor PR, Qiao YL, Abnet CC. Dietary components and risk of total, cancer and cardiovascular disease mortality in the Linxian Nutrition Intervention Trials cohort in China. Scientific reports 2016;6:22619. doi: 10.1038/srep22619.

37. Mizrahi A, Knekt P, Montonen J, Laaksonen MA, Heliovaara M, Jarvinen R. Plant foods and the risk of cerebrovascular diseases: a potential protection of fruit consumption. The British journal of nutrition 2009;102(7):1075-83. doi: 10.1017/s0007114509359097.

38. Bernstein AM, Pan A, Rexrode KM, Stampfer M, Hu FB, Mozaffarian D, Willett WC. Dietary protein sources and the risk of stroke in men and women. Stroke 2012;43(3):637-44. doi: 10.1161/strokeaha.111.633404.

39. Misirli G, Benetou V, Lagiou P, Bamia C, Trichopoulos D, Trichopoulou A. Relation of the traditional Mediterranean diet to cerebrovascular disease in a Mediterranean population. American journal of epidemiology 2012;176(12):1185-92. doi: 10.1093/aje/kws205.

40. Haring B, Misialek JR, Rebholz CM, Petruski-Ivleva N, Gottesman RF, Mosley TH, Alonso A. Association of Dietary Protein Consumption With Incident Silent Cerebral Infarcts and Stroke: The Atherosclerosis Risk in Communities (ARIC) Study. Stroke 2015;46(12):3443-50. doi: 10.1161/strokeaha.115.010693.

41. Meyer KA, Kushi LH, Jacobs DR, Jr., Slavin J, Sellers TA, Folsom AR. Carbohydrates, dietary fiber, and incident type 2 diabetes in older women. The American journal of clinical nutrition 2000;71(4):921-30. doi: 10.1093/ajcn/71.4.921.

42. Hodge AM, English DR, O'Dea K, Giles GG. Glycemic index and dietary fiber and the risk of type 2 diabetes. Diabetes care 2004;27(11):2701-6.

43. Bazzano LA, Li TY, Joshipura KJ, Hu FB. Intake of fruit, vegetables, and fruit juices and risk of diabetes in women. Diabetes care 2008;31(7):1311-7. doi: 10.2337/dc08-0080.

44. Villegas R, Gao YT, Yang G, Li HL, Elasy TA, Zheng W, Shu XO. Legume and soy food intake and the incidence of type 2 diabetes in the Shanghai Women's Health Study. The American journal of clinical nutrition 2008;87(1):162-7. doi: 10.1093/ajcn/87.1.162.

45. Ericson U, Sonestedt E, Gullberg B, Hellstrand S, Hindy G, Wirfalt E, Orho-Melander M. High intakes of protein and processed meat associate with increased incidence of type 2 diabetes. The British journal of nutrition 2013;109(6):1143-53. doi: 10.1017/s0007114512003017.

46. Becerra-Tomas N, Diaz-Lopez A, Rosique-Esteban N, Ros E, Buil-Cosiales P, Corella D, Estruch R, Fito M, Serra-Majem L, Aros F, et al. Legume consumption is inversely associated with type 2 diabetes incidence in adults: A prospective assessment from the PREDIMED study. Clinical nutrition (Edinburgh, Scotland) 2018;37(3):906-13. doi: 10.1016/j.clnu.2017.03.015.

47. Khalili-Moghadam S, Mirmiran P, Bahadoran Z, Azizi F. The Mediterranean diet and risk of type 2 diabetes in Iranian population. European journal of clinical nutrition 2019;73(1):72-8. doi: 10.1038/s41430-018-0336-2.

48. Nunez-Cordoba JM, Valencia-Serrano F, Toledo E, Alonso A, Martinez-Gonzalez MA. The Mediterranean diet and incidence of hypertension: the Seguimiento Universidad de Navarra (SUN) Study. American journal of epidemiology 2009;169(3):339-46. doi: 10.1093/aje/kwn335.

49. Weng LC, Steffen LM, Szklo M, Nettleton J, Chambless L, Folsom AR. A diet pattern with more dairy and nuts, but less meat is related to lower risk of developing hypertension in middle-aged adults: the Atherosclerosis Risk in Communities (ARIC) study. Nutrients 2013;5(5):1719-33. doi: 10.3390/nu5051719.

50. Borgi L, Muraki I, Satija A, Willett WC, Rimm EB, Forman JP. Fruit and Vegetable Consumption and the Incidence of Hypertension in Three Prospective Cohort Studies. Hypertension (Dallas, Tex : 1979) 2016;67(2):288-93. doi: 10.1161/hypertensionaha.115.06497.

51. Golzarand M, Bahadoran Z, Mirmiran P, Azizi F. Protein Foods Group and 3-Year Incidence of Hypertension: A Prospective Study From Tehran Lipid and Glucose Study. Journal of renal nutrition : the official journal of the Council on Renal Nutrition of the National Kidney Foundation 2016;26(4):219-25. doi: 10.1053/j.jrn.2016.01.017.

52. Lelong H, Blacher J, Baudry J, Adriouch S, Galan P, Fezeu L, Hercberg S, Kesse-Guyot E. Individual and Combined Effects of Dietary Factors on Risk of Incident Hypertension: Prospective Analysis From the NutriNet-Sante Cohort. Hypertension (Dallas, Tex : 1979) 2017;70(4):712-20. doi: 10.1161/HYPERTENSIONAHA.117.09622.

53. Rautiainen S, Wang L, Lee IM, Manson JE, Buring JE, Sesso HD. Higher Intake of Fruit, but Not Vegetables or Fiber, at Baseline Is Associated with Lower Risk of Becoming Overweight or Obese in Middle-Aged and Older Women of Normal BMI at Baseline. The Journal of nutrition 2015;145(5):960-8. doi: 10.3945/jn.114.199158.
